# Inference for Stochastic Chemical Kinetics Using Moment Equations and System Size Expansion

**DOI:** 10.1371/journal.pcbi.1005030

**Published:** 2016-07-22

**Authors:** Fabian Fröhlich, Philipp Thomas, Atefeh Kazeroonian, Fabian J. Theis, Ramon Grima, Jan Hasenauer

**Affiliations:** 1 Helmholtz Zentrum München - German Research Center for Environmental Health, Institute of Computational Biology, Neuherberg, Germany; 2 Technische Universität München, Center for Mathematics, Chair of Mathematical Modeling of Biological Systems, Garching, Germany; 3 Department of Mathematics, Imperial College London, London, United Kingdom; 4 School of Biological Sciences, University of Edinburgh, Edinburgh, United Kingdom; University of Michigan, UNITED STATES

## Abstract

Quantitative mechanistic models are valuable tools for disentangling biochemical pathways and for achieving a comprehensive understanding of biological systems. However, to be quantitative the parameters of these models have to be estimated from experimental data. In the presence of significant stochastic fluctuations this is a challenging task as stochastic simulations are usually too time-consuming and a macroscopic description using reaction rate equations (RREs) is no longer accurate. In this manuscript, we therefore consider moment-closure approximation (MA) and the system size expansion (SSE), which approximate the statistical moments of stochastic processes and tend to be more precise than macroscopic descriptions. We introduce gradient-based parameter optimization methods and uncertainty analysis methods for MA and SSE. Efficiency and reliability of the methods are assessed using simulation examples as well as by an application to data for Epo-induced JAK/STAT signaling. The application revealed that even if merely population-average data are available, MA and SSE improve parameter identifiability in comparison to RRE. Furthermore, the simulation examples revealed that the resulting estimates are more reliable for an intermediate volume regime. In this regime the estimation error is reduced and we propose methods to determine the regime boundaries. These results illustrate that inference using MA and SSE is feasible and possesses a high sensitivity.

## Author Summary

In this manuscript, we introduce efficient methods for parameter estimation for stochastic processes. The stochasticity of chemical reactions can influence the average behavior of the considered system. For some biological systems, a microscopic, stochastic description is computationally intractable but a macroscopic, deterministic description too inaccurate. This inaccuracy manifests itself in an error in parameter estimates, which impede the predictive power of the proposed model. Until now, no rigorous analysis on the magnitude of the estimation error exists. We show by means of two simulation examples that using mesoscopic descriptions based on the system size expansions and moment-closure approximations can reduce this estimation error compared to inference using a macroscopic description. This reduction is most pronounced in an intermediate volume regime where the influence of stochasticity on the average behavior is moderately strong. For the JAK/STAT pathway where experimental data is available, we show that one parameter that was not structurally identifiable when using a macroscopic description becomes structurally identifiable when using a mesoscopic description for parameter estimation.

## Introduction

On the single-cell level many biological processes are influenced by stochastic fluctuations [1–3]. This stochasticity must be accounted for when constructing quantitative mechanistic models for the behavior of cells. Traditionally, dynamics of stochastic biochemical processes are modeled using the Chemical Master Equation (CME) [4]. The CME provides an accurate microscopic description of stochastic chemical kinetics [5] and enables the prediction of the behavior of biochemical reaction networks. To achieve high prediction accuracy, however, the parameters of the CME have to be inferred from experimental data. This inference is challenging and the development of new methods to perform efficient inference is the subject of current research.

**Fig 1:**
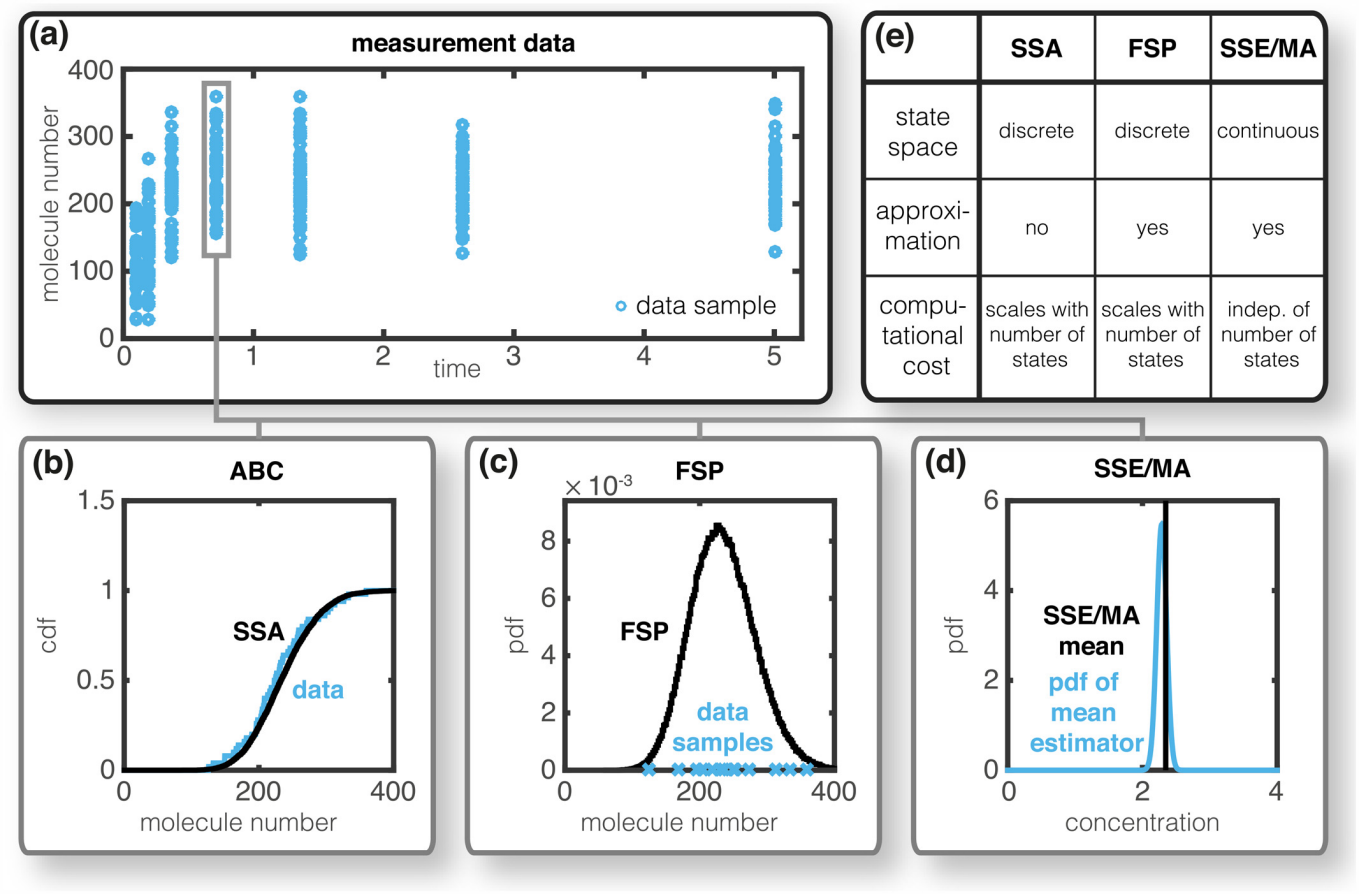
Inference methods for stochastic processes. (a) Single-cell snapshot data collected using a high-throughput technique, such as flow cytometry. (b) Empirical density functions for SSA runs (black —) and experimental data (blue —), the difference is used as distance measure in Approximate Bayesian Computing. (c) Instantaneous probability distribution computed using FSP (black —) to evaluate the likelihood of the observing the individual cells (blue ×). (d) Mean computed using MA/SSE (black —) as well as measured mean and its uncertainty (blue —). (e) Summary of the properties of the displayed methods.

In the literature, methods to perform statistical inference for single-cell time-lapse data [6–13] and populations snapshot data [14–20] have been proposed. These methods use the Stochastic Simulation Algorithm (SSA) [21], as well as various approximations of the CME such as the Finite State Projection (FSP) [22], moment closure approximations (MA) [23] and the linear-noise approximation (LNA) [24]. We next provide a brief discussion of these methods, in particular their use to infer the parameters from experimental single-cell data—a visual summary of these methods and their properties is provided in Fig 1. In this manuscript we will only consider population snapshot data and thus focus on the respective methods.

The parameters of stochastic processes are frequently inferred using Approximate Bayesian Computing approaches [25]. These methods rely on exhaustive stochastic simulations and accept parameter values if the differences between simulation and experimental data is sufficiently small [7, 13, 19]. While many methods which exploit stochastic simulations are asymptotically exact, their computational efficiency suffers from the required number of simulations. While SSA-based methods are asymptotically exact, appropriate stopping criteria and distance measures are difficult to obtain [26]. Furthermore, the computational efficiency of Approximate Bayesian Computing methods suffers from the tremendous number of required SSA runs.

Inference using FSP methods is usually more efficient than using the SSA [20]. The parameter dependent probability distribution of the process is simulated and the likelihood of the data under this distribution is evaluated (Fig 1b). This likelihood function is a multinomial probability distribution [15, 16] and efficient gradient-based optimization methods can be used [18]. The ODE systems might however be large and hence their simulation is intractable even when using state-of-the-art sliding window [27] and tensor train approaches [12]. Even with tailored methods [12, 27, 28], the simulation of many reaction networks remains computationally intractable and hence FSP-based inference is still very limited.

To circumvent the computational complexity of evaluating the full probability distribution, MA [29–32] and the SSE methods [24] have been introduced. Both classes of methods approximate the statistical moments of the stochastic process which is described by the CME:

- The MA is based on the hierarchy of evolution equations for the statistical moments of the CME solution. This hierarchy is truncated at an order *N* and the (*N* + 1)th order moments usually contained in the remaining system are approximated by functions of the lower-order moments. This approximation is based on an assumption of the distribution solution of the CME [33–35]. The *N*th order MA is in the following denoted by *N*MA.
- The SSE of the CME is a series expansion in the inverse volume of the compartment in which the system is confined [24]. The leading order in the mean gives the reaction rate equations (RRE) while the leading order in the variance gives the LNA. The consideration of additional terms in the expansion gives the expected mesoscopic rate equation (EMRE) [36] (the first-order correction to the RRE) and the inverse omega square (IOS) method [37] (the first-order correction to the LNA).

Both MA and SSE approaches generate a system of coupled ordinary differential equations (ODEs) for the approximate moments. It has been shown that the difference between MA and SSE methods decreases with increasing volume and approaches the solution of the CME [23]. The accuracy of MA equations and the conditions under which they provide physically meaningful results have recently been studied for several distribution choices [23, 33, 38].

For statistical inference of parameters the LNA and 2MA have recently been used [10, 17, 18, 39, 40]. The comparison of the measured and simulated moments often provides good parameter estimates [17, 18] and the corresponding estimation problems are tractable. Besides reducing the computational complexity, MA and SSE approaches also enable the application of techniques which were already established for deterministic models, e.g., structural identifiability analysis [41]. Accordingly, the literature for the application of MA and SSE methods for inference is promising, there is however plenty of room for improvement: (i) in none of the studies have gradient-based optimization methods with sensitivity equations been employed, even though they have been shown to be superior for a wide range of dynamical systems [42]; (ii) the estimation error of inferred parameter values is influenced by the fact that the MA and LNA typically provide an approximation of the moments for chemical systems with at least one bimolecular reaction (see [43] for more details on when the LNA is exact). Hence a systematic evaluation of estimation errors in the inferred parameter values, say as a function of the compartment volume is direly needed so that one can decide which modeling approach is best suited for a given compartment volume. (iii) it has been shown that EMRE and IOS yield more accurate approximations to the CME than possible using the LNA and RRE [36, 43–45] (although there are exceptions such as when the LNA is exact up to second-order moments with the CME [43]). Similarly in the limit of large volumes, it has been shown that higher-order MA equations are more accurate than lower-order ones [23]; for example the 3MA is more accurate than the commonly used 2MA. However to-date the equations derived by considering the terms in the SSE beyond the LNA and the equations obtained using the 3MA have not been used for inference.

In this manuscript, we will introduce an efficient gradient- and sensitivity-based method for parameter estimation for population snapshot data using MA and SSE-based approaches. This method is evaluated on experimental data available for the JAK/STAT signaling pathway model, which is traditionally modeled using the RRE. For this model, we demonstrate that our approach yields additional insight. Subsequent to this application part, we systematically evaluate the estimation error for two biochemical networks, each with at least one bimolecular reaction. We will provide a first quantification of the improvement achieved using the 3MA and the SSE truncated beyond the next to leading-order term over the RREs, 2MA and LNA. Using this evaluation, two simple approaches for the selection of the correct inference approach will be proposed.

## Methods

**Fig 2:**
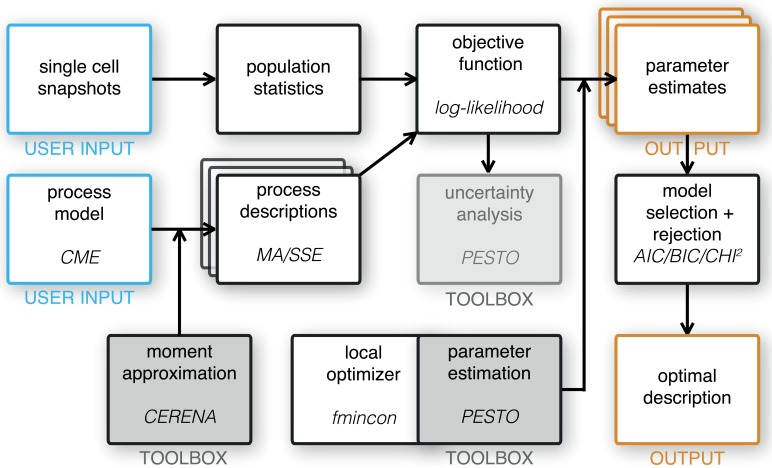
Workflow for modeling, parameter estimation and model selection. User inputs are colored in blue, workflow outputs are colored in orange. MATLAB toolboxes are indicated by gray boxes. The employed method/function/toolbox is indicated as oblique text in every box where applicable.

In the following we outline the considered modeling approaches, parameter estimation, uncertainty analysis, model selection. The workflow is shown in Fig 2.

### Chemical master equation

Consider a set of *R* reactions, involving *M* chemical species confined in a reaction volume of size Ω. Denoting the set of reactants by (*X*_1_, …, *X*_*M*_), the *r*^*th*^ reaction can be written as

$$\sum_{i=1}^{M}\nu_{ir}^{-}X_{i}\overset{k_{r}}{\to }\sum_{i=1}^{M}\nu_{ir}^{+}X_{i}.$$

Here *k*_*r*_ is the reaction rate constant, $\nu_{ir}^{\pm }$ are the integer stoichiometric coefficients, and we denote by $\nu_{ir}=\nu_{ir}^{+}-\nu_{ir}^{-}$ the change in molecules of the *i*^*th*^ species in the *r*^*th*^ reaction. Under well-mixed conditions the state of this biochemical system is characterized by the corresponding vector of molecule numbers *n* = (*n*_1_, …, *n*_*M*_). The time-evolution of the probability of observing the system in state *n*, then obeys the CME

$$\frac{dP(n,t)}{dt}=\Omega \sum_{r=1}^{R} [{\hat{f}}_{r}\left(n-\nu_{r}\right)P\left(n-\nu_{r},t\right)-{\hat{f}}_{r}\left(n\right)P\left(n,t\right)].$$ (1)

Here, *ν*_*r*_ denotes the stoichiometry (*ν*_1*r*_, …, *ν*_*Mr*_) of the *r*^*th*^ reaction and $\Omega {\hat{f}}_{r}\left(n\right)$ is the propensity function, i.e., the probability per unit time for reaction *r* to occur somewhere in the volume Ω. Since the CME is often intractable for analytical solution, we here focus on approximation methods for the mean concentrations *μ*_*i*_ = 〈*n*_*i*_/Ω〉, and the corresponding covariances of the concentration fluctuations about them, Σ_*ij*_ = 〈(*n*_*i*_/Ω − *μ*_*i*_)(*n*_*j*_/Ω − *μ*_*j*_)〉, which is outlined in the following.

### Moment-closure approximation

Equations for the moments are straightforwardly derived from the CME Eq (1). For systems involving non-linear propensities, however, these equations are intractable because the equation for a certain moment is typically coupled to higher-order moments resulting in an infinite system of equations. A common procedure to break this hierarchy of moment equations is to neglect higher than second order cumulants [29]; this indeed is the same as assuming that the third order cumulant is consistent with a Gaussian distribution. Assuming at most bimolecular reactions, the result is a set of non-linear ODEs coupling mean and variance called the 2MA and is given by

$$\begin{array}{lll} \frac{\partial \mu_{i}}{\partial t} & = & \sum_{r=1}^{R}\nu_{ir}\left({\hat{f}}_{r}(\Omega \mu )+\frac{1}{2}\sum_{s,l=1}^{M}\frac{\partial^{2}{\hat{f}}_{r}(\Omega \mu )}{\partial \mu_{s}\partial \mu_{l}}\Sigma_{sl}\right),\\ \frac{\partial \Sigma_{ij}}{\partial t} & = & \sum_{r=1}^{R}\sum_{s=1}^{M}\left(\nu_{ir}\frac{\partial {\hat{f}}_{r}(\Omega \mu )}{\partial \mu_{s}}\Sigma_{sj}+\nu_{jr}\frac{\partial {\hat{f}}_{r}(\Omega \mu )}{\partial \mu_{s}}\Sigma_{si}\right)\\ & & +\frac{1}{\Omega }\sum_{r=1}^{R}\nu_{ir}\nu_{jr}\left({\hat{f}}_{r}(\Omega \mu )\;+\sum_{s,l=1}^{M}\frac{\partial^{2}{\hat{f}}_{r}(\Omega \mu )}{\partial \mu_{s}\partial \mu_{l}}\Sigma_{sl}\right). \end{array}$$

The 2MA is precise for unimolecular reactions and fairly accurate if the third order moment is negligible [29]. The latter is mostly the case for large reaction volume and molecule numbers [29]. For small volumes higher-order moment equation must be used. Neglecting higher than third order cumulants yields the 3rd order moment-closure approximation (3MA) that are outlined in Ref. [29, 30]. The simulation routines were generated using the CERENA toolbox [46].

### System size expansion

A different technique to approximate the moments of the CME is given by the SSE. The procedure allows us to expand the CME about the solution of the RREs which are valid for large reaction volumes Ω and are given by

$$\frac{\partial \phi_{i}}{\partial t}=\sum_{r=1}^{R}\nu_{ir}f_{r}\left(\phi \right)\;.$$

Here $f_{j}\left(\phi \right)=\lim_{\Omega \to \infty \;}\hat{f_{j}}\left(\Omega \phi \right)$ denotes the macroscopic rate function. While the RREs represent the leading order term of the SSE and yield the average concentrations for large volumes Ω, the next term, the LNA, describes the fluctuations about these concentrations. The covariance of these fluctuations obeys [24, 47]:

$$\frac{\partial \Sigma_{ij}}{\partial t}=\sum_{r=1}^{R}\sum_{s=1}^{M}\left(\nu_{ir}\frac{\partial f_{r}\left(\phi \right)}{\partial \phi_{s}}\Sigma_{sj}+\nu_{jr}\frac{\partial f_{r}\left(\phi \right)}{\partial \phi_{s}}\Sigma_{si}\right)+\frac{1}{\Omega }\sum_{r=1}^{R}\nu_{ir}\nu_{jr}f_{r}\left(\phi \right).$$

These results are exact for reaction networks comprising up to unimolecular reactions and for a small subset of networks with bimolecular reactions [43]. For most networks involving bimolecular reactions, the SSE enables us to systematically correct the mean concentrations of the RREs and the variance predictions of the LNA, by considering higher order terms in the expansion. A more accurate estimate for the mean concentrations than the RREs is given by the EMRE [36], and follows

$$\frac{\partial \mu_{i}}{\partial t}=\frac{\partial \phi_{i}}{\partial t}+\sum_{r=1}^{R}\nu_{ir}\left(\sum_{s=1}^{M}\frac{\partial f_{r}\left(\phi \right)}{\partial \phi_{s}}\left(\mu_{s}-\phi_{s}\right)+\frac{1}{2}\sum_{s,l=1}^{M}\frac{\partial^{2}f_{r}\left(\phi \right)}{\partial \phi_{s}\phi_{l}}\Sigma_{sl}-\frac{1}{2}\sum_{s=1}^{M}\frac{\phi_{s}}{\Omega }\frac{\partial^{2}f_{r}\left(\phi \right)}{\partial \phi_{s}^{2}}\right).$$

Note that these equations yield a correction term of order Ω^−1^ to the RREs. Correspondingly, expressions for the covariances about these more accurate concentrations can be derived using the IOS approximation, which corrects the LNA estimate to order Ω^−2^ [37]. In contrast to RRE and LNA, EMRE and IOS do not assume large volumes and hence these estimates are expected to be closer to the true moments predicted by the CME.

In what follows we shall collectively refer to the EMRE and IOS as higher-order SSEs, meaning they are obtained using the SSE truncated to a higher-order than that giving the LNA. The simulation routines were generated using the CERENA toolbox [46].

### Statistical model of experimental data

In this study we consider population average data as well as single-cell snapshot data. Population average data could, among others, be obtained by Western blot and (bulk) mRNA sequencing. Single-cell snapshot data could be obtained by flow and mass cytometry. Some statistical properties of these data types are introduced in the following.

#### Population average data

These data provide information about the mean *μ*_*i*_(*t*_*k*_, *θ*) of measured quantities ${\hat{\mu }}_{i,k}$ at times *t*_*k*_,

$${\hat{\mu }}_{i,k}=\mu_{i}\left(t_{k},\theta \right)+\epsilon_{i,k,T}\;.$$

These measurements are noise corrupted. The measurement noise *ϵ*_*i*, *k*, T_ is in the following assumed to be independently and distributed with mean zero and variance $\sigma_{{\hat{\mu }}_{i,k},T}^{2}$, $\epsilon_{i,k,T}∼N\left(0,\sigma_{{\hat{\mu }}_{i,k},T}^{2}\right)$ and true population mean *μ*_*i*_(*t*_*k*_, *θ*).

#### Single-cell snapshot data

These data provide information about the measured quantities *y*_*i*_ at times *t*_*k*_ for individual cells. The single cell measurements are given by

$${\hat{y}}_{i,k}^{\left(j\right)}=y_{i,k}^{\left(j\right)}+\epsilon_{i,k,T},\;j=1,\ldots ,N\;,$$

with $y_{i,k}^{\left(j\right)}$ denoting a sample from the cell population, $y_{i,k}^{\left(j\right)}∼p\left(y_{i}|t_{k},\theta \right)$ with mean *μ*_*i*_(*t*_*k*_, *θ*), variance Σ_*ii*_(*t*_*k*_, *θ*) and fourth order central moment Σ_*iiii*_(*t*_*k*_, *θ*). The technical noise is assumed to depend on the replicate and therefore independent of *j*. From these samples mean and variances,

$${\hat{\mu }}_{i,k}=\frac{1}{N}\sum_{j=1}^{N}{\hat{y}}_{i,k}^{\left(j\right)}\;\text{and}\;{\hat{\Sigma }}_{ii,k}=\frac{1}{N}\sum_{j}^{N}{\left({\hat{y}}_{i,k}^{\left(j\right)}-{\hat{\mu }}_{i,k}\right)}^{2}\;,$$

as well as higher-order moments can be estimated. According to the central limit theorem, these estimators are approximately normally distributed for *N* ≫ 1. The estimator of the mean, ${\hat{\mu }}_{i,k}$, possesses the variance

$$\sigma_{{\hat{\mu }}_{i,k}}^{2}=E [{\left({\hat{\mu }}_{i,k}-\mu_{i}\left(t_{k},\theta \right)\right)}^{2}]=\underset{=\;\text{statistical}\;\text{uncertainty}}{\underset{︸}{E [{\left(\frac{1}{N}\sum_{j=1}^{N}y_{i,k}^{\left(j\right)}-\mu_{i}\left(t_{k},\theta \right)\right)}^{2}]}}+\underset{=\;\text{technical}\;\text{noise}}{\underset{︸}{E [\epsilon_{i,k,T}^{2}]}},$$

where the last reformulation exploits independence of $y_{i,k}^{\left(j\right)}$ and $\epsilon_{i,k,T}^{2}$. The first summand has the value $\frac{1}{N}\Sigma_{ii,k}$ (see [17, 48]) and describe the statistical noise resulting from the finite number of measured cells. As the sample size *N* grows, this variance contribution goes to zeros. In contrast, the second summand is the variance of the technical noise, $\sigma_{{\hat{\mu }}_{i,k},T}^{2}$, which is independent of the sample size. This yields the overall variance

$$\sigma_{{\hat{\mu }}_{i,k}}^{2}=\frac{1}{N}\Sigma_{ii}\left(t_{k},\theta \right)+\sigma_{{\hat{\mu }}_{i,k},T}^{2},$$

The estimator of the variance, ${\hat{\Sigma }}_{ii,k}$, possesses the variance

$$\sigma_{{\hat{\Sigma }}_{ii,k}}^{2}=E [{\left({\hat{\Sigma }}_{ii,k}-\Sigma_{ii}\left(t_{k},\theta \right)\right)}^{2}]=\frac{1}{N}\left(\Sigma_{iiii}\left(t_{k},\theta \right)-\frac{N-3}{N-1}\Sigma_{ii}^{2}\left(t_{k},\theta \right)\right)\;,$$

which is independent of the technical noise. For a detailed derivation we refer the reader to the supplement. Note that the estimates of mean and variance are potentially correlated if both are computed from the same sample [48].

The statistical description of population snapshot data also provides a framework for population average data. Experimental techniques providing population average typically analyze millions of single cells simultaneously. Accordingly, *N* is rather large, yielding the variance $\sigma_{{\hat{\mu }}_{i,k}}^{2}\approx \sigma_{{\hat{\mu }}_{i,k},T}^{2}$.

#### Modeling of noise variance

The variance of mean and variance estimators, $\sigma_{{\hat{\mu }}_{i,k}}^{2}$ and $\sigma_{{\hat{\Sigma }}_{ii,k}}^{2}$ depends on the statistical moments of the process and the variance of the technical noise. The moments Σ_*ii*_(*t*_*k*_, *θ*) and Σ_*iiii*_(*t*_*k*_, *θ*) could be computed using higher-order MA and SSE. However, this can be computationally intensive and subject to approximation errors. Instead, we used the sample-based estimates of these statistical moments, ${\hat{\Sigma }}_{ii,k}=\frac{1}{N}\sum_{j}^{N}{\left({\hat{y}}_{i,k}^{\left(j\right)}-{\hat{\mu }}_{i,k}\right)}^{2}$ and ${\hat{\Sigma }}_{iiii,k}=\frac{1}{N}\sum_{j}^{N}{\left({\hat{y}}_{i,k}^{\left(j\right)}-{\hat{\mu }}_{i,k}\right)}^{4}$. These estimates are rather reliable (for *N* ≫ 1) and are not influenced by technical noise. Accordingly, the variance of the technical noise, $\sigma_{{\hat{\mu }}_{i,k},T}^{2}$, can either be obtained by computing the statistics over multiple experimental replicates with large sample sizes (*N* ≫ 1), or by modeling them as a possibly parameter dependent function. For generality, we assume in the following that the variances of the estimators are parameter dependent, $\sigma_{{\hat{\mu }}_{i,k}}^{2}\left(\theta \right)$ and $\sigma_{{\hat{\Sigma }}_{ii,k}}^{2}\left(\theta \right)$.

### Parameter estimation

To infer the parameters of biochemical reaction networks we employ maximum likelihood and Bayesian parameter estimation. Based upon the statistical model introduced above, the likelihood function becomes

$$\begin{array}{ll} L\left(\theta \right) & =\underset{(1)\text{ likelihood of measured means}}{\underset{︸}{\underset{i,k}{\prod }\frac{1}{\sqrt{2\pi \sigma_{{\hat{\mu }}_{i,k}}^{2}\left(\theta \right)}}\text{exp}\left(-\frac{1}{2}{\left(\frac{\mu_{i}\left(t_{k},\theta \right)-{\hat{\mu }}_{i,k}}{\sigma_{{\hat{\mu }}_{i,k}}\left(\theta \right)}\right)}^{2}\right)}}\\ & \;\;\times \underset{(2)\text{ likelihood of measured variances}}{\underset{︸}{\underset{i,k}{\prod }\frac{1}{\sqrt{2\pi \sigma_{{\hat{\Sigma }}_{ii,k}}^{2}\left(\theta \right)}}\text{exp}\left(-\frac{1}{2}{\left(\frac{\Sigma_{ii}\left(t_{k},\theta \right)-{\hat{\Sigma }}_{ii,k}}{\sigma_{{\hat{\Sigma }}_{ii,k}}\left(\theta \right)}\right)}^{2}\right)}}. \end{array}$$

The two contributions, (1) and (2), provide the likelihood of measured mean and measured variance of the data, respectively. In the absence of information about the variance, part (2) is set to one. To improve the numerical robustness and the convergence properties of optimizers, instead of maximizing the likelihood, the negative log-likelihood

$$\begin{array}{cc} J\left(\theta \right) & =\frac{1}{2}\underset{k,i}{\sum }\left(\text{log}2\pi \sigma_{{\hat{\mu }}_{i,k}}^{2}\left(\theta \right)+{\left(\frac{\mu_{i}\left(t_{k},\theta \right)-{\hat{\mu }}_{i,k}}{\sigma_{{\hat{\mu }}_{i,k}}\left(\theta \right)}\right)}^{2}\right)\\ & \;\;+\frac{1}{2}\underset{k,i}{\sum }\left(\text{log}2\pi \sigma_{{\hat{\Sigma }}_{ii,k}}^{2}\left(\theta \right)+{\left(\frac{\Sigma_{ii}\left(t_{k},\theta \right)-{\hat{\Sigma }}_{ii,k}}{\sigma_{{\hat{\Sigma }}_{ii,k}}\left(\theta \right)}\right)}^{2}\right) \end{array}$$

is minimized [42]. The corresponding minimization problem is

$$\hat{\theta }=\arg\min_{\theta \in \Theta }J\left(\theta \right)\;,$$

with plausible parameter domain Θ. The minimizer $\hat{\theta }$ of *J*(*θ*) is the maximum likelihood estimate. In practice, a further improvement is often achieved by optimizing the log-transformed parameter *ξ* = log *θ* instead of *θ* [42].

The optimization of the objective function has been implemented in MATLAB using our in-house Parameter Estimation Toolbox (PESTO). PESTO uses a multi-start local optimization scheme, an approach which has been shown to perform well for similar problems [42]. To ensure a good coverage of the domain Θ [42], the starting points for the local solvers were generated using a latin hypercube sampling between the lower and upper bounds for the parameters defined by Θ. In order to exploit gradient and curvature information in the local optimization we made use of the trust-region-reflective algorithm [49, 50] implemented in the MATLAB routine fmincon.m.

The gradient of the objective function with respect to parameter *θ*_*l*_ is given by

$$\begin{array}{l} \;\frac{\partial J}{\partial \theta_{l}}=\sum_{i,k}\frac{1}{\sigma_{{\hat{\mu }}_{i,k}}^{2}(\theta )}\left(1-{\left(\frac{\mu_{i}(t_{k},\theta )-{\hat{\mu }}_{i,k}}{\sigma_{{\hat{\mu }}_{i,k}}^{2}(\theta )}\right)}^{2}\right){\frac{\partial \sigma_{{\hat{\mu }}_{i,k}}^{2}}{\partial \theta_{l}}|}_{\theta }+\frac{\mu_{i}(t_{k},\theta )-{\hat{\mu }}_{i,k}}{\sigma_{{\hat{\mu }}_{i,k}}^{2}(\theta )}{\frac{\partial \mu_{i}}{\partial \theta_{l}}|}_{t_{k},\theta }\\ +\sum_{i,k}\frac{1}{\sigma_{{\hat{\Sigma }}_{ii,k}}^{2}(\theta )}\left(1-{\left(\frac{\Sigma_{i}(t_{k},\theta )-{\hat{\Sigma }}_{ii,k}}{\sigma_{{\hat{\mu }}_{i,k}}^{2}(\theta )}\right)}^{2}\right){\frac{\partial \sigma_{{\hat{\Sigma }}_{i,k}}^{2}}{\partial \theta_{l}}|}_{\theta }+\frac{\Sigma_{ii}(t_{k},\theta )-{\hat{\Sigma }}_{i,k}}{\Sigma_{{\hat{\mu }}_{i,k}}^{2}(\theta )}{\frac{\partial \Sigma_{ii}}{\partial \theta_{l}}|}_{t_{k},\theta }, \end{array}$$

in which $\partial \mu_{i}/\partial \theta_{l}≔{\left(s_{l}^{\mu }\right)}_{i}$ and $\partial \Sigma_{ii}/\partial \theta_{l}≔{\left(s_{l}^{\Sigma }\right)}_{i}$ denote the sensitivity of mean and variance with respect to the parameters. The governing equations for the sensitivities $s_{l}^{\mu }$ and $s_{l}^{\Sigma }$ are derived by differentiation of the evolution equations and subsequent reordering. For the 2MA the sensitivities are governed by:

$$\begin{array}{c} \frac{\partial s_{l}^{\mu }}{\partial t}\;=\;\left(\frac{\partial }{\partial \mu }\left(\frac{\partial \mu }{\partial t}\right)\right)s_{l}^{\mu }+\left(\frac{\partial }{\partial \Sigma }\left(\frac{\partial \mu }{\partial t}\right)\right)s_{l}^{\Sigma }+\frac{\partial }{\partial \theta_{l}}\left(\frac{\partial \mu }{\partial t}\right),\\ \frac{\partial s_{l}^{\Sigma }}{\partial t}\;=\;\left(\frac{\partial }{\partial \mu }\left(\frac{\partial \Sigma }{\partial t}\right)\right)s_{l}^{\mu }+\left(\frac{\partial }{\partial \Sigma }\left(\frac{\partial \Sigma }{\partial t}\right)\right)s_{l}^{\Sigma }+\frac{\partial }{\partial \theta_{l}}\left(\frac{\partial \Sigma }{\partial t}\right),\; \end{array}$$

in which ∂*μ*/∂*t* and ∂Σ/∂*t* denote the right-hand side of the evolution equations for the 2MA. For the EMRE the sensitivities are governed by:

$$\begin{array}{l} \frac{\partial s_{l}^{\Phi }}{\partial t}\;=\;\left(\frac{\partial }{\partial \Phi }\left(\frac{\partial \Phi }{\partial t}\right)\right)s_{l}^{\Phi }+\left(\frac{\partial }{\partial \mu }\left(\frac{\partial \Phi }{\partial t}\right)\right)s_{l}^{\mu }+\left(\frac{\partial }{\partial \Sigma }\left(\frac{\partial \Phi }{\partial t}\right)\right)s_{l}^{\Sigma }+\frac{\partial }{\partial \theta_{l}}\left(\frac{\partial \Phi }{\partial t}\right),\\ \frac{\partial s_{l}^{\mu }}{\partial t}\;=\;\left(\frac{\partial }{\partial \Phi }\left(\frac{\partial \mu }{\partial t}\right)\right)s_{l}^{\Phi }+\left(\frac{\partial }{\partial \mu }\left(\frac{\partial \mu }{\partial t}\right)\right)s_{l}^{\mu }+\left(\frac{\partial }{\partial \Sigma }\left(\frac{\partial \mu }{\partial t}\right)\right)s_{l}^{\Sigma }+\frac{\partial }{\partial \theta_{l}}\left(\frac{\partial \mu }{\partial t}\right),\\ \frac{\partial s_{l}^{\Sigma }}{\partial t}\;=\;\left(\frac{\partial }{\partial \Phi }\left(\frac{\partial \Sigma }{\partial t}\right)\right)s_{l}^{\Phi }+\left(\frac{\partial }{\partial \mu }\left(\frac{\partial \Sigma }{\partial t}\right)\right)s_{l}^{\mu }+\left(\frac{\partial }{\partial \Sigma }\left(\frac{\partial \Sigma }{\partial t}\right)\right)s_{l}^{\Sigma }+\frac{\partial }{\partial \theta_{l}}\left(\frac{\partial \Sigma }{\partial t}\right), \end{array}$$

in which $\partial s_{l}^{\Phi }=\partial \Phi /\partial \theta_{l}$ is the sensitivity of the solution of the reaction rate equation and ∂Φ/∂*t*, ∂*μ*/∂*t* and ∂Σ/∂*t* denote the right-hand side of the evolution equations for the EMRE. The sensitivity equations for RRE, 3MA and IOS possess a similar structure as those for 2MA and EMRE. In principle all the sensitivity equations can be obtained by rewriting the respective systems into systems of ODEs and using generic methods (see, e.g., [51]).

The gradient of the objective function was computed using forward sensitivity equations to ensure robust and efficient evaluation [42]. In addition to gradient information, we supplied fmincon.m with the Fisher-Information Matrix as approximation to the Hessian of the objective function to accelerate the optimization. This approximation of the Hessian is equivalent to the formulation in Levenberg-Marquardt [52] type optimization schemes. Parameter and objective function tolerances were both set to 10^−6^. For every dataset, the multi-start scheme was initialized at 50 initial values using a latin hypercube sampling. Convergence to a local and supposedly global optimum was checked by ensuring that a minimum of 5 of the 50 starts yielded the same minimal objective function value. If convergence was not observed, we doubled the number of multi-starts until this criterion was met.

### Uncertainty analysis

Experimental data of biochemical processes is often scarce and noise corrupted, resulting in non-identifiabilities and parameter uncertainties. Parameter identifiability is typically assessed using structural and practical identifiability analysis (see [41, 53] and references therein). Structural identifiability analysis provides information for the considered model topology and measured output, independent of a specific dataset. In contrast, practical identifiability and uncertainty analysis provide information about the reliability of parameter estimates for a given dataset. In this study we use profile likelihoods [54, 55] and Bayesian methods [56, 57] to study practical identifiability and parameter uncertainties.

The profile likelihood of a parameter *θ*_*i*_, denoted by PL(*θ*_*i*_), is given by the likelihood maximized over the remaining parameters,

$$\text{PL}\left(\theta_{i}\right)=\max_{\theta_{j\;\neq \;i},\theta \in \Theta }L\left(\theta \right).$$

Accordingly, profile likelihoods can be computed by solving a set of constrained optimization problems requiring repeated local optimization. In this study this task was carried out using the toolbox PESTO. Frequentist confidence intervals can be computed by comparing the profile likelihood PL(*θ*_*i*_) to the likelihood $L(\hat{\theta })$ at the globally optimal parameter point $\hat{\theta }$ [58]. As the models considered here can contain structurally non-identifiable parameters, profile likelihoods are the only viable frequentist technique for global uncertainty analysis [59].

Bayesian uncertainty analysis methods rely on Bayes’ theorem,

$$p\left(\theta |D\right)=\frac{p(D|\theta )p(\theta )}{p(D)},$$

in which *p*(*θ*), p(D|θ)(=L(θ)), p(D) and p(θ|D) denote prior probability, likelihood, evidence and posterior distribution, respectively [56]. For determining Bayesian credibility intervals of the parameters, we sampled from the posterior distribution using the efficient adaptive Markov Chain Monte Carlo (MCMC) method delayed rejection adaptive metropolis [60]. From the multivariate samples the respective univariate Bayesian confidence intervals were computed. We collected a total of 10^5^ samples after a burn-in period of 10^4^. In accordance with the log-transformed parameters used for optimization, a log-uniform prior over the parameter domain Θ has been employed.

### Model selection

For comparing competing model alternatives, we used Akaike’s Information Criterion (AIC),

$${\text{AIC}}_{l}=-2\log L\left({\hat{\theta }}_{l}\right)+2n_{\theta ,l}\;.$$

The AIC of the *l*-th model depends on the maximum of the likelihood, ${\hat{\theta }}_{l}$, and the number of estimated parameters *n*_*θ*, *l*_. Therefore, the AIC accounts for the match of model and data as well as for model complexity. The model with the lowest AIC value and index l* is selected. In order to simplify the interpretation of individual AIC values, we employ Akaike weights [61] defined by

$$w_{\text{AIC},l}=\frac{\exp\left(-\frac{1}{2}\left({\text{AIC}}_{l}-{\text{AIC}}_{l^{*}}\right)\right)}{\sum_{l^{\prime }}\exp\left(-\frac{1}{2}\left({\text{AIC}}_{l^{\prime }}-{\text{AIC}}_{l^{*}}\right)\right)}.$$

The AIC weight *w*_AIC, *l*_ of the *l*-th model is related to its posterior probability [61].

Reliability of our results has been ensured by comparing these values to the Bayesian information criterion (BIC) [62] and their corresponding BIC weights. As the number of parameters of the different models (e.g., RRE, EMRE and 2MA) is very similar, the results of these model selection criteria were comparable.

### Model falsification

Model selection criteria provide information about the relative quality of competing models, but not about their respective goodness-of-fit. The best model l* may still fail to adequately describe the measured data. To assess whether a model fits the data appropriately, we considered the sum of squared residuals at the optimal parameter value $\hat{\theta }$ [63],

$$\chi^{2}\left(\hat{\theta }\right)=\sum_{i,k}\underset{≔r_{\mu_{i,k}}^{2}}{\underset{︸}{{\left(\frac{\mu_{i}\left(t_{k},\hat{\theta }\right)-{\hat{\mu }}_{i,k}}{\sigma_{{\hat{\mu }}_{i,k}}\left(\hat{\theta }\right)}\right)}^{2}}}+\sum_{i,k}\underset{≔r_{\Sigma_{ii,k}}^{2}}{\underset{︸}{{\left(\frac{\Sigma_{ii}\left(t_{k},\hat{\theta }\right)-{\hat{\Sigma }}_{ii,k}}{\sigma_{{\hat{\Sigma }}_{ii,k}}\left(\hat{\theta }\right)}\right)}^{2}}}\;.$$

The sum of squared residuals is a standard goodness-of-fit statistic and is equal to $-2\log L(\hat{\theta })$ put to a negative constant. As for adequate models the residuals *r*_*μ*_*i*, *k*__ and *r*_Σ_*ii*, *k*__ should be normally distributed with unit variance, the sum of squared residuals should be drawn from a *χ*^2^-distribution [64]. The number of degrees of freedom of the *χ*^2^ distribution is the number of data points minus number of parameters. Accordingly, the *χ*^2^-test can be used for model rejection [65, 66].

## Results

In the following, we will illustrate how MA and SSE can be used to infer the parameters of stochastic biochemical processes. We will outline how the results can be interpreted and tested, and which novel insights can be gained even when only population-average data is available. For this purpose, we study an example for which experimental data is available and two examples for which artificial data was generated using stochastic simulations. The application to experimental data should substantiate the relevance of the developed methods in real-world application whereas the application to simulation examples allows for a more detailed analysis of the method properties.

### Application to experimental data: The JAK/STAT signaling pathway

To evaluate MA and SSE based inference in a real-world application, we study the dynamics of the Janus family of kinases (JAK)-signal transducer and activator of transcription (STAT) signaling pathway [67]. Constitutive activation of STATs is related to the malignancy of many tumors [68]. Moreover, Erythropoietin (Epo), the upstream activation factor of the JAK/STAT signaling pathway, is administered as therapeutic agent for treatment of cancer related anaemia [69]. This is the case although several adverse effects such as increased tumour progression and thromboembolic events have been attributed to Epo [69, 70].

**Fig 3:**
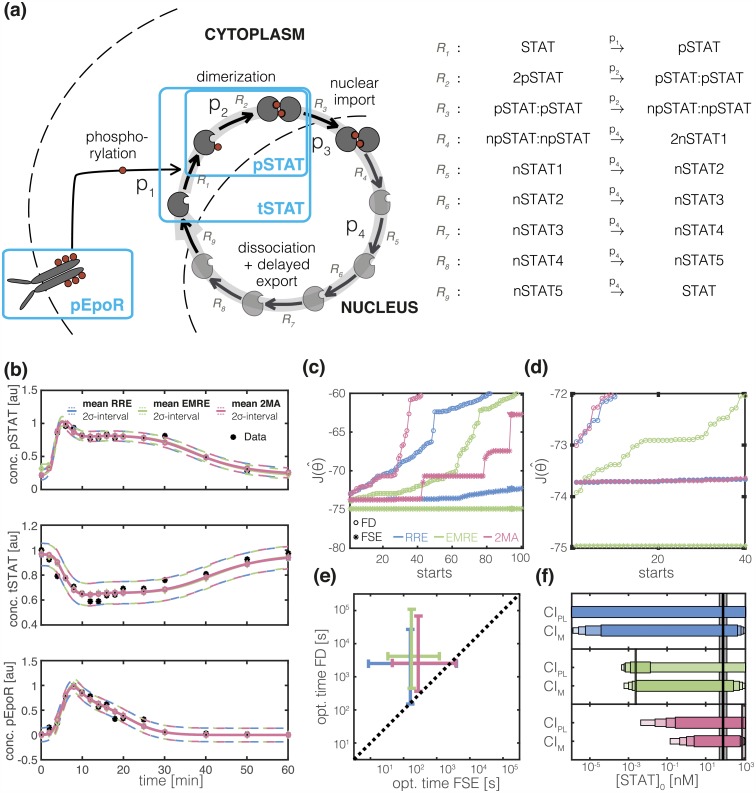
Parameter inference using EMRE and 2MA for JAK/STAT signaling pathway. (a) Schematic of JAK/STAT signaling pathway including biochemical reactions (→), biochemical species (gray elements) and observed outputs (blue boxes). Elements introduced to capture the delayed export of pSTAT from the nucleus are indicates as light gray. For subplots (b)-(e): RRE (blue), EMRE (green) and 2MA (red). (b) Experimental data (*), fitted mean (—) and estimated 2*σ* interval of the measurement noise (- -). (c) Objective function values for the best 100 (out of 1000) multi-starts obtained using forward sensitivity analysis (FSE, *) and finite differences (FD, °) for gradient calculation. Local optimization for RRE, EMRE and 2MA used the same initial parameter values. (d) Zoom-in of the 40 best multi-starts. (e) Median (+) and 80% percentile interval of computation time per local optimizer run. (f) Estimate of initial STAT concentration. Vertical lines mark the maximum likelihood estimates and the horizontal bars represent the confidence(CI_PL_)/credibility(CI_M_) intervals corresponding to different significance levels (80%, 90%, 95% and 99%) computed using profile likelihoods/MCMC samples. The reference value with 95% confidence intervals [71] is depicted by a black line and gray bar respectively.

The core module of the JAK/STAT signaling pathway is composed of the Erythropoietin receptor (EpoR) and the transcription factor STAT5. Upon phosphorylation, the Epo receptor induces phosphorylation of STAT5 via the JAK2 kinase. Phosphorylated STAT5 (pSTAT) can dimerize and the pSTAT dimer can translocate to the nucleus to activate the transcription of target genes. The dimer dissociates and is exported to the cytoplasm after some delay, which is described by a sequence of intermediate states. The biochemical reaction network is depicted in Fig 3(a). A more detailed description of the employed mathematical model is provided in S1 Supporting Information Section 1.2.

The JAK/STAT signaling pathway is a well studied system [53, 67]. For inference, we use Western Blot data for the phosphorylated Epo receptor (pEpoR), the cytoplasmic phosphorylated STAT (pSTAT), and the cytoplasmic STAT (tSTAT). These Western Blots average concentrations in thousands of cells, thereby provide information about the population mean but not about cell-to-cell variability. Due to the large cell numbers, statistic uncertainty can be ignored ($\frac{1}{N}\Sigma_{ii}\left(t_{k},\theta \right)=0$). The technical noise of each measured species was estimated as additional log-scaled parameters ($\sigma_{{\hat{\mu }}_{\text{pSTAT},k},T}^{2}=10^{\theta_{15}}$,$\sigma_{{\hat{\mu }}_{\text{tSTAT},k},T}^{2}=10^{\theta_{16}}$,$\sigma_{{\hat{\mu }}_{\text{pEpoR},k},T}^{2}=10^{\theta_{17}}$). The data have been recorded by Swameye et al. [67] and are depicted by the black stars in Fig 3(b).

#### Mesoscopic description of the JAK/STAT signaling

A RRE model for the JAK/STAT signaling pathway has been introduced by Swameye et al. [67] and analyzed/extended in subsequent publications [53, 71]. Microscopic and mesoscopic descriptions of the process have not been studied yet. Thus, it remains unclear which role stochasticity plays in this process and how valid the RRE description is. To address this, we derived 2MA and EMRE models for the process (S1 Code). As the JAK/STAT pathway involves two compartments, the cytoplasm and the nucleus, we applied a simple extension of the MA and SSE to multiple compartments (see SI for details). The extension essentially leads to a rescaling of propensities for reactions that transport chemical species between compartments and ensures the correctness of parameter estimates of the associated kinetic rates.

The 2MA and EMRE models are studied along with the well-known RRE model by Raue et al. [53]. All three descriptions possess 5 mechanistic parameters: 4 kinetic parameters (*p*_1_, …, *p*_4_); and the initial concentration of STAT5 in the cytoplasm ([STAT]_0_). For all descriptions, the pEpoR concentration is modeled as a time-dependent cubic spline function with 5 parameters. Furthermore, 7 nuisance parameters are used, i.e. scaling factors, noise variances. The number of state variables for RRE, EMRE and 2MA are 8, 52 and 44, respectively. As the dimerization reaction possesses a nonlinear propensity, the predictions for the mean of the underlying stochastic process differ between the models. Moreover, the phosphorylation of STAT5 depends on the pEpoR concentration which, as the concentration is modeled as a spline function, gives rise to a time-dependent propensity.

#### Efficient multi-start local optimization makes parameter inference feasible

As parameter estimation for RRE was reported to be challenging [53, 55], we evaluated multi-start local optimization for 2MA, EMRE and RRE using a large number of multi-starts (1000). Similar to previous studies we used the trust-region-reflective method in the MATLAB routine fmincon.m. To demonstrate the importance of accurate gradient calculation we compared results obtained using forward sensitivity equations and finite difference approximations. Forward sensitivities were computed using CVODES while finite differences were evaluated with a step-size of 10^−4^. The results are illustrated in Fig 3(b)–3(f).

Optimization using finite differences does not work reliably for the three considered descriptions. This can be attributed to poor accuracy in gradient computations. In regions where the objective function gradient entries are small, for instance close to the optimum, approximation errors caused by numerical integration of the ODE models can dominate over actual entries and thus lead to poor search directions. This can lead to premature termination of the optimization, if the objective function is locally ascending in the chosen search direction. The lowest objective function value achieved for finite differences is greater than the value obtained using forward sensitivities (Fig 3(a)). Moreover, no plateaus are observed [42]. This is the case for the RRE as well as 2MA and EMRE model. Using forward sensitivity equations we observed reproducible optimization results, substantiating that the global optimum is found. In addition to the superior convergence rate, the median computation times for one local optimization were consistently more than 10-fold faster using forward sensitivity analysis compared to finite differences (Fig 3(e)). This finding supports previous findings for ODE models [42] and underlines the importance of employing forward sensitivities as an efficient and robust gradient computation scheme.

A comparison across models revealed that the fitting results for RRE and 2MA are visually indistinguishable, while the EMRE differ slightly from both (Fig 3(b)). Furthermore, optimization of the RRE was indeed computationally most efficient (Fig 3(e)). The computation times for EMRE and 2MA were however only slightly higher. Interestingly, the minimal objective function value was more frequently reached for the EMRE and MA compared to RRE (Fig 3(d)). This indicates a larger region of attraction, reducing the number of required multi-starts and the convergence of alternative global optimization methods. Our results verify the practical feasibility of parameter inference using mesoscopic descriptions and potentially simpler objective function landscape.

#### Mesoscopic descriptions improve data exploitation

Optimization yielded the maximum likelihood estimates for the parameters of the biochemical process. Due to limited and noise corrupted data, these maximum likelihood estimates are often unreliable. We evaluated the uncertainty of the parameters obtained using RRE, EMRE and 2MA via profile likelihood calculation and Markov chain Monte-Carlo sampling. Profile likelihoods and marginal densities are provided in Figure B in S1 Supporting Information.

Profiles and marginals indicate identifiability of the four kinetic parameters *p*_1_-*p*_4_. Confidence intervals for these parameters are finite and agree for RRE, EMRE and 2MA. The initial STAT concentration, [STAT]_0_, has been shown to be structurally non-identifiable when using RRE [53]. This implied that independent of the amount of measurement data, the initial STAT concentration cannot be inferred using the RRE. Accordingly, the RRE yielded flat profiles for the initial STAT concentration. This was different for EMRE and 2MA. For EMRE, the lower bound of the 99% confidence and credibility intervals computed using profiles and marginals is 8 ⋅ 10^−3^ nM for the initial STAT concentration. For 2MA, we found lower bounds of 2 ⋅ 10^−2^ nM and 1 ⋅ 10^−1^ nM using profiles and marginals, respectively. This lower bound could only be derived as the reaction propensities are nonlinear and the reaction volumes as well as molecule numbers are finite. In this case the dynamics of the population mean are affected by fluctuations, which are controlled by initial concentrations. This dependency established structural identifiability and enabled us to exploit features of the data that could not be used by the RRE.

This finding is in line with results reported in the literature, which suggested that stochasticity can be exploited to improve the identifiability of parameters [18, 40, 72, 73]. Yet, previous analysis relied on using the process mean and variance for inference. The latter is only available for single-cell measurements. We demonstrated that stochasticity can be exploited even if only the process mean is available for inference. This renders stochastic inference attractive even if single-cell data is not available.

#### Literature validates lower bound for previously structurally non-identifiable parameter

To verify the lower bound for the initial STAT concentration derived using EMRE and 2MA, we screened additional literature. We found that Bachmann et al. [71] determined a STAT concentration of 80 nM under similar experimental conditions. This value is within the confidence/credibility bounds for both, EMRE and 2MA. While Bachman et al. [71] considered a different cell types, their results provide a partial confirmation of our finding.

In summary, the study of the JAK/STAT signaling pathway using EMRE and 2MA demonstrated the applicability of mesoscopic descriptions to real-world data. Using multi-start local optimization with accurate gradients, model parameters can be inferred from experimental data. Frequentist and Bayesian uncertainty analysis revealed that MA and SSE can provide additional insights, even if merely population-average data are available.

### Application to artificial data: Trimerization and enzymatic degradation

To assess the properties and potential of inference using mesoscopic descriptions (MA and SSE) in more detail, we study two processes: trimerization and enzymatic degradation. The use of artificial data enabled us to: (i) assess the estimation error introduced by macroscopic and mesoscopic descriptions; (ii) deduce a rule-of-thumb for the *a priori* selection of modeling approaches; and (iii) develop methods for the *a posteriori* selection and verification of modeling approaches.

#### Model description and artificial data generation

**Fig 4:**
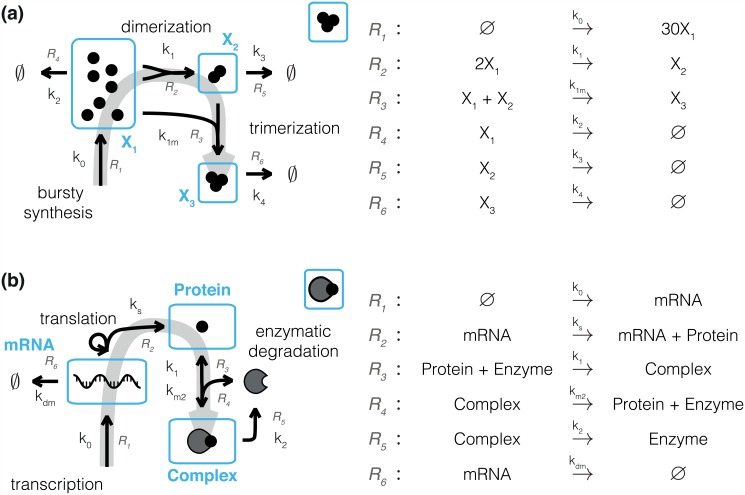
Reaction networks for comprehensive *in silico* evaluation of mesoscopic and macroscopic approaches. (a) Schematic of the trimerization process. (b) Schematic of the enzymatic degradation process. Arrows indicate reactions with the corresponding rate and reaction index next to them. Observed states are outlined and labeled in blue. A gray arrow represents the direction of information flow.

In the remainder, we study the trimerization process and the enzymatic degradation process depicted in Fig 4(a) and 4(b). The icons for the models introduced in Fig 4(a) and 4(b) will be used in the following figures to indicate the model in the respective study.

The trimerization process describes the bursty synthesis of monomers and their subsequent dimerization and trimerization [44]. Relevant biological applications of this model include receptor clustering and heat-shock factor trimerization [74, 75]. The trimerization process consists of 6 reactions and possesses 7 parameters (6 kinetic parameters and the reaction volume). Two reactions are bimolecular and hence have nonlinear propensities. Monomer, dimer and trimer concentrations are assumed to be measurable.

The enzymatic degradation process is an extension of the well-known two-stage model of gene expression [39, 76] and it has previously been studied in [77]. The enzymatic degradation process describes transcription and translation as well as enzymatic degradation of the gene product. It comprises several models of gene expression as special cases, e.g. [78–80]. The process consists of 6 reactions and possesses 8 parameters (6 kinetic parameters, the initial concentration of the enzyme and the reaction volume). The reaction resulting in the formation of the protein-enzyme complex is bimolecular and hence its propensity is nonlinear. The measured outputs are the mRNA, protein and complex concentrations.

A detailed mathematical description of trimerization and enzymatic degradation process is provided in S1 Supporting Information, Section 1.2.

For trimerization and enzymatic degradation process artificial data are generated using the SSA [21] with the parameter values in (Table B,D in S1 Supporting Information). A range of volumes Ω is considered to facilitate a comprehensive analysis of stochastic effects on estimation accuracy and to assess the regimes of validity for the different approximations. We considered realistic sample sizes in the range of *N*_*k*_ = 10^1^−10^4^, which are accessible by recent single-cell technologies [81]. The results of the parameter inference of the trimerization and the enzymatic degradation process, which are depicted schematically in Fig 4, are presented in the following.

#### Approximate descriptions result in estimation errors

**Fig 5:**
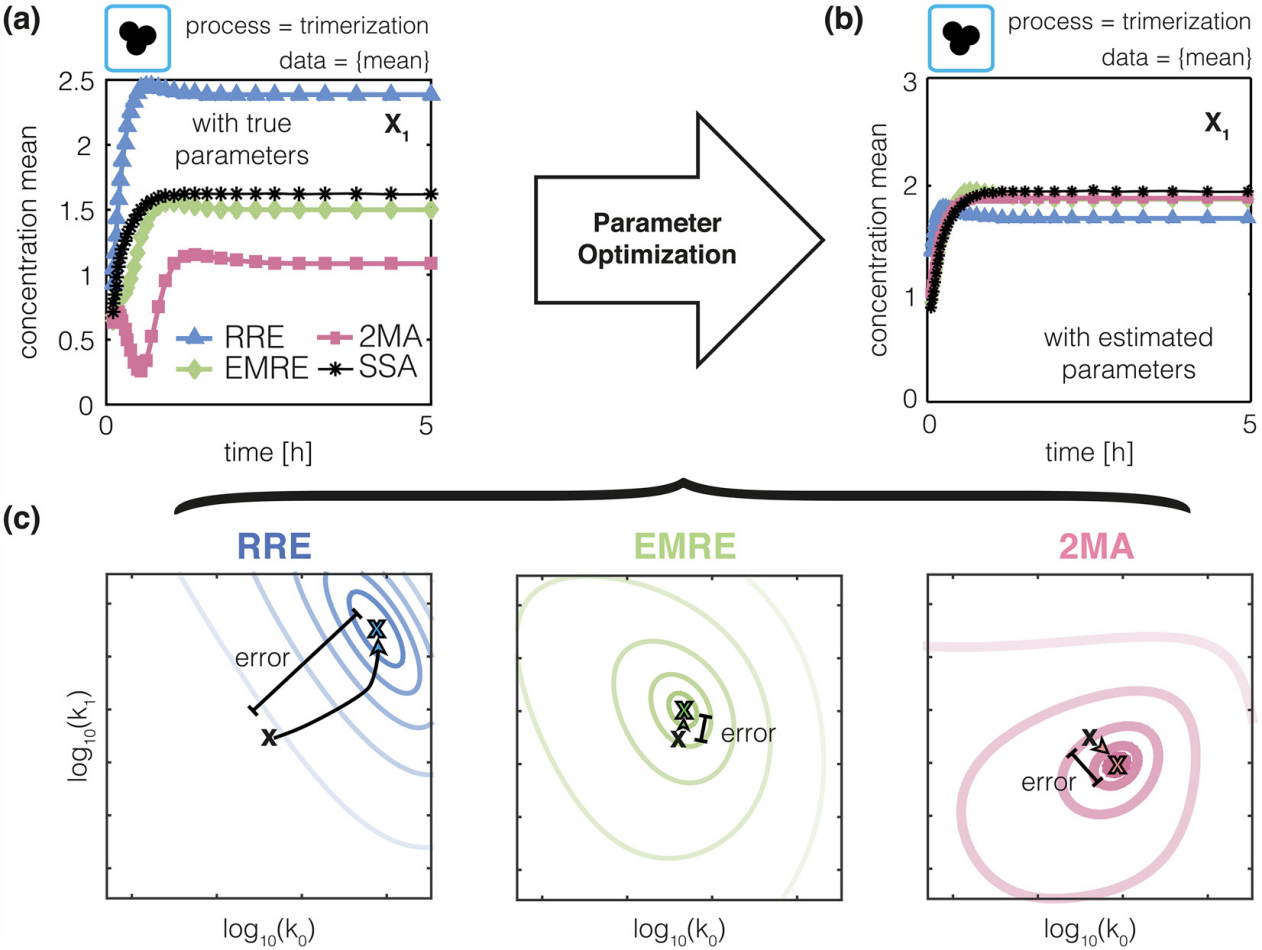
Approximation error introduces estimation error. (a) Mean monomer concentration in the trimerization process for Ω = 6*μ*m^3^ computed from 10^5^ SSA trajectory realizations (black line). Approximate mean monomer concentrations obtained using RRE, EMRE and 2MA (colored lines). (b) Mean monomer concentration for RRE, EMRE and 2MA obtained after parameter estimation using the SSA mean as artificial dataset. (c) True (black ×) and optimized parameter values (colored ×) for RRE, EMRE and 2MA. Contour lines of objective function are colored. The opacity increases with increasing likelihood values.

Macroscopic and mesoscopic descriptions provide only approximate estimates of the statistical moments of microscopic processes. These approximation errors may result in erroneous parameter estimates. This happens, for instance, when the approximation error can be partially or completely compensated by changing the parameter values, as we have illustrated in Fig 5 for the trimerization process. For small volumes, we find pronounced differences between the mean of the stochastic process determined using SSA and the means predicted by the RRE, EMRE and 2MA (Fig 5(a)). We regarded the mean of the SSA runs as artificial data and optimized parameters of RRE, EMRE and 2MA using the aforementioned multi-start local optimization with accurate gradients. The optimized trajectories for RRE, EMRE and 2MA agree well with the mean of the SSA runs as shown in Fig 5(b). This agreement is achieved for parameter values deviating from the true parameter values used for the stochastic simulation. The objective function landscapes of the individual models shown in Fig 5(c) indicate that the optimum of the objective function does generally not coincide with the true parameters. This pattern is reproducible and is caused by the error of the approximation methods resulting in erroneous, biased parameter estimates.

#### Mesoscopic descriptions improve the estimation accuracy at intermediate volumes

As the estimation error is caused by the approximation error of the statistical moments on which the inference is based, a relation between the magnitude of the approximation error and the estimation error is to be expected. Since mesoscopic descriptions (EMRE,2MA) tend to have smaller approximation errors than macroscopic descriptions (RRE) [23, 29, 36], the former are expected to lead to smaller estimation errors as we have have demonstrated in Fig 5(c). We will now give a verification of these arguments. To assess the estimation error we generated 100 artificial datasets, each containing 10^5^ cells for different volumes, and evaluated the estimation accuracy of the parameter estimation. The workflow is illustrated in Figure C in S1 Supporting Information. For the inference we used MA and the SSE truncated to various orders:

- data = {mean} → inference using RRE, EMRE and 2MA.
- data = {mean,variance} → inference using LNA, IOS and 3MA.

**Fig 6:**
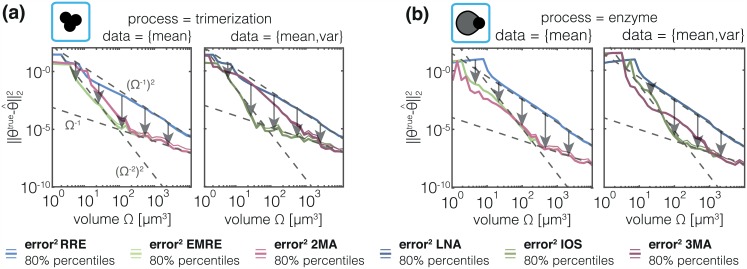
Quantification of volume dependence of estimation error. Medians (thick line) and symmetric 80% percentile based confidence intervals (thin lines) of the errors for two representative parameters of (a) the trimerization process and (b) enzymatic degradation process. Results for different meso- and macroscopic models are color-coded and panels show datasets computed from 10^5^ single-cell measurements: (left) data = {mean}; and (right) data = {mean,variance}. The estimated convergence order for the intermediate and high-volume regimes is indicated as gray dotted lines.

Medians and 80% symmetric percentile intervals of the squared estimation error,

$${\text{error}}^{2}={\left\|\theta^{\text{true}}-\hat{\theta }\right\|}_{2}^{2},$$

were calculated and the results are shown in Fig 6 for both processes.

In accordance with our hypothesis, we found that mesoscopic descriptions using higher-order SSEs and MAs tend to yield a lower estimation error compared to macroscopic descriptions, here RRE and LNA. The difference between meso- and macroscopic descriptions is most pronounced for intermediate volumes (10^1^ *μ*m^3^ − 10^3^ *μ*m^3^). As expected, for large volumes—where micro-, meso- and macroscopic descriptions agree—all descriptions resulted in small estimation errors. For small volumes, meso- and macroscopic descriptions depart from the underlying process resulting in large estimation errors which render results meaningless. For the enzymatic degradation process, higher-order MAs and SSEs sometimes yield higher estimation errors than low-order MAs and SSEs. This might come surprising, but the approximation order is only informative about the approximation error in the large volume limit and does not allow conclusions for low volume regimes. Accordingly, the superiority of higher-order approximations cannot be expected in low volume regimes.

In the medium- to high-volume regime we would expect an approximation order of Ω^−1^ for RNA/LNA and Ω^−2^ for EMRE/IOS. Accordingly, in the absence of measurement noise, the convergence order of the mean squared error should be (Ω^−1^)^2^ and (Ω^−2^)^2^ respectively. In fact, the observed convergence rates agree with these theoretical rates which are indicated by dashed gray lines in Fig 6. In the medium- to high-volume regime, the convergence rates are dominated by the bias fraction of the mean squared error. However, for high volume regimes we observe a convergence rate of approximately Ω^−1^ for EMRE/IOS. In this regime, the convergence rate of the mean squared error is dominated by the variance of the parameter estimator. Thus the convergence rate can be expected to be proportional to the variances of sample means and variances $\sigma_{{\hat{\mu }}_{i,k}}^{2}$ and $\sigma_{{\hat{\Sigma }}_{ii,k}}^{2}$. For the considered setting the convergence rate seems to be dominated by $\sigma_{{\hat{\mu }}_{i,k}}^{2}=\frac{1}{N}\Sigma_{ii}$, which scales, according to the LNA, as $\frac{1}{N}\Omega^{-1}$. We expect that for higher volumes, the convergence rate of RRE/LNA will also be limited by the estimation variance and thus attune to $\frac{1}{N}\Omega^{-1}$. The decomposition of the mean squared error for the two models is provided in Figure E in S1 Supporting Information. Furthermore, this theoretical limit suggest that an increase in the number of measured cells *N* should result in a shift of this variance limit to lower values.

For the simulation examples, including variance information did not yield any consistent reduction of the estimation error. This might come as a surprise as a previous study suggested that the variance carries considerable amounts of information which when included can even render previously non-identifiable parameters identifiable [39]. However, for the simulation examples we considered a data-rich setting where all parameters are well identifiable and the estimation error is mainly due to the approximation error of the description. In less data-rich situations and in the presence of technical noise, we expect that including variance information could also reduce the estimation error.

SSE and MA methods achieved similar estimation accuracies for the trimerization and enzymatic degradation processes. However, optimization using SSE turned out to be computationally more efficient than MA, as robust numerical integration of the respective differential equations was less problematic, see Figure D in S1 Supporting Information. In the following we present the results for RRE, LNA, EMRE and IOS while the results for 2MA and 3MA are reported in Figure F-I in S1 Supporting Information.

#### Mesoscopic descriptions are beneficial for the analysis of high-throughput single-cell data

**Fig 7:**
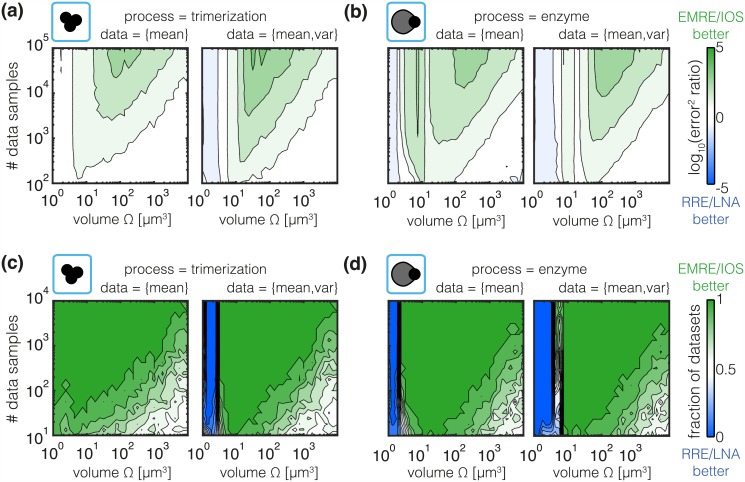
Quantification of sample size dependence of estimation error. (a,b) Ratio of the absolute estimation errors. Green indicates a lower estimation error for EMRE and IOS while blue indicates a lower estimation error for RRE and LNA. (c,d) Frequency for lower estimation error for EMRE and IOS compared to RRE and LNA. The color indicates the fraction of datasets for which EMRE and IOS yields a lower estimation error than RRE and LNA.

As we have seen in the previous section, the sample size influences the estimation of mean and variance, we studied its impact on the accuracy of inference with different models. We determined the estimation errors for RRE, LNA, EMRE and IOS using 100 artificial data sets of different sample sizes and volumes. This detailed analysis confirmed that RRE and LNA generally yield larger estimation errors than EMRE and IOS. Interestingly, the regime of volumes for which this is consistently observed increases with sample size as we show in Fig 7(a) and 7(b, green area). In Fig 7(c) and 7(d) we verify that this relation holds not only on average but also for individual datasets resulting in lower estimation errors. As expected, this is the case for intermediate to large volumes. Only for small volumes, the approximation was unsatisfactory and RRE and LNA were occasionally favored over EMRE and IOS methods.

Depending on the experimental devices, the number of single-cell recordings ranges from tens to hundreds of thousands measured cells. High-content single-cell methods, such as single-cell RNAseq and single-cell time-lapse microscopy, are mostly used to study tens to hundreds of cells [82, 83]. High-throughput single-cell methods, flow and mass cytometry, enable the assessment of thousands of cells but provide merely a smaller number of features [84]. Intuitively, the high-throughput single-cell methods reduce the sampling error as many cells are recorded and can therefore be well characterized by moments. Hence higher-order SSEs are particularly valuable for the analysis of high-throughput single-cell data. This simple rule-of-thumb for the *a priori* selection of the modeling approach is also corroborated by our findings for MA Figure F,G in S1 Supporting Information.

#### Model selection pinpoints regimes in which mesoscopic descriptions yield improved approximation accuracy

Our results suggest that meso- and macroscopic descriptions are only appropriate for inference in certain volume and sample size regimes. In practice, the boundaries of these regimes remain unknown. To identify the most appropriate description in a certain regime *a posteriori*, we propose the use of model selection methods.

**Fig 8:**
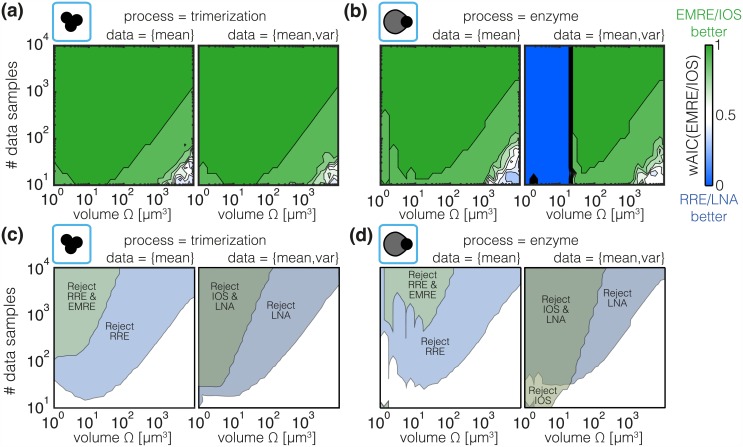
Analysis of model selection and rejection criteria. (a) and (b) Median AIC weight for EMRE and IOS at respective estimated parameters. A green color indicates that the EMRE and IOS description is more probable and a blue color indicates the RRE and LNA description is more probable. (c) and (d) area in which the models can on average be rejected based on a chi-square test to confidence level 0.01. The coloring indicates the method to which the area corresponds.

We employed AIC to select the most appropriate among a set of candidate models given by the macro- and mesoscopic descriptions of the processes. Fig 8 depicts the AIC weights—interpretable as posterior probabilities—of EMRE and IOS for different volumes and sample sizes. We find that EMRE and IOS are favored over RRE and LNA everywhere except in two regimes that provide additional insights:

- **Regime I** is classified by large volumes and low sample sizes. The AICs of RRE and EMRE as well as LNA and IOS are comparable—AIC weights close to 0.5—as the models fit the limited data fairly well. If the statistical power of the data is however increased by increasing the sample size, EMRE and IOS are favored as descriptions provided by RRE and LNA are no longer sufficiently accurate. This indicates that the statistical power is simply not sufficient to reveal the small differences in between EMRE/IOS and RRE/LNA.
- **Regime II** appears only for the inference of the enzymatic degradation model using data for mean and variance. For volumes below 10 *μ*m^3^, LNA is favored over IOS. The reason for this is that the LNA leads to a physically meaningful description, i.e. positive variances for all volumes, whereas the IOS leads to positive variances (which correct the LNA) for large enough volumes but can potentially give rise to negative variances for small enough volumes. The latter is possible since terms in the SSE beyond the LNA, i.e., those involving third- and higher-order derivatives, do not lead to a Fokker-Planck description which can imply negative values of the approximated probability density function [37, 85]). Hence the LNA becomes favorable over the IOS for small volumes.

Accordingly, model selection favors the macroscopic description over the mesoscopic one either when the statistical power is too limited to reject them (Regime I) or when they are indeed more accurate (Regime II). Otherwise, mesoscopic descriptions based on higher-order SSEs or MAs (Figure H,I in S1 Supporting Information) are selected.

The selection also resembles the results for the estimation error in Fig 7(a) and 7(b). The critical volume for which the AIC weights depart from unity coincides with the upper bound of the intermediate regime in which mesoscopic description provide lower estimation errors. Furthermore, in Regime II IOS yields large estimation errors. In summary, this suggests that model selection can be used (i) to decide whether a mesoscopic or a macroscopic description is appropriate and (ii) to improve the quality of parameter estimates.

#### Model rejection criteria can reveal the necessity of a microscopic description

The superiority of a model according to model selection criteria does not imply that the favored model accurately represents the data. Specific applications may indeed require microscopic descriptions to model experimental data. To check this, simulation and parameter estimation using microscopic descriptions could be performed. While efficient algorithms have been developed, such procedure is often time-consuming. We therefore considered model rejection to assess the necessity of microscopic modeling without performing the microscopic analysis.

We computed the goodness-of-fit and employed a *χ*^2^-test with a confidence level of 0.01 for model rejection. Fig 8(c) and 8(d) illustrates the regimes in which the meso-/macroscopic descriptions have been rejected for at least 50% of the artificial datasets. We find that regions in which higher-order SSEs are rejected are mostly contained in regions for which lower-order SSEs are rejected.

As sample size increases higher-order SSEs and MAs are rejected for increasingly larger volumes. This is plausible as the improved statistical power allows us to resolve smaller differences between microscopic and the corresponding meso-/macroscopic descriptions. The statistical power is determined by the number of samples and the statistical moments of the samples. If the difference between approximative descriptions and the process is large, a small sample size is sufficient to rule out a model, while a large number of samples is required to detect smaller differences. As the difference between approximative descriptions and the process is volume-dependent and process-specific, the regions in which the approximative description can be rejected might possess a complex shape. For the enzymatic degradation model we find for instance that for low sample sizes the RRE can merely be rejected for an intermediate volume regime but not for small or large volumes. The dependence on the number of samples supports also the finding that for the analysis of high-throughput data accurate models need to be employed.

The proposed approach based on model rejection reveals the need for a more accurate description without performing the corresponding analysis. Accordingly, macroscopic models such as RRE and LNA can be used to perform the initial analysis. Only if these models are rejected using the *χ*^2^-test, mesoscopic descriptions need to be employed. In the same way also the necessity of microscopic descriptions can be assessed without actually performing the corresponding time-consuming analysis.

In summary, the study of trimerization and enzymatic degradation model clearly revealed that higher-order SSEs and MAs are generally more reliable. The increased computational complexity is tractable and the investment becomes worthwhile for high-throughput data in particular. Further improvement could be achieved by combining model selection criteria and rejection criteria.

## Discussion

Many biological processes exhibit stochastic fluctuations which are relevant for cells and organisms [1–3]. Quantitative mechanistic models facilitate an understanding of the relevance of these fluctuations to dynamics over various length scales. Despite significant progress, the parameterization of such quantitative mechanistic models remains challenging. In this work, we implemented sophisticated parameter estimation and uncertainty analysis methods relying on mesoscopic descriptions of stochastic processes, namely higher-order SSEs and MAs.

We verified the developed methods using simulation examples. We found that for intermediate and large volumes, for which inference using microscopic descriptions is computationally already demanding, our approximate methods provides reliable estimation results. The computation time required for optimization was a fraction of the computation time required for the stochastic simulation of the stochastic process (c.f. Figure A in S1 Supporting Information). Compared to estimation methods using macroscopic descriptions, such as RRE or LNA [40], a significantly decreased estimation error is observed for intermediate volumes. This intermediate regime increases with the number of single-cell measurements. Our parameter estimation methods using higher-order SSEs and MAs are therefore especially suited for the data-driven modeling of high-throughout data, such as flow and mass cytometry data.

As the unnecessary study of meso-/microscopic descriptions can be time-consuming, we also considered model rejection approaches. We found that the application of such methods can guide model refinement. The methods cannot however distinguish between inappropriateness arising from meso- and microscopic descriptions due to an inaccurate knowledge of the biochemical reaction network as both result in a disagreement of model and data. In addition, for applications with multiple candidate models it is not guaranteed that model selection results obtained for macroscopic descriptions will be reproduced for the corresponding mesoscopic or microscopic descriptions. Thus model selection and model rejection methods should always be combined. If the microscopic description of all candidate models were rejected using the *χ*^2^-test, the set of candidates would not contain a model which accurately represents the data and should be extended.

Beyond the study of artificial data, we employed the proposed methods to study experimental data for the JAK/STAT signaling pathway. This revealed that mesoscopic modeling can also provide additional insights if merely population-average data are available. For processes with non-linear reaction propensities, the mean encodes information about the volume and the molecular numbers, respectively [29, 36]. This enabled the estimation of a lower bound for the initial STAT concentration, a parameter, which is structurally non-identifiable when macroscopic descriptions are employed. To assess the lower bound we implemented profile likelihood calculations and MCMC methods for higher-order SSEs and MAs. MCMC methods for MAs had already been proposed [10], the combination of Bayesian and frequentist methodology is however known to provide more robust results [55, 86]. The derived lower bound for the STAT concentration could be confirmed with literature data. The insight could be obtained for a well-studied system and pinpoints the great potential of mesoscopic descriptions for data-driven modeling.

However, the use of mesoscopic descriptions also has certain drawbacks. It is for instance not completely clear how practical and structural identifiability of the stochastic process (described by the CME) and the approximative descriptions are related [18]. Furthermore, as higher-order SSEs and MAs are merely approximations to data generating processes, the resulting estimators are inconsistent. Hence, parameter estimates and confidence intervals can be erroneous. In principle, this problem can be addressed using ideas developed in the fields of model reduction [87, 88] or probabilistic numerical simulations [89, 90]. These methods require upper bounds for the approximation error or the error distribution of vector field approximation, respectively. Approximations for both might be obtained by using a sequence of higher-order expansions. A rigorous treatment would yet require exact bounds, as available for the FSP [22].

In this study we employed higher-order SSEs and MAs to approximate the moments of the stochastic process for inference. A further improvement could be achieved by using hybrid approaches, such as the method of conditional moments [91] or the conditional system size expansion [92]. These approaches exploit a microscopic description of low-copy number species and a mesoscopic description for medium- to high-copy number species. Complementarily, higher-order SSEs and MAs could be used to enhance the accuracy of ODE constrained mixture modeling [93]. This modeling and analysis method accounts for the subpopulation structure but relies on simple macroscopic descriptions for the subpopulation dynamics. The use of macroscopic descriptions could result in a reduction of the number of parameters and an improved data exploitation.

Until now, the stochasticity of biological systems is often disregarded as its analysis is computationally demanding. The emergence of measurement techniques such as single-cell fluorescent microscopy [83, 94], flow and mass cytometry [84], single-cell qPCR [95] and single-cell RNA-seq [82] renders the consideration of stochastic effects a necessity [96, 97]. The presented methods are computationally efficient and scalable. This will facilitate the quantitative mechanistic modeling of complex cellular process es and the exploitation of cell-to-cell variability for biological discovery.

## Supporting Information

S1 Supporting Information Supplementary notes regarding modeling and computational analysis. This document provides a detailed description of the biochemical reaction networks and their parameters, system size expansion and moment approximation, as well as the parameter estimation and the uncertainty analysis results.
(PDF)

S1 Code MATLAB code used for inference using SSE and MA. This zip-file contains the MATLAB code for the simulation and application example presented in the paper. We provide implementations of all models, parameter estimation and uncertainty analysis to allow everybody to reproduce the results.
(ZIP)
