# Supplementary material for: Inference for Stochastic Chemical Kinetics Using Moment Equations and System Size Expansion
Source: PLoS Comput Biol. 2016 Jul 22;12(7):e1005030. doi: 10.1371/journal.pcbi.1005030 (PMC4957800; doi:10.1371/journal.pcbi.1005030)
Supplement: S1 Supporting Information — This document provides a detailed description of the biochemical reaction networks and their parameters, system size expansion and moment approximation, as well as the parameter estimation and the uncertainty analysis results. (PDF) [file pcbi.1005030.s001.pdf]

Supplement to
Inference for stochastic chemical kinetics using moment equations and system size expansion

Fabian Fröhlich [1,2], Philipp Thomas[3], Atefeh Kazeroonian[1,2], Fabian J. Theis[1,2], Ramon Grima[4], Jan Hasenauer[1,2]

1 Helmholtz Zentrum München - German Research Center for Environmental Health, Institute of Computational Biology, Ingolstädter Landstr. 1, 85764 Neuherberg, Germany

2 Technische Universität München, Center for Mathematics, Chair of Mathematical Modeling of Biological Systems, Boltzmannstr. 3, 85748 Garching, Germany

3 Department of Mathematics, Imperial College London, London SW7 2AZ, United Kingdom

4 School of Biological Sciences, University of Edinburgh, Edinburgh EH9 3BF, United Kingdom

Contents

1 Supporting Information S1 **1**
1.1 Numerical simulation 1
1.2 Model Definitions 2

2 Parameter Estimation for the Simulation Examples **4**
2.1 Further Quantification of Estimation Error and Model Selection Criteria 6

1 Supporting Information S1

1.1 Numerical simulation

MA and SSE yield ordinary differential equation models. For these models the forward sensitivity equations are derived. These equations describe the derivative of the time-dependent state of the ODE with respect to the parameter. To solve the ODE system for states and forward sensitivities simultaneously the SUNDIALS library CVODES [1, 2] is used. CVODES allows for highly efficient numerical integration and is usually significantly faster than the corresponding MATLAB implementation. For the simulation we used the BDF integrator with a Newton dense non-linear solver. For relative and absolute tolerances we used 10^{-8} for the state equations and estimated tolerances for the sensitivity equations via the `CVodeSensEEtolerances` command. To prevent numerical problems for systems that exhibit weakly damped oscillations, we activated stability limit detection via the `CVodeSetStabLimDet` command.

We compared the resulting simulation time to those of the SSA method used for data generation. The results are shown in Figure 1. Even for the considered small-scale examples, the simulation of IOS and 3MA is on average faster than computing an emsemble of 100 SSA runs. Typically thousands of SSA runs are necessary to obtain reliable statistics.

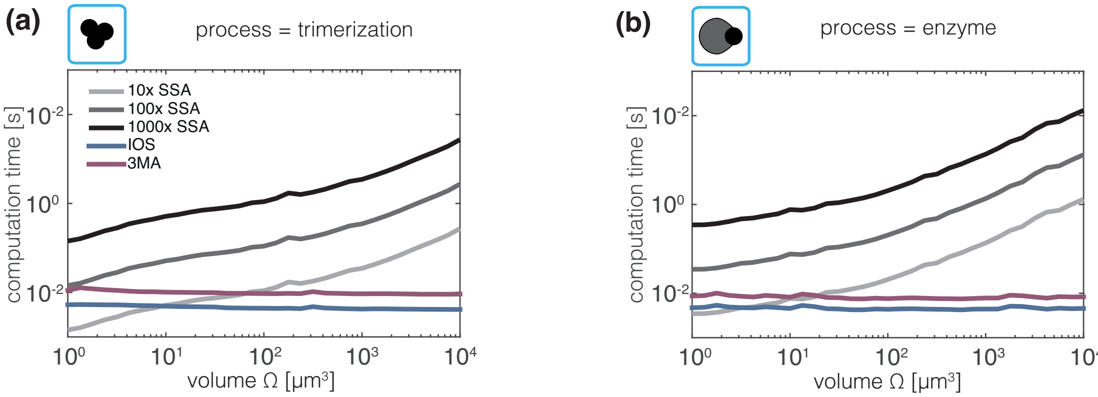

Figure 1. Comparison of the computation time of SSA and 3MA and IOS approximations for the trimerization and enzymatic degradation model. For SSA, the computation time for ensembles of size 10, 100 and 1000 are shown. For IOS and 3MA the average computation time across 1000 simulations is shown. All simulations were carried out at the true parameter which were also used for data generation.

1.2 Model Definitions

In the following we specify the reactions in the trimerization model, the model of enzymatic protein degradation and the JAK/STAT signaling pathway. We do not deem a definition of the individual equations reasonable, as the systems of equations become quite large for higher order expansions. This renders a manual implementation of the equations error-prone and an automatic generation of differential equations via e.g. the ACME toolbox much more tractable. Where applicable the models were reduced by their conservation laws.

Trimerization Model The trimerization model was formulated using mass-action kinetics leading to the reactions provided in Table 1 with the parameters provided in Table 2. The three species were initialized with zero molecules at time $t = 0$.

Enzymatic Degradation Model The reactions of the reduced system are given in Table 2 while parameters are provided in Table 4. The model was then reduced by the enzyme-protein complex exploiting the conservation law

$$[\text{Complex}] = [\text{Enzyme}]_0 - [\text{Enzyme}],$$

where we assumed that all species have zero molecules initially except the enzyme species whose initial concentration is $[\text{Enzyme}]_0$.

JAK/STAT model The JAK/STAT model consists of nine species of nuclear and cytosol compartments. The respective compartment volumes of BaF3 cells are $\Omega_{\text{nuc}} = 450\,\mu\text{m}^3$ and $\Omega_{\text{cyt}} = 1400\,\mu\text{m}^3$ [3]. We assume that initially only $STAT$ is present in the cytosol at a concentration of $[\text{STAT}]_0$. The reactions in this pathway are given in Table 5. Note that the overall concentration of $STAT$ is constant and hence the model can be reduced by the concentration of the nuclear complex which is given by

$$\begin{aligned}2[\text{npSTAT:npSTAT}] = \frac{\Omega_{\text{cyt}}}{\Omega_{\text{nuc}}}\big([\text{STAT}]_0 - [\text{pSTAT}] - 2[\text{pSTAT:pSTAT}]\big) \\ - \big([\text{nSTAT1}] + [\text{nSTAT2}] + [\text{nSTAT3}] + [\text{nSTAT4}] + [\text{nSTAT5}]\big).\end{aligned}$$

Note that $pEpoR(t)$ in Table 5 denotes a time-dependent function describing phosphorylation of STAT which is parametrized by a cubic spline between the time points 0, 5, 10, 20 and 60. Its five parameters are estimated along with scaling parameters and the biologically relevant parameters p_1, p_2,p_3, p_4 and $[\text{STAT}]_0$.

Since the SSE is commonly formulated for a single compartment, we performed the multi-compartment analysis by rescaling the bimolecular reaction rate constant p_2 by the cytosolic volume and performing the

Table 1. Reactions of the trimerization model.

index	educts		product	rate constant
R_1	$\varnothing$	$\rightarrow$	$30X_1$	k_0
R_2	$2X_1$	$\rightarrow$	X_2	k_1
R_3	$X_1 + X_2$	$\rightarrow$	X_3	k_{1m}
R_4	X_1	$\rightarrow$	$\varnothing$	k_2
R_5	X_2	$\rightarrow$	$\varnothing$	k_3
R_6	X_3	$\rightarrow$	$\varnothing$	k_4

Table 2. Parameter values of the trimerization model from which reference dataset was obtained.

Parameters	value	unit	lower bound	upper bound
k_0	$\frac{1}{3}$	$1/(\mu m^3 \cdot h)$	10^{-2}	10^4
k_1	0.5	$\mu m^3/h$	10^{-2}	10^4
k_{1m}	1	$\mu m^3/h$	10^{-2}	10^4
k_2	1	1/h	10^{-2}	10^4
k_3	1	1/h	10^{-2}	10^4
k_4	1	1/h	10^{-2}	10^4

Table 3. Reactions of the enzymatic degradation model.

index	educts		product	rate constant
R_1	$\varnothing$	$\rightarrow$	mRNA	k_0
R_2	mRNA	$\rightarrow$	mRNA+Protein	k_s
R_3	Protein + Enzyme	$\rightarrow$	Complex	k_1
R_4	Complex	$\rightarrow$	Protein + Enzyme	k_{m2}
R_5	Complex	$\rightarrow$	Enzyme	k_2
R_6	mRNA	$\rightarrow$	$\varnothing$	k_{dm}

Table 4. Parameter values of the trimerization model from which reference dataset was obtained.

Parameters	value	unit	lower bound	upper bound
k_0	2.25	$1/(\mu m^3 \cdot h)$	10^{-4}	10^4
k_s	4	1/h	10^{-4}	10^4
k_1	100	$\mu m^3/h$	10^{-4}	10^4
k_{m2}	1	1/h	10^{-4}	10^4
k_2	1	1/h	10^{-4}	10^4
k_{dm}	10	1/h	10^{-4}	10^4
$[\text{Enzyme}]_0$	1	$1/\mu m^3$	10^{-4}	10^4

Table 5. Reactions of the JAK/STAT Model. The function $pEpoR(t)$ is described in the text.

index	educts		product	rate constant
R_1	STAT	$\rightarrow$	pSTAT	$p_1 \cdot \mathrm{pEpoR}(t)$
R_2	2 pSTAT	$\rightarrow$	pSTAT:pSTAT	$p_2/(\Omega_{\mathrm{cyt}}[\mathrm{STAT}]_0)$
R_3	pSTAT:pSTAT	$\rightarrow$	npSTAT:npSTAT	p_3
R_4	npSTAT:npSTAT	$\rightarrow$	2 nSTAT1	p_4
R_5	nSTAT1	$\rightarrow$	nSTAT2	p_4
R_6	nSTAT2	$\rightarrow$	nSTAT3	p_4
R_7	nSTAT3	$\rightarrow$	nSTAT4	p_4
R_8	nSTAT4	$\rightarrow$	nSTAT5	p_4
R_9	nSTAT5	$\rightarrow$	STAT	p_4

Table 6. Parameter estimates and minimal objective function values for the JAK/STAT model for different descriptions of the mean behavior.

parameter	estimate RRE	estimate EMRE	estimate 2MA	unit	lower bound	upper bound
p_1	3.82	5.79	4.01	1/min	10^{-5}	10^3
p_2	$9.58 \cdot 10^5$	$6 \cdot 10^6$	$3.98 \cdot 10^5$	1/min	10^{-3}	10^6
p_3	0.11	0.11	0.11	1/min	10^{-5}	10^3
p_4	0.98	0.95	0.86	1/min	10^{-5}	10^3
$[\mathrm{STAT}]_0$	$3.55 \cdot 10^5$	2.06	$1.28 \cdot 10^5$	nM	10^{-3}	10^6
$J_M(\hat{\theta})$	73.7261	74.9429	73.7200			

SSE for a unit volume. Effectively, this procedure yields expressions for the moments in units of molecules numbers. The resulting averages are then divided by the compartment volume of the respective species while the variances are divided by the volume squared to obtain the concentration moments. Similarly, the MA estimates were obtained. Equivalent formulations for the SSE and MA with multiple compartments have given in Refs. [4,5] and [6], respectively.

2 Parameter Estimation for the Simulation Examples

We carried out parameter estimation for all generated datasets. In the following we will outline the results found for one of the datasets for the trimerization model at $\Omega = 100\mu\mathrm{m}^3$. These results are representative for both models and other sample size and volume scenarios. Figure 4 (a) shows the simulation of the different description at the respective estimated parameters and the data which was used to estimate parameters for the trimerization model. We find that there is a good agreement between simulation and data. To demonstrate the efficiency of optimization using sensitivity (SE) based gradients over finite difference (FD) based gradients we compared convergence rate and computation time for both approaches (c.f. Figure 4 (b-c)). Both approaches yield comparable convergence rates to the lowest found objective function values across all descriptions. In contrast, we find pronounced differences in computation time. FD optimization takes almost 10 times longer than SE optimization. This means that SE optimization yield 10 times more converged starts than FD optimization in the same amount of time. Comparing the optimization time between different descriptions, we find that on average the required time is of the same order of magnitude for all descriptions. Hence for problems where RRE based parameter estimation is feasible, MA and SSE based parameter estimation should be feasible as well.

For the MA a large fraction of optimizer runs ended in parameter domains where numerical integration was not possible. Figure 4(b) therefore only includes runs where no such difficulties occurred. For the SSE all runs are shown, which indicates no problems with integrability. This means that for this systems MA will in general require more starts to obtain the same number of convergent runs. Similar problems were

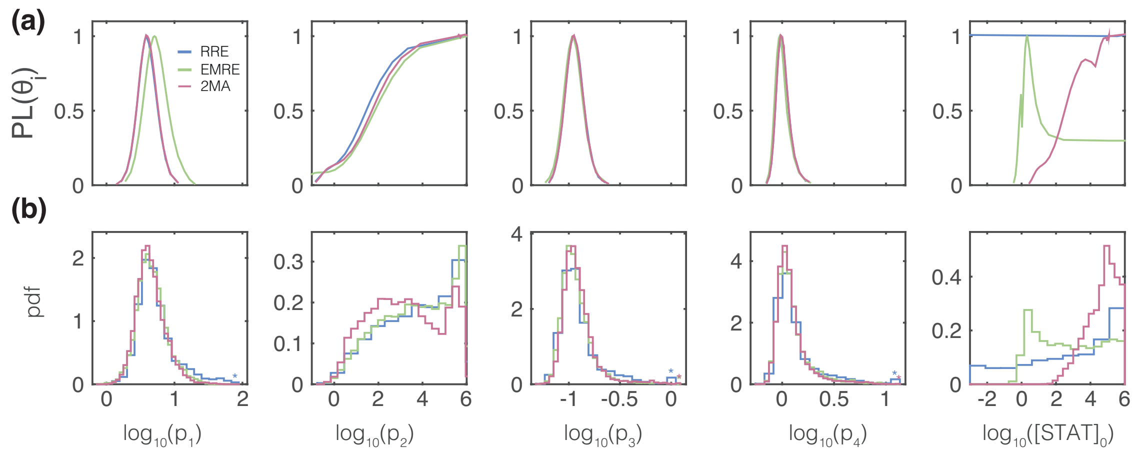

Figure 2. Comparison of uncertainties of parameter estimates for the JAK/STAT pathway. (a) Profile densities for the 5 biologically relevant parameters. (b) Histogram approximation of marginal densities.

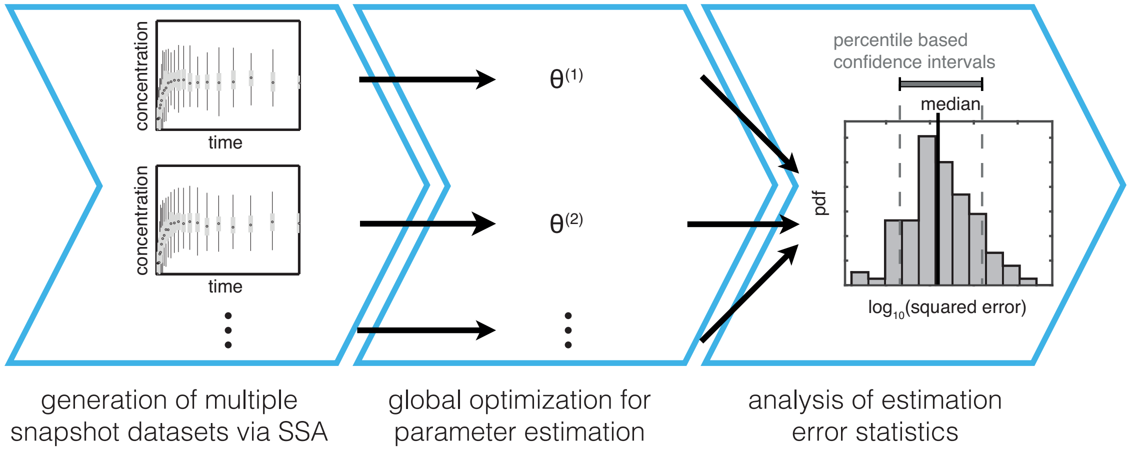

Figure 3. Workflow schematic for error analysis of the parameter estimation. The left panel shows two exemplary datasets generated via SSA. From these either mean or mean and variance is used to estimate parameters for the different models, which is represented symbolically in the middle panel. Parameters are optimized using a local gradient based optimizer in a multi-start optimization resulting in a global optimization scheme. The estimated parameters are subsequently compared to the true parameters via the euclidean distance. Exemplary statistics of this squared error across all datasets are shown in the right panel.

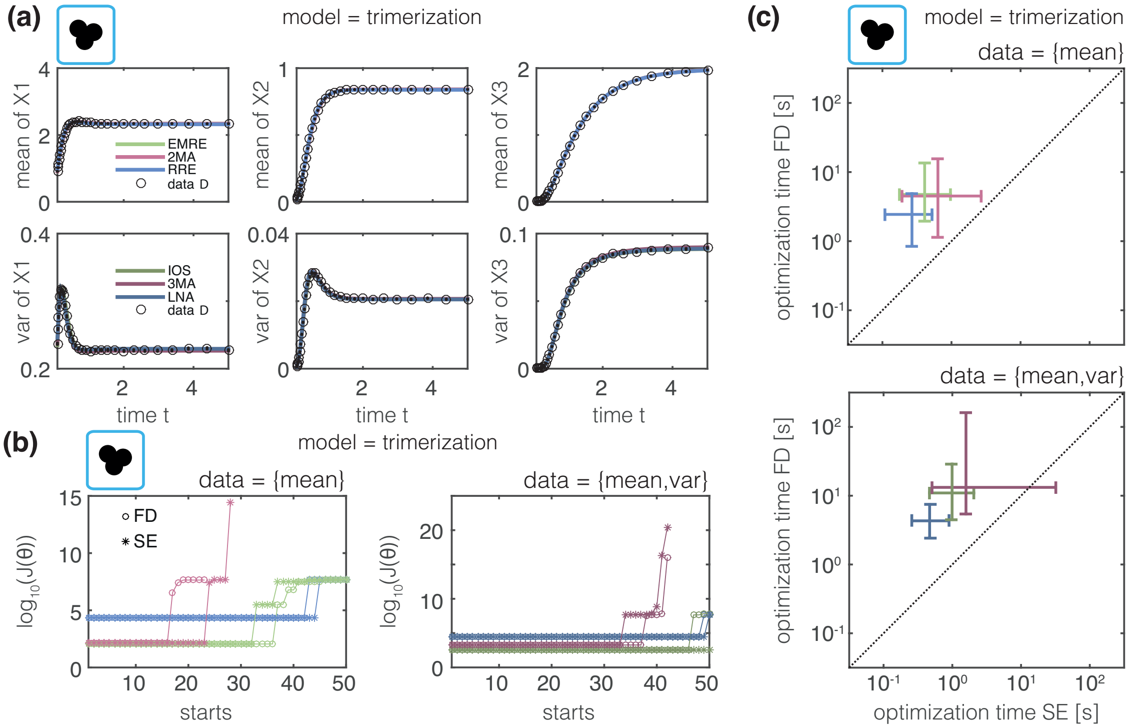

Figure 4. Parameter estimation for the trimerization model. (a) Objective function values for different initializations using finite difference (FD, o) and sensitivity based gradients (SE, *). Function values are colored according to the employed description. (b) Comparison of optimization times for finite differences and sensitivity based gradients for a single start. Crosses indicate 90% percentiles of optimization times across finished starts and are centered around mean optimization times. Crosses are colored according to the employed description.

encountered for the MA of the enzymatic degradation model as well.

2.1 Further Quantification of Estimation Error and Model Selection Criteria

In the following we provide a comparison of the estimation error and uncertainty analysis for all model parameters between RRE and EMRE as well as LNA and IOS. Moreover we provide a comparison of estimation errors and model selection between EMRE and 2MA as well as IOS and 3MA.

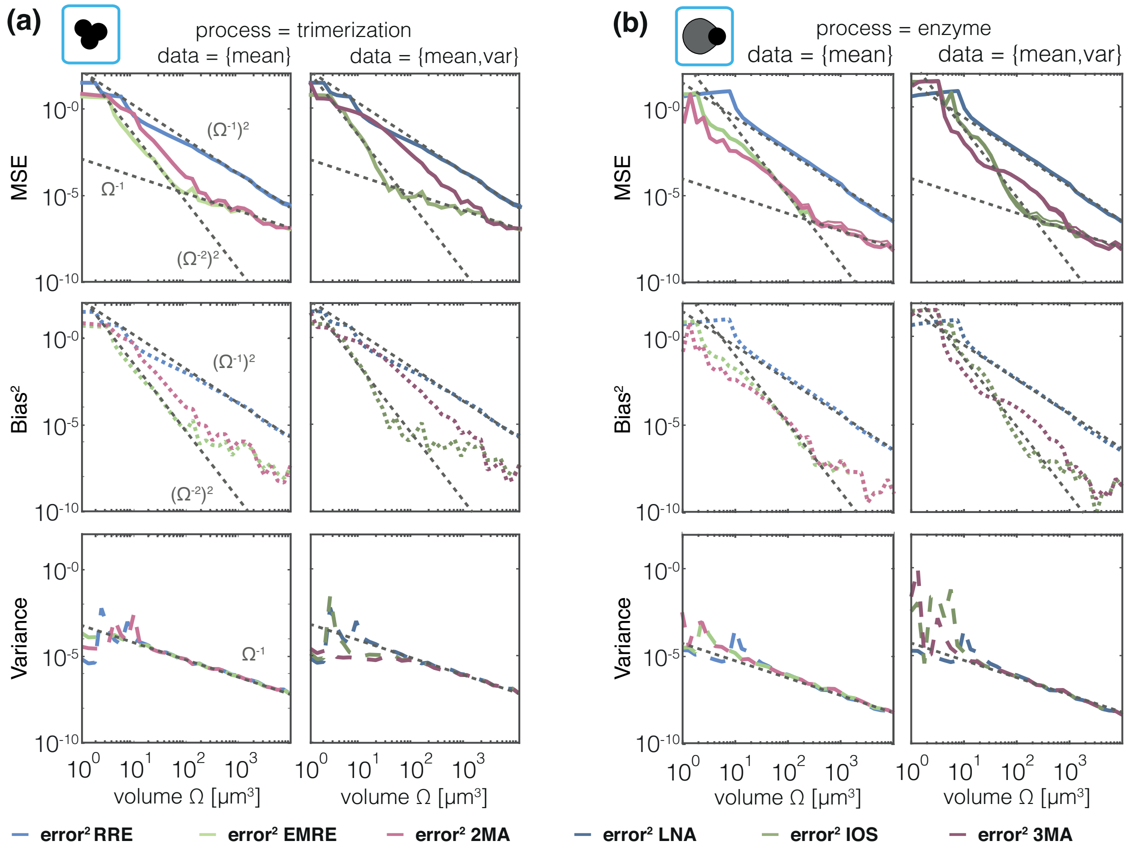

Figure 5. Decomposition of volume dependence of estimation error. Medians squared, squared bias and trace of variance of the estimator for two representative parameters of (a) the trimerization process and (b) enzymatic degradation process. Results for different meso- and macroscopic models are color-coded and panels show datasets computed from 10^5 single-cell measurements: (left) `data = {mean}`; and (right) `data = {mean,variance}`. The estimated convergence order for the intermediate and high-volume regimes is indicated as grey dotted lines.

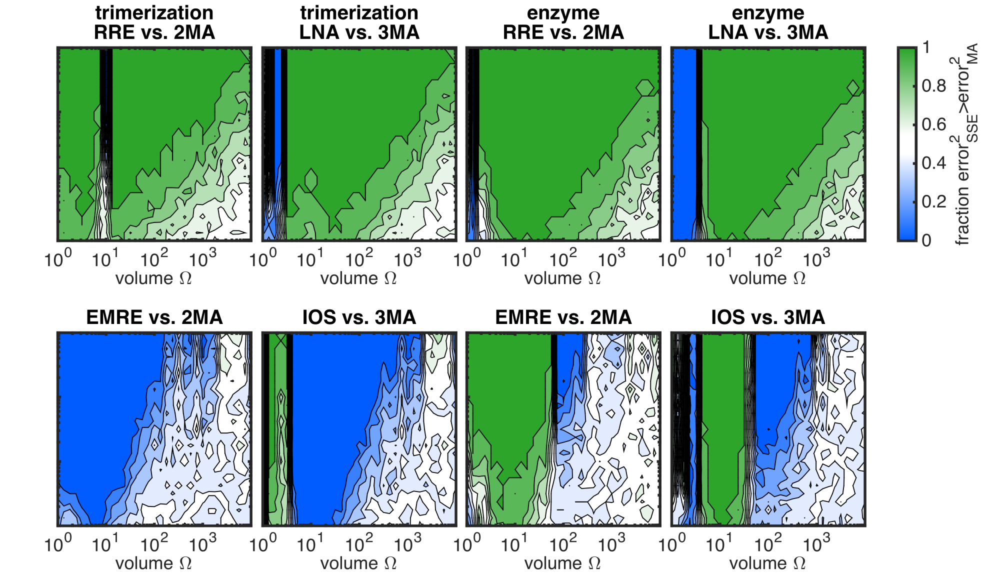

Figure 6. Qualitative comparison between estimation errors of SSE and MA for the trimerization and enzymatic degradation model. Coloring indicates the fraction of datasets for which MA resulted in a smaller error than SSE. The fraction was computed from 100 datasets for every volume/sample size scenario.

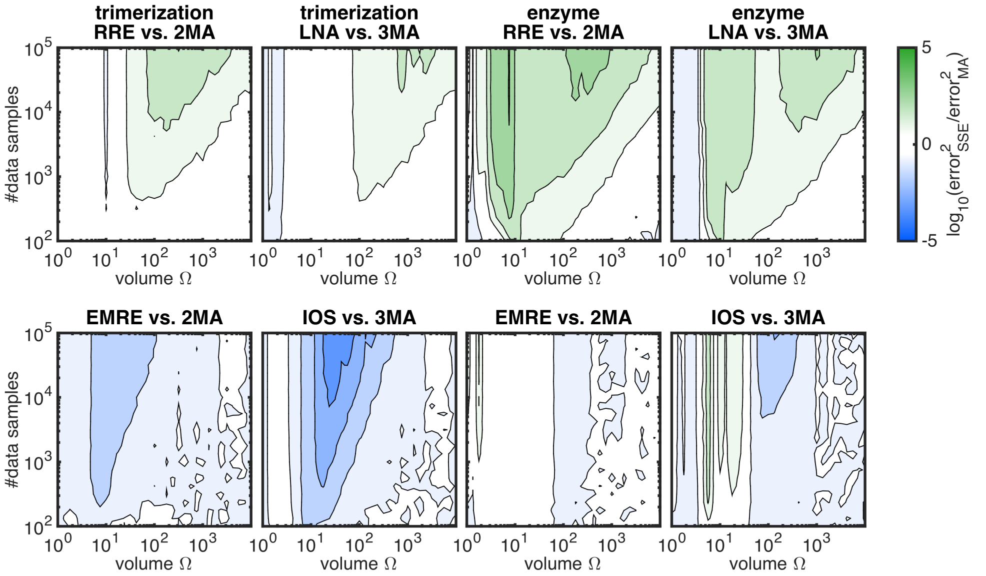

Figure 7. Quantitative comparison between estimation errors of SSE and MA for the trimerization and enzymatic degradation model. Coloring indicates the median of the logarithm of the ratio between squared MA error and squared SSE error. The median was computed from 100 datasets for every volume/sample size scenario.

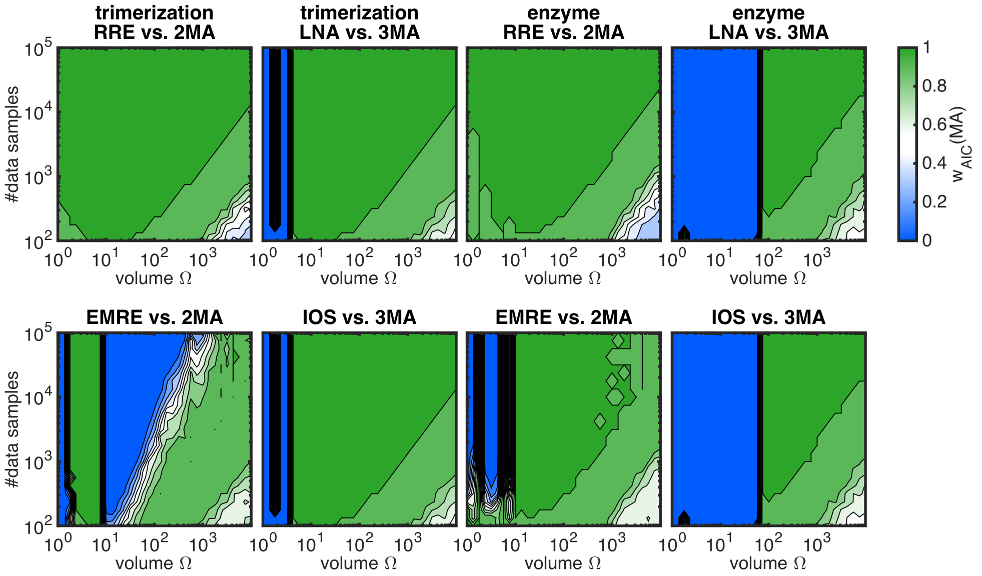

Figure 8. Model selection results with AIC for the trimerization and enzymatic degradation model. Median AIC weight for 2MA and 3MA at respective estimated parameters. A green color indicates that the 2MA and 3MA description is more probable and a blue color indicates the RRE and LNA description is more probable. The median was computed from 100 datasets for every volume/sample size scenario.

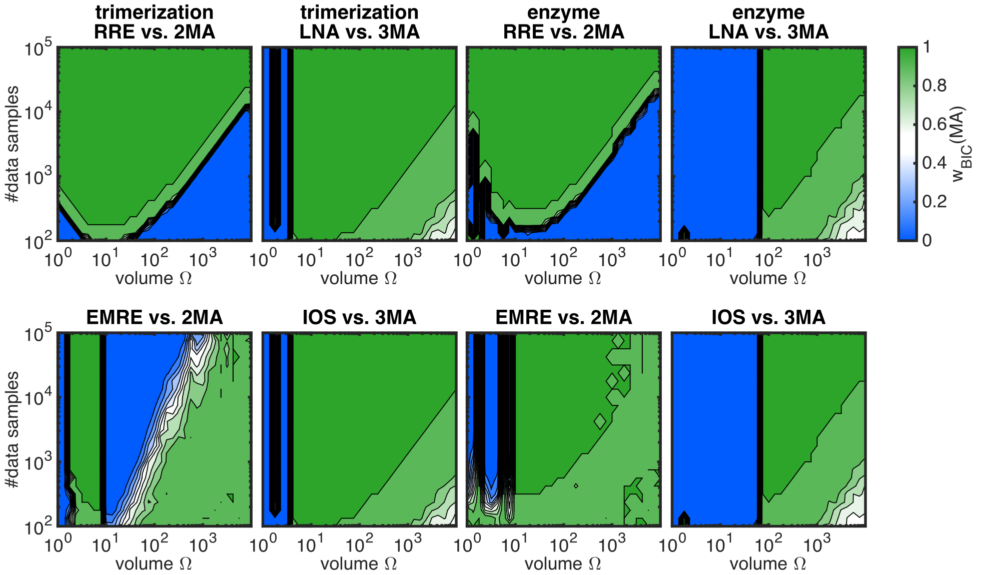

Figure 9. Model selection results with BIC for the trimerization and enzymatic degradation model. Median BIC weight for 2MA and 3MA at respective estimated parameters. A green color indicates that the 2MA and 3MA description is more probable and a blue color indicates the RRE and LNA description is more probable. The median was computed from 100 datasets for every volume/sample size scenario.

References

1. Serban R, Hindmarsh AC. CVODES: An ODE solver with sensitivity analysis capabilities. ACM T Math Software; 2005;31(3):363–396.

2. Hindmarsh AC, Brown PN, Grant KE, Lee SL, Serban R, Shumaker DE, et al. SUNDIALS: Suite of Nonlinear and Differential/Algebraic Equation Solvers. ACM T Math Software; 2005;31(3):363–396.

3. Raue A, Kreutz C, Maiwald T, Bachmann J, Schilling M, Klingmüller U, et al. Structural and practical identifiability analysis of partially observed dynamical models by exploiting the profile likelihood. Bioinformatics; 2009;25(15):1923–1929.

4. Pahle J, Challenger JD, Mendes P, McKane AJ. Biochemical fluctuations, optimisation and the linear noise approximation. BMC Systems Biology; 2012;6(1):86.

5. Challenger JD, McKane AJ, Pahle J. Multi-compartment linear noise approximation. Journal of Statistical Mechanics: Theory and Experiment; 2012;2012(11):P11010.

6. Gomez-Uribe CA, Verghese GC. Mass fluctuation kinetics: Capturing stochastic effects in systems of chemical reactions through coupled mean-variance computations. The Journal of Chemical Physics; 2007;126(2):024109.
