# Supplementary material for: Inference for Stochastic Chemical Kinetics Using Moment Equations and System Size Expansion
Source: PLoS Comput Biol. 2016 Jul 22;12(7):e1005030. doi: 10.1371/journal.pcbi.1005030 (PMC4957800; doi:10.1371/journal.pcbi.1005030)
Supplement: S1 Code — This zip-file contains the MATLAB code for the simulation and application example presented in the paper. We provide implementations of all models, parameter estimation and uncertainty analysis to allow everybody to reproduce the results. (ZIP) [file pcbi.1005030.s002.zip › S1code/cvodewrap/SuiteSparse/AMD/Doc/AMD_UserGuide.pdf]

AMD User Guide

Patrick R. Amestoy[*] Timothy A. Davis[†] Iain S. Duff[‡]

VERSION 2.4.1, Oct 10, 2014

Abstract

AMD is a set of routines that implements the approximate minimum degree ordering algorithm to permute sparse matrices prior to numerical factorization. There are versions written in both C and Fortran 77. A MATLAB interface is included.

AMD Copyright©2013 by Timothy A. Davis, Patrick R. Amestoy, and Iain S. Duff. All Rights Reserved. AMD is available under alternate licences; contact T. Davis for details.

AMD License: Your use or distribution of AMD or any modified version of AMD implies that you agree to this License.

This library is free software; you can redistribute it and/or modify it under the terms of the GNU Lesser General Public License as published by the Free Software Foundation; either version 2.1 of the License, or (at your option) any later version.

This library is distributed in the hope that it will be useful, but WITHOUT ANY WARRANTY; without even the implied warranty of MERCHANTABILITY or FITNESS FOR A PARTICULAR PURPOSE. See the GNU Lesser General Public License for more details.

You should have received a copy of the GNU Lesser General Public License along with this library; if not, write to the Free Software Foundation, Inc., 51 Franklin St, Fifth Floor, Boston, MA 02110-1301 USA

Permission is hereby granted to use or copy this program under the terms of the GNU LGPL, provided that the Copyright, this License, and the Availability of the original version is retained on all copies. User documentation of any code that uses this code or any modified version of this code must cite the Copyright, this License, the Availability note, and "Used by permission." Permission to modify the code and to distribute modified code is granted, provided the Copyright, this License, and the Availability note are retained, and a notice that the code was modified is included.

Availability: http://www.suitesparse.com

Acknowledgments:

This work was supported by the National Science Foundation, under grants ASC-9111263 and DMS-9223088 and CCR-0203270. The conversion to C, the addition of the elimination tree post-ordering, and the handling of dense rows and columns were done while Davis was on sabbatical at Stanford University and Lawrence Berkeley National Laboratory.

[*]ENSEEIHT-IRIT, 2 rue Camichel 31017 Toulouse, France. email: amestoy@enseeiht.fr. http://www.enseeiht.fr/~amestoy.

[†]email: DrTimothyAldenDavis@gmail.com, http://www.suitesparse.com. This work was supported by the National Science Foundation, under grants ASC-9111263, DMS-9223088, and CCR-0203270. Portions of the work were done while on sabbatical at Stanford University and Lawrence Berkeley National Laboratory (with funding from Stanford University and the SciDAC program).

[‡]Rutherford Appleton Laboratory, Chilton, Didcot, Oxon OX11 0QX, England. email: i.s.duff@rl.ac.uk. http://www.numerical.rl.ac.uk/people/isd/isd.html. This work was supported by the EPSRC under grant GR/R46441.

1 Overview

AMD is a set of routines for preordering a sparse matrix prior to numerical factorization. It uses an approximate minimum degree ordering algorithm [1, 2] to find a permutation matrix $\mathbf{P}$ so that the Cholesky factorization $\mathbf{PAP}^\mathsf{T} = \mathbf{LL}^\mathsf{T}$ has fewer (often much fewer) nonzero entries than the Cholesky factorization of $\mathbf{A}$. The algorithm is typically much faster than other ordering methods and minimum degree ordering algorithms that compute an exact degree [4]. Some methods, such as approximate deficiency [9] and graph-partitioning based methods [5, 7, 8, 10] can produce better orderings, depending on the matrix.

The algorithm starts with an undirected graph representation of a symmetric sparse matrix $\mathbf{A}$. Node i in the graph corresponds to row and column i of the matrix, and there is an edge (i, j) in the graph if a_{ij} is nonzero. The degree of a node is initialized to the number of off-diagonal nonzeros in row i, which is the size of the set of nodes adjacent to i in the graph.

The selection of a pivot a_{ii} from the diagonal of $\mathbf{A}$ and the first step of Gaussian elimination corresponds to one step of graph elimination. Numerical fill-in causes new nonzero entries in the matrix (fill-in refers to nonzeros in $\mathbf{L}$ that are not in $\mathbf{A}$). Node i is eliminated and edges are added to its neighbors so that they form a clique (or *element*). To reduce fill-in, node i is selected as the node of least degree in the graph. This process repeats until the graph is eliminated.

The clique is represented implicitly. Rather than listing all the new edges in the graph, a single list of nodes is kept which represents the clique. This list corresponds to the nonzero pattern of the first column of $\mathbf{L}$. As the elimination proceeds, some of these cliques become subsets of subsequent cliques, and are removed. This graph can be stored in place, that is using the same amount of memory as the original graph.

The most costly part of the minimum degree algorithm is the recomputation of the degrees of nodes adjacent to the current pivot element. Rather than keep track of the exact degree, the approximate minimum degree algorithm finds an upper bound on the degree that is easier to compute. For nodes of least degree, this bound tends to be tight. Using the approximate degree instead of the exact degree leads to a substantial savings in run time, particularly for very irregularly structured matrices. It has no effect on the quality of the ordering.

In the C version of AMD, the elimination phase is followed by an elimination tree post-ordering. This has no effect on fill-in, but reorganizes the ordering so that the subsequent numerical factorization is more efficient. It also includes a pre-processing phase in which nodes of very high degree are removed (without causing fill-in), and placed last in the permutation $\mathbf{P}$. This reduces the run time substantially if the matrix has a few rows with many nonzero entries, and has little effect on the quality of the ordering. The C version operates on the symmetric nonzero pattern of $\mathbf{A}+\mathbf{A}^\mathsf{T}$, so it can be given an unsymmetric matrix, or either the lower or upper triangular part of a symmetric matrix.

The two Fortran versions of AMD are essentially identical to two versions of the AMD algorithm discussed in an earlier paper [1] (approximate minimum external degree, both with and without aggressive absorption). For a discussion of the long history of the minimum degree algorithm, see [4].

2 Availability

In addition to appearing as a Collected Algorithm of the ACM,
AMD is available at http://www.suitesparse.com. The Fortran version is available as the routine `MC47` in HSL (formerly the Harwell Subroutine Library) [6].

3 Using AMD in MATLAB

The MATLAB function `amd` is now a built-in function in MATLAB 7.3 (R2006b). The built-in `amd` and the `amd2` function provided here differ in how the optional parameters are passed (the 2nd input parameter).

To use AMD2 in MATLAB, you must first compile the AMD2 mexFunction. Just type `make` in the Unix system shell, while in the `AMD/MATLAB` directory. You can also type `amd_make` in MATLAB, while in the `AMD/MATLAB` directory. Place the `AMD/MATLAB` directory in your MATLAB path. This can be done on any system with MATLAB, including Windows. See Section 8 for more details on how to install AMD.

The MATLAB statement `p=amd(A)` finds a permutation vector `p` such that the Cholesky factorization `chol(A(p,p))` is typically sparser than `chol(A)`. If `A` is unsymmetric, `amd(A)` is identical to `amd(A+A')` (ignoring numerical cancellation). If `A` is not symmetric positive definite, but has substantial diagonal entries and a mostly symmetric nonzero pattern, then this ordering is also suitable for LU factorization. A partial pivoting threshold may be required to prevent pivots from being selected off the diagonal, such as the statement `[L,U,P] = lu (A (p,p), 0.1)`. Type `help lu` for more details. The statement `[L,U,P,Q] = lu (A (p,p))` in MATLAB 6.5 is not suitable, however, because it uses UMFPACK Version 4.0 and thus does not attempt to select pivots from the diagonal. UMFPACK Version 4.1 in MATLAB 7.0 and later uses several strategies, including a symmetric pivoting strategy, and will give you better results if you want to factorize an unsymmetric matrix of this type. Refer to the UMFPACK User Guide for more details, at http://www.suitesparse.com.

The AMD mexFunction is much faster than the built-in MATLAB symmetric minimum degree ordering methods, SYMAMD and SYMMMD. Its ordering quality is comparable to SYMAMD, and better than SYMMMD [3].

An optional input argument can be used to modify the control parameters for AMD (aggressive absorption, dense row/column handling, and printing of statistics). An optional output argument provides statistics on the ordering, including an analysis of the fill-in and the floating-point operation count for a subsequent factorization. For more details (once AMD is installed), type `help amd` in the MATLAB command window.

4 Using AMD in a C program

The C-callable AMD library consists of seven user-callable routines and one include file. There are two versions of each of the routines, with `int` and `long` integers. The routines with prefix `amd_l_` use `long` integer arguments; the others use `int` integer arguments. If you compile AMD in the standard ILP32 mode (32-bit `int`'s, `long`'s, and pointers) then the versions are essentially identical. You will be able to solve problems using up to 2GB of memory. If you compile AMD in the standard LP64 mode, the size of an `int` remains 32-bits, but the size of a `long` and a pointer both get promoted to 64-bits.

The following routines are fully described in Section 9:

- `amd_order` (`long` version: `amd_l_order`)

```
#include "amd.h"
int n, Ap [n+1], Ai [nz], P [n] ;
double Control [AMD_CONTROL], Info [AMD_INFO] ;
int result = amd_order (n, Ap, Ai, P, Control, Info) ;
```

Computes the approximate minimum degree ordering of an n-by-n matrix $\mathbf{A}$. Returns a permutation vector `P` of size `n`, where `P[k] = i` if row and column `i` are the `k`th row and column in the permuted matrix. This routine allocates its own memory of size $1.2e + 9n$ integers, where e is the number of nonzeros in $\mathbf{A} + \mathbf{A}^\mathsf{T}$. It computes statistics about the matrix $\mathbf{A}$, such as the symmetry of its nonzero pattern, the number of nonzeros in $\mathbf{L}$, and the number of floating-point operations required for Cholesky and LU factorizations (which are returned in the `Info` array). The user's input matrix is not modified. It returns `AMD_OK` if successful, `AMD_OK_BUT_JUMBLED` if successful (but the matrix had unsorted and/or duplicate row indices), `AMD_INVALID` if the matrix is invalid, `AMD_OUT_OF_MEMORY` if out of memory.

- `amd_defaults` (`long` version: `amd_l_defaults`)

```
#include "amd.h"
double Control [AMD_CONTROL] ;
amd_defaults (Control) ;
```

Sets the default control parameters in the `Control` array. These can then be modified as desired before passing the array to the other AMD routines.

- `amd_control` (`long` version: `amd_l_control`)

```
#include "amd.h"
double Control [AMD_CONTROL] ;
amd_control (Control) ;
```

Prints a description of the control parameters, and their values.

- `amd_info` (`long` version: `amd_l_info`)

```
#include "amd.h"
double Info [AMD_INFO] ;
amd_info (Info) ;
```

Prints a description of the statistics computed by AMD, and their values.

- `amd_valid` (`long` version: `amd_valid`)

```
#include "amd.h"
int n, Ap [n+1], Ai [nz] ;
int result = amd_valid (n, n, Ap, Ai) ;
```

Returns `AMD_OK` or `AMD_OK_BUT_JUMBLED` if the matrix is valid as input to `amd_order`; the latter is returned if the matrix has unsorted and/or duplicate row indices in one or more columns. Returns `AMD_INVALID` if the matrix cannot be passed to `amd_order`. For `amd_order`, the matrix must also be square. The first two arguments are the number of rows and the number of columns of the matrix. For its use in AMD, these must both equal `n`.

- `amd_2` (`long` version: `amd_l2`) AMD ordering kernel. It is faster than `amd_order`, and can be called by the user, but it is difficult to use. It does not check its inputs for errors. It does not require the columns of its input matrix to be sorted, but it destroys the matrix on output. Additional workspace must be passed. Refer to the source file `AMD/Source/amd_2.c` for a description.

The nonzero pattern of the matrix **A** is represented in compressed column form. For an n-by-n matrix **A** with `nz` nonzero entries, the representation consists of two arrays: `Ap` of size `n+1` and `Ai` of size `nz`. The row indices of entries in column `j` are stored in `Ai[Ap[j]` ... `Ap[j+1]-1]`. For `amd_order`, if duplicate row indices are present, or if the row indices in any given column are not sorted in ascending order, then `amd_order` creates an internal copy of the matrix with sorted rows and no duplicate entries, and orders the copy. This adds slightly to the time and memory usage of `amd_order`, but is not an error condition.

The matrix is 0-based, and thus row indices must be in the range `0` to `n-1`. The first entry `Ap[0]` must be zero. The total number of entries in the matrix is thus `nz = Ap[n]`.

The matrix must be square, but it does not need to be symmetric. The `amd_order` routine constructs the nonzero pattern of $\mathbf{B} = \mathbf{A} + \mathbf{A}^{\mathsf{T}}$ (without forming $\mathbf{A}^{\mathsf{T}}$ explicitly if **A** has sorted columns and no duplicate entries), and then orders the matrix **B**. Thus, either the lower triangular part of **A**, the upper triangular part, or any combination may be passed. The transpose $\mathbf{A}^{\mathsf{T}}$ may also be passed to `amd_order`. The diagonal entries may be present, but are ignored.

4.1 Control parameters

Control parameters are set in an optional `Control` array. It is optional in the sense that if a `NULL` pointer is passed for the `Control` input argument, then default control parameters are used.

- `Control[AMD_DENSE]` (or `Control(1)` in MATLAB): controls the threshold for "dense" rows/columns. A dense row/column in $\mathbf{A} + \mathbf{A}^{\mathsf{T}}$ can cause AMD to spend significant time in ordering the matrix. If `Control[AMD_DENSE]` ≥ 0, rows/columns with more than `Control[AMD_DENSE]` $\sqrt{n}$ entries are ignored during the ordering, and placed last in the output order. The default value of `Control[AMD_DENSE]` is 10. If negative, no rows/columns are treated as "dense." Rows/columns with 16 or fewer off-diagonal entries are never considered "dense."

- `Control[AMD_AGGRESSIVE]` (or `Control(2)` in MATLAB): controls whether or not to use aggressive absorption, in which a prior element is absorbed into the current element if it is a subset of the current element, even if it is not adjacent to the current pivot element (refer to [1, 2] for more details). The default value is nonzero, which means that aggressive absorption will be performed. This nearly always leads to a better ordering (because the approximate degrees are more accurate) and a lower execution time. There are cases where it can lead to a slightly worse ordering, however. To turn it off, set `Control[AMD_AGGRESSIVE]` to 0.

Statistics are returned in the `Info` array (if `Info` is `NULL`, then no statistics are returned). Refer to `amd.h` file, for more details (14 different statistics are returned, so the list is not included here).

4.2 Sample C program

The following program, `amd_demo.c`, illustrates the basic use of AMD. See Section 5 for a short description of each calling sequence.

```
#include <stdio.h>
#include "amd.h"

int n = 5 ;
int Ap [ ] = { 0,   2,       6,       10,  12, 14} ;
int Ai [ ] = { 0,1, 0,1,2,4, 1,2,3,4, 2,3, 1,4   } ;
int P [5] ;
```

```
int main (void)
{
    int k ;
    (void) amd_order (n, Ap, Ai, P, (double *) NULL, (double *) NULL) ;
    for (k = 0 ; k < n ; k++) printf ("P [%d] = %d\n", k, P [k]) ;
    return (0) ;
}
```

The `Ap` and `Ai` arrays represent the binary matrix

$$\mathbf{A} = \left[\begin{array}{ccccc} 1 & 1 & 0 & 0 & 0 \\ 1 & 1 & 1 & 0 & 1 \\ 0 & 1 & 1 & 1 & 0 \\ 0 & 0 & 1 & 1 & 0 \\ 0 & 1 & 1 & 0 & 1 \end{array} \right].$$

The diagonal entries are ignored. AMD constructs the pattern of $\mathbf{A}+\mathbf{A}^{\mathsf{T}}$, and returns a permutation vector of $(0,3,1,4,2)$. Since the matrix is unsymmetric but with a mostly symmetric nonzero pattern, this would be a suitable permutation for an LU factorization of a matrix with this nonzero pattern and whose diagonal entries are not too small. The program uses default control settings and does not return any statistics about the ordering, factorization, or solution (`Control` and `Info` are both `(double *) NULL`). It also ignores the status value returned by `amd_order`.

More example programs are included with the AMD package. The `amd_demo.c` program provides a more detailed demo of AMD. Another example is the AMD mexFunction, `amd_mex.c`.

4.3 A note about zero-sized arrays

AMD uses several user-provided arrays of size `n` or `nz`. Either `n` or `nz` can be zero. If you attempt to `malloc` an array of size zero, however, `malloc` will return a null pointer which AMD will report as invalid. If you `malloc` an array of size `n` or `nz` to pass to AMD, make sure that you handle the `n` = 0 and `nz = 0` cases correctly.

5 Synopsis of C-callable routines

The matrix $\mathbf{A}$ is `n`-by-`n` with `nz` entries.

```
#include "amd.h"
int n, status, Ap [n+1], Ai [nz], P [n] ;
double Control [AMD_CONTROL], Info [AMD_INFO] ;
amd_defaults (Control) ;
status = amd_order (n, Ap, Ai, P, Control, Info) ;
amd_control (Control) ;
amd_info (Info) ;
status = amd_valid (n, n, Ap, Ai) ;
```

The `amd_l_*` routines are identical, except that all `int` arguments become `long`:

```
#include "amd.h"
long n, status, Ap [n+1], Ai [nz], P [n] ;
double Control [AMD_CONTROL], Info [AMD_INFO] ;
amd_l_defaults (Control) ;
status = amd_l_order (n, Ap, Ai, P, Control, Info) ;
amd_l_control (Control) ;
amd_l_info (Info) ;
status = amd_l_valid (n, n, Ap, Ai) ;
```

6 Using AMD in a Fortran program

Two Fortran versions of AMD are provided. The `AMD` routine computes the approximate minimum degree ordering, using aggressive absorption. The `AMDBAR` routine is identical, except that it does not perform aggressive absorption. The `AMD` routine is essentially identical to the HSL routine `MC47B/BD`. Note that earlier versions of the Fortran `AMD` and `AMDBAR` routines included an `IOVFLO` argument, which is no longer present.

In contrast to the C version, the Fortran routines require a symmetric nonzero pattern, with no diagonal entries present although the `MC47A/AD` wrapper in HSL allows duplicates, ignores out-of-range entries, and only uses entries from the upper triangular part of the matrix. Although we have an experimental Fortran code for treating "dense" rows, the Fortran codes in this release do not treat "dense" rows and columns of **A** differently, and thus their run time can be high if there are a few dense rows and columns in the matrix. They do not perform a post-ordering of the elimination tree, compute statistics on the ordering, or check the validity of their input arguments. These facilities are provided by `MC47A/AD` and other subroutines from HSL. Only one `integer` version of each Fortran routine is provided. Both Fortran routines overwrite the user's input matrix, in contrast to the C version. The C version does not return the elimination or assembly tree. The Fortran version returns an assembly tree; refer to the User Guide for details. The following is the syntax of the `AMD` Fortran routine. The `AMDBAR` routine is identical except for the routine name.

```
      INTEGER N, IWLEN, PFREE, NCMPA, IW (IWLEN), PE (N), DEGREE (N), NV (N),
     $         NEXT (N), LAST (N), HEAD (N), ELEN (N), W (N), LEN (N)
      CALL AMD (N, PE, IW, LEN, IWLEN, PFREE, NV, NEXT,
     $         LAST, HEAD, ELEN, DEGREE, NCMPA, W)
      CALL AMDBAR (N, PE, IW, LEN, IWLEN, PFREE, NV, NEXT,
     $         LAST, HEAD, ELEN, DEGREE, NCMPA, W)
```

The input matrix is provided to `AMD` and `AMDBAR` in three arrays, `PE`, of size `N`, `LEN`, of size `N`, and `IW`, of size `IWLEN`. The size of `IW` must be at least `NZ+N`. The recommended size is `1.2*NZ + N`. On input, the indices of nonzero entries in row `I` are stored in `IW`. `PE(I)` is the index in `IW` of the start of row `I`. `LEN(I)` is the number of entries in row `I`. The matrix is 1-based, with row and column indices in the range 1 to `N`. Row `I` is contained in `IW (PE(I)` ... `PE(I) + LEN(I) - 1)`. The diagonal entries must not be present. The indices within each row must not contain any duplicates, but they need not be sorted. The rows themselves need not be in any particular order, and there may be empty space between the rows. If `LEN(I)` is zero, then there are no off-diagonal entries in row `I`, and `PE(I)` is ignored. The integer `PFREE` defines what part of `IW` contains the user's input matrix, which is held in `IW(1` ... `PFREE-1)`. The contents of `IW` and `LEN` are undefined on output, and `PE` is modified to contain information about the ordering.

As the algorithm proceeds, it modifies the `IW` array, placing the pattern of the partially eliminated matrix in `IW(PFREE` ... `IWLEN)`. If this space is exhausted, the space is compressed. The number of compressions performed on the `IW` array is returned in the scalar `NCMPA`. The value of `PFREE` on output is the length of `IW` required for no compressions to be needed.

The output permutation is returned in the array `LAST`, of size `N`. If `I=LAST(K)`, then `I` is the `K`th row in the permuted matrix. The inverse permutation is returned in the array `ELEN`, where `K=ELEN(I)` if `I` is the `K`th row in the permuted matrix. On output, the `PE` and `NV` arrays hold the assembly tree, a supernodal elimination tree that represents the relationship between columns of the Cholesky factor **L**. If `NV(I)` > 0, then `I` is a node in the assembly tree, and the parent of `I` is `-PE(I)`. If `I` is a root of the tree, then `PE(I)` is zero. The value of `NV(I)` is the number of entries in the corresponding column of **L**, including the diagonal. If `NV(I)` is zero, then `I` is a non-principal node that is not in the assembly tree. Node `-PE(I)` is the parent of node `I` in a subtree, the root

of which is a node in the assembly tree. All nodes in one subtree belong to the same supernode in the assembly tree. The other size N arrays (DEGREE, HEAD, NEXT, and W) are used as workspace, and are not defined on input or output.

If you want to use a simpler user-interface and compute the elimination tree post-ordering, you should be able to call the C routines amd_order or amd_l_order from a Fortran program. Just be sure to take into account the 0-based indexing in the P, Ap, and Ai arguments to amd_order and amd_l_order. A sample interface is provided in the files AMD/Demo/amd_f77cross.f and AMD/Demo/amd_f77wrapper.c. To compile the amd_f77cross program, type make cross in the AMD/Demo directory. The Fortran-to-C calling conventions are highly non-portable, so this example is not guaranteed to work with your compiler C and Fortran compilers. The output of amd_f77cross is in amd_f77cross.out.

7 Sample Fortran main program

The following program illustrates the basic usage of the Fortran version of AMD. The AP and AI arrays represent the binary matrix

$$\mathbf{A} = \begin{bmatrix} 1 & 1 & 0 & 0 & 0 \\ 1 & 1 & 1 & 0 & 1 \\ 0 & 1 & 1 & 1 & 1 \\ 0 & 0 & 1 & 1 & 0 \\ 0 & 1 & 1 & 0 & 1 \end{bmatrix}$$

in a conventional 1-based column-oriented form, except that the diagonal entries are not present. The matrix has the same as nonzero pattern of $\mathbf{A}+\mathbf{A}^{\mathsf{T}}$ in the C program, in Section 4. The output permutation is $(4, 1, 3, 5, 2)$. It differs from the permutation returned by the C routine amd_order because a post-order of the elimination tree has not yet been performed.

```
        INTEGER N, NZ, J, K, P, IWLEN, PFREE, NCMPA
        PARAMETER (N = 5, NZ = 10, IWLEN = 17)
        INTEGER AP (N+1), AI (NZ), LAST (N), PE (N), LEN (N), ELEN (N),
     $      IW (IWLEN), DEGREE (N), NV (N), NEXT (N), HEAD (N), W (N)
        DATA AP / 1, 2,     5,     8,  9,  11/
        DATA AI / 2, 1,3,5, 2,4,5, 3,  2,3   /
C       load the matrix into the AMD workspace
        DO 10 J = 1,N
            PE (J) = AP (J)
            LEN (J) = AP (J+1) - AP (J)
10      CONTINUE
        DO 20 P = 1,NZ
            IW (P) = AI (P)
20      CONTINUE
        PFREE = NZ + 1
C       order the matrix (destroys the copy of A in IW, PE, and LEN)
        CALL AMD (N, PE, IW, LEN, IWLEN, PFREE, NV, NEXT, LAST, HEAD,
     $      ELEN, DEGREE, NCMPA, W)
        DO 60 K = 1, N
            PRINT 50, K, LAST (K)
50          FORMAT ('P (',I2,') = ', I2)
60      CONTINUE
        END
```

The Demo directory contains an example of how the C version may be called from a Fortran program, but this is highly non-portable. For this reason, it is placed in the Demo directory, not in the primary Source directory.

8 Installation

The following discussion assumes you have the make program, either in Unix, or in Windows with Cygwin.

System-dependent configurations are in the ../SuiteSparse_config/SuiteSparse_config.mk file. You can edit that file to customize the compilation. The default settings will work on most systems. Sample configuration files are provided for Linux, Sun Solaris, SGI IRIX, IBM AIX, and the DEC/Compaq Alpha.

To compile and install the C-callable AMD library, go to the AMD directory and type make. The library will be placed in AMD/Lib/libamd.a. Three demo programs of the AMD ordering routine will be compiled and tested in the AMD/Demo directory. The outputs of these demo programs will then be compared with output files in the distribution.

To compile and install the Fortran-callable AMD library, go to the AMD directory and type make fortran. The library will be placed in AMD/Lib/libamdf77.a. A demo program will be compiled and tested in the AMD/Demo directory. The output will be compared with an output file in the distribution.

Typing make clean will remove all but the final compiled libraries and demo programs. Typing make purge or make distclean removes all files not in the original distribution. If you compile AMD and then later change the ../SuiteSparse_config/SuiteSparse_config.mk file then you should type make purge and then make to recompile.

When you compile your program that uses the C-callable AMD library, you need to add the AMD/Lib/libamd.a library and you need to tell your compiler to look in the AMD/Include directory for include files. To compile a Fortran program that calls the Fortran AMD library, you need to add the AMD/Lib/libamdf77.a library. See AMD/Demo/Makefile for an example.

If all you want to use is the AMD2 mexFunction in MATLAB, you can skip the use of the make command entirely. Simply type amd_make in MATLAB while in the AMD/MATLAB directory. This works on any system with MATLAB, including Windows. Alternately, type make in the AMD/MATLAB directory, or just use the built-in amd in MATLAB 7.3 or later.

If you are including AMD as a subset of a larger library and do not want to link the C standard I/O library, or if you simply do not need to use them, you can safely remove the amd_control.c and amd_info.c files. Similarly, if you use default parameters (or define your own Control array), then you can exclude the amd_defaults.c file. Each of these files contains the user-callable routines of the same name. None of these auxiliary routines are directly called by amd_order. The amd_dump.c file contains debugging routines that are neither used nor compiled unless debugging is enabled. The amd_internal.h file must be edited to enable debugging; refer to the instructions in that file. The bare minimum files required to use just amd_order are amd.h and amd_internal.h in the Include directory, and amd_1.c, amd_2.c, amd_aat.c, amd_global.c, and_order.c, amd_postorder.c, amd_post_tree.c, amd_preprocess.c, and amd_valid.c in the Source directory.

9 The AMD routines

The file AMD/Include/amd.h listed below describes each user-callable routine in the C version of AMD, and gives details on their use.

```
/* ========================================================================= */
/* === AMD:  approximate minimum degree ordering =========================== */
/* ========================================================================= */

/* ------------------------------------------------------------------------- */
/* AMD Version 2.4, Copyright (c) 1996-2013 by Timothy A. Davis,             */
/* Patrick R. Amestoy, and Iain S. Duff.  See ../README.txt for License.     */
/* email: DrTimothyAldenDavis@gmail.com                                      */
/* ------------------------------------------------------------------------- */

/* AMD finds a symmetric ordering P of a matrix A so that the Cholesky
 * factorization of P*A*P' has fewer nonzeros and takes less work than the
 * Cholesky factorization of A.  If A is not symmetric, then it performs its
 * ordering on the matrix A+A'.  Two sets of user-callable routines are
 * provided, one for int integers and the other for SuiteSparse_long integers.
 *
 * The method is based on the approximate minimum degree algorithm, discussed
 * in Amestoy, Davis, and Duff, "An approximate degree ordering algorithm",
 * SIAM Journal of Matrix Analysis and Applications, vol. 17, no. 4, pp.
 * 886-905, 1996.  This package can perform both the AMD ordering (with
 * aggressive absorption), and the AMDBAR ordering (without aggressive
 * absorption) discussed in the above paper.  This package differs from the
 * Fortran codes discussed in the paper:
 *
 *       (1) it can ignore "dense" rows and columns, leading to faster run times
 *       (2) it computes the ordering of A+A' if A is not symmetric
 *       (3) it is followed by a depth-first post-ordering of the assembly tree
 *           (or supernodal elimination tree)
 *
 * For historical reasons, the Fortran versions, amd.f and amdbar.f, have
 * been left (nearly) unchanged.  They compute the identical ordering as
 * described in the above paper.
 */

#ifndef AMD_H
#define AMD_H

/* make it easy for C++ programs to include AMD */
#ifdef __cplusplus
extern "C" {
#endif

/* get the definition of size_t: */
#include <stddef.h>

#include "SuiteSparse_config.h"

int amd_order                  /* returns AMD_OK, AMD_OK_BUT_JUMBLED,
                                 * AMD_INVALID, or AMD_OUT_OF_MEMORY */
(
    int n,                     /* A is n-by-n.  n must be >= 0. */
    const int Ap [ ],          /* column pointers for A, of size n+1 */
    const int Ai [ ],          /* row indices of A, of size nz = Ap [n] */
    int P [ ],                 /* output permutation, of size n */
```

```
    double Control [ ],         /* input Control settings, of size AMD_CONTROL */
    double Info [ ]             /* output Info statistics, of size AMD_INFO */
) ;

SuiteSparse_long amd_l_order    /* see above for description of arguments */
(
    SuiteSparse_long n,
    const SuiteSparse_long Ap [ ],
    const SuiteSparse_long Ai [ ],
    SuiteSparse_long P [ ],
    double Control [ ],
    double Info [ ]
) ;

/* Input arguments (not modified):
 *
 *       n: the matrix A is n-by-n.
 *       Ap: an int/SuiteSparse_long array of size n+1, containing column
 *              pointers of A.
 *       Ai: an int/SuiteSparse_long array of size nz, containing the row
 *              indices of A, where nz = Ap [n].
 *       Control:  a double array of size AMD_CONTROL, containing control
 *           parameters.  Defaults are used if Control is NULL.
 *
 * Output arguments (not defined on input):
 *
 *       P: an int/SuiteSparse_long array of size n, containing the output
 *           permutation. If row i is the kth pivot row, then P [k] = i.  In
 *           MATLAB notation, the reordered matrix is A (P,P).
 *       Info: a double array of size AMD_INFO, containing statistical
 *           information.  Ignored if Info is NULL.
 *
 * On input, the matrix A is stored in column-oriented form.  The row indices
 * of nonzero entries in column j are stored in Ai [Ap [j] ... Ap [j+1]-1].
 *
 * If the row indices appear in ascending order in each column, and there
 * are no duplicate entries, then amd_order is slightly more efficient in
 * terms of time and memory usage.  If this condition does not hold, a copy
 * of the matrix is created (where these conditions do hold), and the copy is
 * ordered.  This feature is new to v2.0 (v1.2 and earlier required this
 * condition to hold for the input matrix).
 *
 * Row indices must be in the range 0 to
 * n-1.  Ap [0] must be zero, and thus nz = Ap [n] is the number of nonzeros
 * in A.  The array Ap is of size n+1, and the array Ai is of size nz = Ap [n].
 * The matrix does not need to be symmetric, and the diagonal does not need to
 * be present (if diagonal entries are present, they are ignored except for
 * the output statistic Info [AMD_NZDIAG]).  The arrays Ai and Ap are not
 * modified.  This form of the Ap and Ai arrays to represent the nonzero
 * pattern of the matrix A is the same as that used internally by MATLAB.
 * If you wish to use a more flexible input structure, please see the
 * umfpack_*_triplet_to_col routines in the UMFPACK package, at
 * http://www.suitesparse.com.
 *
 * Restrictions:  n >= 0.  Ap [0] = 0.  Ap [j] <= Ap [j+1] for all j in the
 *       range 0 to n-1.  nz = Ap [n] >= 0.  Ai [0..nz-1] must be in the range 0
 *       to n-1.  Finally, Ai, Ap, and P must not be NULL.  If any of these
 *       restrictions are not met, AMD returns AMD_INVALID.
 *
```

```
* AMD returns:
*
*       AMD_OK if the matrix is valid and sufficient memory can be allocated to
*           perform the ordering.
*
*       AMD_OUT_OF_MEMORY if not enough memory can be allocated.
*
*       AMD_INVALID if the input arguments n, Ap, Ai are invalid, or if P is
*           NULL.
*
*       AMD_OK_BUT_JUMBLED if the matrix had unsorted columns, and/or duplicate
*           entries, but was otherwise valid.
*
* The AMD routine first forms the pattern of the matrix A+A', and then
* computes a fill-reducing ordering, P.  If P [k] = i, then row/column i of
* the original is the kth pivotal row.  In MATLAB notation, the permuted
* matrix is A (P,P), except that 0-based indexing is used instead of the
* 1-based indexing in MATLAB.
*
* The Control array is used to set various parameters for AMD.  If a NULL
* pointer is passed, default values are used.  The Control array is not
* modified.
*
*       Control [AMD_DENSE]:  controls the threshold for "dense" rows/columns.
*           A dense row/column in A+A' can cause AMD to spend a lot of time in
*           ordering the matrix.  If Control [AMD_DENSE] >= 0, rows/columns
*           with more than Control [AMD_DENSE] * sqrt (n) entries are ignored
*           during the ordering, and placed last in the output order.  The
*           default value of Control [AMD_DENSE] is 10.  If negative, no
*           rows/columns are treated as "dense".  Rows/columns with 16 or
*           fewer off-diagonal entries are never considered "dense".
*
*       Control [AMD_AGGRESSIVE]: controls whether or not to use aggressive
*           absorption, in which a prior element is absorbed into the current
*           element if is a subset of the current element, even if it is not
*           adjacent to the current pivot element (refer to Amestoy, Davis,
*           & Duff, 1996, for more details).  The default value is nonzero,
*           which means to perform aggressive absorption.  This nearly always
*           leads to a better ordering (because the approximate degrees are
*           more accurate) and a lower execution time.  There are cases where
*           it can lead to a slightly worse ordering, however.  To turn it off,
*           set Control [AMD_AGGRESSIVE] to 0.
*
*       Control [2..4] are not used in the current version, but may be used in
*           future versions.
*
* The Info array provides statistics about the ordering on output.  If it is
* not present, the statistics are not returned.  This is not an error
* condition.
*
*       Info [AMD_STATUS]:  the return value of AMD, either AMD_OK,
*           AMD_OK_BUT_JUMBLED, AMD_OUT_OF_MEMORY, or AMD_INVALID.
*
*       Info [AMD_N]: n, the size of the input matrix
*
*       Info [AMD_NZ]: the number of nonzeros in A, nz = Ap [n]
*
*       Info [AMD_SYMMETRY]:  the symmetry of the matrix A.  It is the number
*           of "matched" off-diagonal entries divided by the total number of
```

```
 *          off-diagonal entries.  An entry A(i,j) is matched if A(j,i) is also
 *          an entry, for any pair (i,j) for which i != j.  In MATLAB notation,
 *               S = spones (A) ;
 *               B = tril (S, -1) + triu (S, 1) ;
 *               symmetry = nnz (B & B') / nnz (B) ;
 *
 *      Info [AMD_NZDIAG]: the number of entries on the diagonal of A.
 *
 *      Info [AMD_NZ_A_PLUS_AT]:  the number of nonzeros in A+A', excluding the
 *          diagonal.  If A is perfectly symmetric (Info [AMD_SYMMETRY] = 1)
 *          with a fully nonzero diagonal, then Info [AMD_NZ_A_PLUS_AT] = nz-n
 *          (the smallest possible value).  If A is perfectly unsymmetric
 *          (Info [AMD_SYMMETRY] = 0, for an upper triangular matrix, for
 *          example) with no diagonal, then Info [AMD_NZ_A_PLUS_AT] = 2*nz
 *          (the largest possible value).
 *
 *      Info [AMD_NDENSE]: the number of "dense" rows/columns of A+A' that were
 *          removed from A prior to ordering.  These are placed last in the
 *          output order P.
 *
 *      Info [AMD_MEMORY]: the amount of memory used by AMD, in bytes.  In the
 *          current version, this is 1.2 * Info  [AMD_NZ_A_PLUS_AT] + 9*n
 *          times the size of an integer.  This is at most 2.4nz + 9n.  This
 *          excludes the size of the input arguments Ai, Ap, and P, which have
 *          a total size of nz + 2*n + 1 integers.
 *
 *      Info [AMD_NCMPA]: the number of garbage collections performed.
 *
 *      Info [AMD_LNZ]: the number of nonzeros in L (excluding the diagonal).
 *          This is a slight upper bound because mass elimination is combined
 *          with the approximate degree update.  It is a rough upper bound if
 *          there are many "dense" rows/columns.  The rest of the statistics,
 *          below, are also slight or rough upper bounds, for the same reasons.
 *          The post-ordering of the assembly tree might also not exactly
 *          correspond to a true elimination tree postordering.
 *
 *      Info [AMD_NDIV]: the number of divide operations for a subsequent LDL'
 *          or LU factorization of the permuted matrix A (P,P).
 *
 *      Info [AMD_NMULTSUBS_LDL]:  the number of multiply-subtract pairs for a
 *          subsequent LDL' factorization of A (P,P).
 *
 *      Info [AMD_NMULTSUBS_LU]:  the number of multiply-subtract pairs for a
 *          subsequent LU factorization of A (P,P), assuming that no numerical
 *          pivoting is required.
 *
 *      Info [AMD_DMAX]:  the maximum number of nonzeros in any column of L,
 *          including the diagonal.
 *
 *      Info [14..19] are not used in the current version, but may be used in
 *          future versions.
 */

/* ------------------------------------------------------------------------- */
/* direct interface to AMD */
/* ------------------------------------------------------------------------- */

/* amd_2 is the primary AMD ordering routine.  It is not meant to be
 * user-callable because of its restrictive inputs and because it destroys
```

```
 * the user's input matrix.  It does not check its inputs for errors, either.
 * However, if you can work with these restrictions it can be faster than
 * amd_order and use less memory (assuming that you can create your own copy
 * of the matrix for AMD to destroy).  Refer to AMD/Source/amd_2.c for a
 * description of each parameter. */

void amd_2
(
    int n,
    int Pe [ ],
    int Iw [ ],
    int Len [ ],
    int iwlen,
    int pfree,
    int Nv [ ],
    int Next [ ],
    int Last [ ],
    int Head [ ],
    int Elen [ ],
    int Degree [ ],
    int W [ ],
    double Control [ ],
    double Info [ ]
) ;

void amd_l2
(
    SuiteSparse_long n,
    SuiteSparse_long Pe [ ],
    SuiteSparse_long Iw [ ],
    SuiteSparse_long Len [ ],
    SuiteSparse_long iwlen,
    SuiteSparse_long pfree,
    SuiteSparse_long Nv [ ],
    SuiteSparse_long Next [ ],
    SuiteSparse_long Last [ ],
    SuiteSparse_long Head [ ],
    SuiteSparse_long Elen [ ],
    SuiteSparse_long Degree [ ],
    SuiteSparse_long W [ ],
    double Control [ ],
    double Info [ ]
) ;

/* ------------------------------------------------------------------------- */
/* amd_valid */
/* ------------------------------------------------------------------------- */

/* Returns AMD_OK or AMD_OK_BUT_JUMBLED if the matrix is valid as input to
 * amd_order; the latter is returned if the matrix has unsorted and/or
 * duplicate row indices in one or more columns.  Returns AMD_INVALID if the
 * matrix cannot be passed to amd_order.  For amd_order, the matrix must also
 * be square.  The first two arguments are the number of rows and the number
 * of columns of the matrix.  For its use in AMD, these must both equal n.
 *
 * NOTE: this routine returned TRUE/FALSE in v1.2 and earlier.
 */

int amd_valid
```

```
(
    int n_row,                  /* # of rows */
    int n_col,                  /* # of columns */
    const int Ap [ ],           /* column pointers, of size n_col+1 */
    const int Ai [ ]            /* row indices, of size Ap [n_col] */
) ;

SuiteSparse_long amd_l_valid
(
    SuiteSparse_long n_row,
    SuiteSparse_long n_col,
    const SuiteSparse_long Ap [ ],
    const SuiteSparse_long Ai [ ]
) ;

/* ------------------------------------------------------------------------- */
/* AMD memory manager and printf routines */
/* ------------------------------------------------------------------------- */

    /* moved to SuiteSparse_config.c */

/* ------------------------------------------------------------------------- */
/* AMD Control and Info arrays */
/* ------------------------------------------------------------------------- */

/* amd_defaults:  sets the default control settings */
void amd_defaults   (double Control [ ]) ;
void amd_l_defaults (double Control [ ]) ;

/* amd_control: prints the control settings */
void amd_control    (double Control [ ]) ;
void amd_l_control  (double Control [ ]) ;

/* amd_info: prints the statistics */
void amd_info       (double Info [ ]) ;
void amd_l_info     (double Info [ ]) ;

#define AMD_CONTROL 5          /* size of Control array */
#define AMD_INFO 20            /* size of Info array */

/* contents of Control */
#define AMD_DENSE 0            /* "dense" if degree > Control [0] * sqrt (n) */
#define AMD_AGGRESSIVE 1    /* do aggressive absorption if Control [1] != 0 */

/* default Control settings */
#define AMD_DEFAULT_DENSE 10.0          /* default "dense" degree 10*sqrt(n) */
#define AMD_DEFAULT_AGGRESSIVE 1    /* do aggressive absorption by default */

/* contents of Info */
#define AMD_STATUS 0           /* return value of amd_order and amd_l_order */
#define AMD_N 1                /* A is n-by-n */
#define AMD_NZ 2      /* number of nonzeros in A */
#define AMD_SYMMETRY 3         /* symmetry of pattern (1 is sym., 0 is unsym.) */
#define AMD_NZDIAG 4           /* # of entries on diagonal */
#define AMD_NZ_A_PLUS_AT 5  /* nz in A+A' */
#define AMD_NDENSE 6           /* number of "dense" rows/columns in A */
#define AMD_MEMORY 7           /* amount of memory used by AMD */
#define AMD_NCMPA 8            /* number of garbage collections in AMD */
#define AMD_LNZ 9     /* approx. nz in L, excluding the diagonal */
```

```
#define AMD_NDIV 10            /* number of fl. point divides for LU and LDL' */
#define AMD_NMULTSUBS_LDL 11 /* number of fl. point (*,-) pairs for LDL' */
#define AMD_NMULTSUBS_LU 12  /* number of fl. point (*,-) pairs for LU */
#define AMD_DMAX 13             /* max nz. in any column of L, incl. diagonal */

/* ------------------------------------------------------------------------- */
/* return values of AMD */
/* ------------------------------------------------------------------------- */

#define AMD_OK 0           /* success */
#define AMD_OUT_OF_MEMORY -1        /* malloc failed, or problem too large */
#define AMD_INVALID -2              /* input arguments are not valid */
#define AMD_OK_BUT_JUMBLED 1        /* input matrix is OK for amd_order, but
    * columns were not sorted, and/or duplicate entries were present.  AMD had
    * to do extra work before ordering the matrix.  This is a warning, not an
    * error.  */

/* ========================================================================== */
/* === AMD version ========================================================== */
/* ========================================================================== */

/* AMD Version 1.2 and later include the following definitions.
 * As an example, to test if the version you are using is 1.2 or later:
 *
 * #ifdef AMD_VERSION
 *       if (AMD_VERSION >= AMD_VERSION_CODE (1,2)) ...
 * #endif
 *
 * This also works during compile-time:
 *
 *       #if defined(AMD_VERSION) && (AMD_VERSION >= AMD_VERSION_CODE (1,2))
 *           printf ("This is version 1.2 or later\n") ;
 *       #else
 *           printf ("This is an early version\n") ;
 *       #endif
 *
 * Versions 1.1 and earlier of AMD do not include a #define'd version number.
 */

#define AMD_DATE "Oct 10, 2014"
#define AMD_VERSION_CODE(main,sub) ((main) * 1000 + (sub))
#define AMD_MAIN_VERSION 2
#define AMD_SUB_VERSION 4
#define AMD_SUBSUB_VERSION 1
#define AMD_VERSION AMD_VERSION_CODE(AMD_MAIN_VERSION,AMD_SUB_VERSION)

#ifdef __cplusplus
}
#endif

#endif
```

References

[1] P. R. Amestoy, T. A. Davis, and I. S. Duff. An approximate minimum degree ordering algorithm. *SIAM J. Matrix Anal. Applic.*, 17(4):886–905, 1996.

[2] P. R. Amestoy, T. A. Davis, and I. S. Duff. Algorithm 837: An approximate minimum degree ordering algorithm. *ACM Trans. Math. Softw.*, 30(3):381–388, 2004.

[3] T. A. Davis, J. R. Gilbert, S. I. Larimore, and E. G. Ng. A column approximate minimum degree ordering algorithm. *ACM Trans. Math. Softw.*, 30:353–376, 2004.

[4] A. George and J. W. H. Liu. The evolution of the minimum degree ordering algorithm. *SIAM Review*, 31(1):1–19, 1989.

[5] B. Hendrickson and E. Rothberg. Improving the runtime and quality of nested dissection ordering. *SIAM J. Sci. Comput.*, 20:468–489, 1999.

[6] HSL. HSL 2002: A collection of Fortran codes for large scale scientific computation, 2002. `www.cse.clrc.ac.uk/nag/hsl`.

[7] G. Karypis and V. Kumar. A fast and high quality multilevel scheme for partitioning irregular graphs. *SIAM J. Sci. Comput.*, 20:359–392, 1998.

[8] F. Pellegrini, J. Roman, and P. Amestoy. Hybridizing nested dissection and halo approximate minimum degree for efficient sparse matrix ordering. *Concurrency: Practice and Experience*, 12:68–84, 2000.

[9] E. Rothberg and S. C. Eisenstat. Node selection strategies for bottom-up sparse matrix orderings. *SIAM J. Matrix Anal. Applic.*, 19(3):682–695, 1998.

[10] J. Schulze. Towards a tighter coupling of bottom-up and top-down sparse matrix ordering methods. *BIT*, 41(4):800–841, 2001.
