# Supplementary material for: Inference for Stochastic Chemical Kinetics Using Moment Equations and System Size Expansion
Source: PLoS Comput Biol. 2016 Jul 22;12(7):e1005030. doi: 10.1371/journal.pcbi.1005030 (PMC4957800; doi:10.1371/journal.pcbi.1005030)
Supplement: S1 Code — This zip-file contains the MATLAB code for the simulation and application example presented in the paper. We provide implementations of all models, parameter estimation and uncertainty analysis to allow everybody to reproduce the results. (ZIP) [file pcbi.1005030.s002.zip › S1code/cvodewrap/SuiteSparse/KLU/Doc/KLU_UserGuide.pdf]

User Guide for KLU and BTF

Timothy A. Davis*   Eka Palamadai Natarajan

VERSION 1.3.2, Oct 23, 2014

Abstract

KLU is a set of routines for solving sparse linear systems of equations. It is particularly well-suited to matrices arising in SPICE-like circuit simulation applications. It relies on a permutation to block triangular form (BTF), several methods for finding a fill-reducing ordering (variants of approximate minimum degree, and nested dissection), and a sparse left-looking LU factorization method to factorize each block. A MATLAB interface is included. KLU appears as Collected Algorithm 907 of the ACM [9].

*DrTimothyAldenDavis@gmail.com, http://www.suitesparse.com. This work was supported by Sandia National Labs, and the National Science Foundation. Portions of the work were done while on sabbatical at Stanford University and Lawrence Berkeley National Laboratory (with funding from Stanford University and the SciDAC program).

Contents

1 License and Copyright **3**

2 Overview **4**

3 Availability **5**

4 Using KLU and BTF in MATLAB **5**

5 Using KLU and BTF in a C program **6**

5.1 KLU Common object 6
5.2 KLU Symbolic object 8
5.3 KLU Numeric object 8
5.4 A sparse matrix in KLU 8
5.5 `klu_defaults`: set default parameters 9
5.6 `klu_analyze`: order and analyze a matrix 9
5.7 `klu_analyze_given`: order and analyze a matrix 9
5.8 `klu_factor`: numerical factorization 10
5.9 `klu_solve`: solve a linear system 10
5.10 `klu_tsolve`: solve a transposed linear system 11
5.11 `klu_refactor`: numerical refactorization 11
5.12 `klu_free_symbolic`: destroy the `Symbolic` object 12
5.13 `klu_free_numeric`: destroy the `Numeric` object 12
5.14 `klu_sort`: sort the columns of L and U 12
5.15 `klu_flops`: determine the flop count 13
5.16 `klu_rgrowth`: determine the pivot growth 13
5.17 `klu_condest`: accurate condition number estimation 14
5.18 `klu_rcond`: cheap reciprocal condition number estimation 14
5.19 `klu_scale`: scale and check a sparse matrix 15
5.20 `klu_extract`: extract the LU factorization 15
5.21 `klu_malloc`, `klu_free`, `klu_realloc`: memory management 16
5.22 `btf_maxtrans`: maximum transversal 16
5.23 `btf_strongcomp`: strongly connected components 17
5.24 `btf_order`: permutation to block triangular form 17
5.25 Sample C programs that use KLU 18

6 Installation **19**

7 The KLU routines **20**

8 The BTF routines **35**

1 License and Copyright

KLU, Copyright©2004-2011 University of Florida. All Rights Reserved. KLU is available under alternate licenses; contact T. Davis for details.

KLU License: Your use or distribution of KLU or any modified version of KLU implies that you agree to this License.

This library is free software; you can redistribute it and/or modify it under the terms of the GNU Lesser General Public License as published by the Free Software Foundation; either version 2.1 of the License, or (at your option) any later version.

This library is distributed in the hope that it will be useful, but WITHOUT ANY WARRANTY; without even the implied warranty of MERCHANTABILITY or FITNESS FOR A PARTICULAR PURPOSE. See the GNU Lesser General Public License for more details.

You should have received a copy of the GNU Lesser General Public License along with this library; if not, write to the Free Software Foundation, Inc., 51 Franklin St, Fifth Floor, Boston, MA 02110-1301 USA

Permission is hereby granted to use or copy this program under the terms of the GNU LGPL, provided that the Copyright, this License, and the Availability of the original version is retained on all copies. User documentation of any code that uses this code or any modified version of this code must cite the Copyright, this License, the Availability note, and "Used by permission." Permission to modify the code and to distribute modified code is granted, provided the Copyright, this License, and the Availability note are retained, and a notice that the code was modified is included.

Availability: `http://www.suitesparse.com`

Acknowledgments:

This work was supported by Sandia National Laboratories (Mike Heroux) and the National Science Foundation under grants 0203270 and 0620286.

2 Overview

KLU is a set of routines for solving sparse linear systems of equations. It first permutes the matrix into upper block triangular form, via the BTF package. This is done by first finding a permutation for a zero-free diagonal (a maximum transversal) [12]. If there is no such permutation, then the matrix is structurally rank-deficient, and is numerically singular. Next, Tarjan's method [13, 23] is used to find the strongly-connected components of the graph. The block triangular form is essentially unique; any method will lead to the same number and sizes of blocks, although the ordering of the blocks may vary (consider a diagonal matrix, for example). Assuming the matrix has full structural rank, the permuted matrix has the following form:

$$PAQ = \left[\begin{array}{ccc} A_{11} & \cdots & A_{1k} \\ & \ddots & \vdots \\ & & A_{kk} \end{array} \right],$$

where each diagonal block is square with a zero-free diagonal.

Next, each diagonal block is factorized with a sparse left-looking method [14]. The kernel of this factorization method is an efficient method for solving $Lx = b$ when L, x, and b are all sparse. This kernel is used to compute each column of L and U, one column at a time. The total work performed by this method is always proportional to the number of floating-point operations, something that is not true of any other sparse LU factorization method.

Prior to factorizing each diagonal block, the blocks are ordered to reduce fill-in. By default, the symmetric approximate minimum degree (AMD) ordering is used on $A_{ii} + A_{ii}^T$ [1, 2]. Another ordering option is to find a column ordering via COLAMD [7, 8]. Alternatively, a permutation can be provided by the user, or a pointer to a user-provided ordering function can be passed, which is then used to order each block.

Only the diagonal blocks need to be factorized. Consider a linear system where the matrix is permuted into three blocks, for example:

$$\left[\begin{array}{ccc} A_{11} & A_{12} & A_{13} \\ & A_{22} & A_{23} \\ & & A_{33} \end{array} \right] \left[\begin{array}{c} x_1 \\ x_2 \\ x_3 \end{array} \right] = \left[\begin{array}{c} b_1 \\ b_2 \\ b_3 \end{array} \right].$$

The non-singular system $A_{33}x_3 = b_3$ can first be solved for x_3. After a block back substitution, the resulting system becomes

$$\left[\begin{array}{cc} A_{11} & A_{12} \\ & A_{22} \end{array} \right] \left[\begin{array}{c} x_1 \\ x_2 \end{array} \right] = \left[\begin{array}{c} b_1 - A_{13}x_3 \\ b_2 - A_{23}x_3 \end{array} \right] = \left[\begin{array}{c} b'_1 \\ b'_2 \end{array} \right]$$

and the $A_{22}x_2 = b'_2$ system can be solved for x_2. The primary advantage of this method is that no fill-in occurs in the off-diagonal blocks (A_{12}, A_{13}, and A_{23}). This is particular critical for sparse linear systems arising in SPICE-like circuit simulation [18, 19, 20, 22]. Circuit matrices are typically permutable into block triangular form, with many singletons (1-by-1 blocks). They also often have a handful of rows and columns with many nonzero entries, due to voltage and current sources. These rows and columns are pushed into the upper block triangular form, and related to the singleton blocks (for example, A_{33} in the above system is 1-by-1, and the column in A_{13} and A_{23} has many nonzero entries). Since these nearly-dense rows and columns do not appear in the LU factorization of the diagonal blocks, they cause no fill-in.

The structural rank of a matrix is based solely on the pattern of its entries, not their numerical values. With random entries, the two ranks are equal with probability one. The structural rank of

any matrix is an upper bound on the numerical rank. The maximum transversal algorithm in the BTF package is useful in determining if a matrix is structurally rank deficient, and if so, it reveals a (non-unique) set of rows and columns that contribute to that rank deficiency. This is useful in determining what parts of a circuit are poorly formulated (such as dangling components).

When ordered and factorized with KLU, very little fill-in occurs in the resulting LU factors, which means that there is little scope for use of the BLAS [11]. Sparse LU factorization methods that use the BLAS (such as SuperLU [10] amd UMFPACK [4, 5]) are slower than KLU when applied to sparse matrices arising in circuit simulation. Both KLU and SuperLU are based on Gilbert and Peierl's left-looking method [14]. SuperLU uses supernodes, but KLU does not; thus the name *KLU* refers to a "Clark Kent" LU factorization algorithm (what SuperLU was before it became Super).

For details of the permutation to block triangular form, left-looking sparse LU factorization, and approximate minimum degree, refer to [6]. Concise versions of these methods can be found in the CSparse package. KLU is also the topic of a Master's thesis by Palamadai Natarajan [21]; a copy of the thesis can be found in the `KLU/Doc` directory. It includes a description of an earlier version of KLU; some of the function names and parameter lists have changed in this version. The descriptions of the methods used still applies to the current version of KLU, however.

KLU appears as *Algorithm 907: KLU, a direct sparse solver for circuit simulation problems*, ACM Transactions on Mathematical Software, vol 37, no 3, 2010.

3 Availability

KLU and its required ordering packages (BTF, COLAMD, AMD, and SuiteSparse_config) are available at
`http://www.suitesparse.com`. In addition, KLU can make use of any user-provided ordering function. One such function is included, which provides KLU with an interface to the ordering methods used in CHOLMOD [3], such as METIS, a nested dissection method [17]. After permutation to block triangular form, circuit matrices have very good node separators, and are thus excellent candidates for nested dissection. The METIS ordering takes much more time to compute than the AMD ordering, but if the ordering is reused many times (typical in circuit simulation) the better-quality ordering can pay off in lower total simulation time.

To use KLU, you must obtain the KLU, BTF, SuiteSparse_config, AMD, and COLAMD packages in the SuiteSparse suite of sparse matrix libraries. See `http://www.suitesparse.com` for each of these packages. They are also all contained within the single `SuiteSparse.zip` or `SuiteSparse.tar.gz` distribution.

4 Using KLU and BTF in MATLAB

KLU has a single MATLAB interface which provides several options for factorizing a matrix and/or using the factors to solve a linear system. The following is a synopsis of its use. For more details, type `help klu` in MATLAB.

```
LU = klu (A)            factorizes R\A(p,q) into L*U+F, returning a struct
x = klu (A,'\',b)       x = A\b
x = klu (b,'/',A)       x = b/A
x = klu (LU,'\',b)      x = A\b, where LU = klu(A)
x = klu (b,'/',LU)      x = b/A, where LU = klu(A)
```

With a single input klu(A) returns a MATLAB struct containing the LU factors. The factorization is in the form L*U + F = R \ A(p,q) where L*U is the LU factorization of just the diagonal blocks of the block triangular form, F is a sparse matrix containing the entries in the off-diagonal blocks, R is a diagonal matrix containing the row scale factors, and p and q are permutation vectors. The LU struct also contains a vector r which describes the block boundaries (the same as the third output parameter of dmperm). The kth block consists of rows and columns r(k) to r(k+1)-1 in the permuted matrix A(p,q) and the factors L and U.

An optional final input argument (klu(A,opts) for example) provides a way of modifying KLU's user-definable parameters, including a partial pivoting tolerance and ordering options. A second output argument ([LU,info] = klu (...)) provides statistics on the factorization.

The BTF package includes three user-callable MATLAB functions which replicate most of features of the MATLAB built-in dmperm function, and provide an additional option which can significantly limit the worst-case time taken by dmperm. For more details, type help btf, help maxtrans, and help strongcomp in MATLAB. Additional information about how these functions work can be found in [6].

```
[p,q,r] = btf (A)          similar to [p,q,r] = dmperm (A)
q = maxtrans (A)           similar to q = dmperm (A')
[p,r] = strongcomp (A)     similar to [p,q,r] = dmperm (A + speye(n))
```

Both btf and maxtrans include a second option input, maxwork, which limits the total work performed in the maximum transversal to maxwork * nnz(A). The worst-case time taken by the algorithm is O (n * nnz(A)), where the matrix A is n-by-n, but this worst-case time is rarely reached in practice.

To use the KLU and BTF functions in MATLAB, you must first compile and install them. In the BTF/MATLAB directory, type btf_install, and then type klu_install in the KLU/MATLAB directory. Alternatively, if you have the entire SuiteSparse, simply run the SuiteSparse_install function in the SuiteSparse directory. To use METIS 4.0.1 with KLU (and CHOLMOD, another part of SuiteSparse) you must first download it from http://glaros.dtc.umn.edu/gkhome/views/metis and place the metis-4.0 directory in the SuiteSparse directory, alongside the KLU and BTF directories.

After running the installation scripts, type pathtool and save your path for future MATLAB sessions. If you cannot save your path because of file permissions, edit your startup.m by adding addpath commands (type doc startup and doc addpath for more information).

5 Using KLU and BTF in a C program

KLU and BTF include the following C-callable functions. Each function is available in two or four versions: with int or long integers, and (for functions that deal with numerical values), with double or complex double values. The long integer is actually a SuiteSparse_long, which is typically a long, defined with a #define statement. It becomes an __int64 on Microsoft Windows 64, however.

The usage of real and complex, and int and SuiteSparse_long, must not be mixed, except that some functions can be used for both real and complex cases.

5.1 KLU Common object

The klu_common object (klu_l_common for the SuiteSparse_long version) contains user-definable parameters and statistics returned from KLU functions. This object appears in every KLU function

as the last parameter. Details are given in the `klu.h` include file, which also appears below in Section 7. Primary parameters and statistics are summarized below. The defaults are chosen specifically for circuit simulation matrices.

- `tol`: partial pivoting tolerance. If the diagonal entry has a magnitude greater than or equal to `tol` times the largest magnitude of entries in the pivot column, then the diagonal entry is chosen. Default value: 0.001.

- `ordering`: which fill-reducing ordering to use: 0 for AMD, 1 for COLAMD, 2 for a user-provided permutation `P` and `Q` (or a natural ordering if `P` and `Q` are NULL), or 3 for the `user_order` function. Default: 0 (AMD).

- `scale`: whether or not the matrix should be scaled. If `scale < 0`, then no scaling is performed and the input matrix is not checked for errors. If `scale >= 0`, the input matrix is check for errors. If `scale=0`, then no scaling is performed. If `scale=1`, then each row of `A` is divided by the sum of the absolute values in that row. If `scale=2`, then each row of `A` is divided by the maximum absolute value in that row. Default: 2.

- `btf`: if nonzero, then BTF is used to permute the input matrix into block upper triangular form. This step is skipped if `Common.btf` is zero. Default: 1.

- `maxwork`: sets an upper limit on the amount of work performed in `btf_maxtrans` to `maxwork*nnz(A)`. If the limit is reached, a partial zero-free diagonal might be found. This has no effect on whether or not the matrix can be factorized, since the matrix can be factorized with no BTF pre-ordering at all. This option provides a tradeoff between the effectiveness of the BTF ordering and the cost to compute it. A partial result can result in fewer, and larger, blocks in the BTF form, resulting to more work required to factorize the matrix. No limit is enforced if `maxwork <= 0`. Default: 0.

- `user_order`: a pointer to a function that can be provided by the application that uses KLU, to redefine the fill-reducing ordering used by KLU for each diagonal block. The `int` and `SuiteSparse_long` prototypes must be as follows:

```
int my_ordering_function (int n, int Ap [ ], int Ai [ ], int Perm [ ], klu_common *Common) ;


SuiteSparse_long my_long_ordering_function (SuiteSparse_long n,
    SuiteSparse_long Ap [ ], SuiteSparse_long Ai [ ],
    SuiteSparse_long Perm [ ], klu_l_common *Common);
```

  The function should return 0 if an error occurred, and either -1 or a positive (nonzero) value if no error occurred. If greater than zero, then the return value is interpreted by KLU as an estimate of the number of nonzeros in L or U (whichever is greater), when the permuted matrix is factorized. Only an estimate is possible, since partial pivoting with row interchanges is performed during numerical factorization. The input matrix is provided to the function in the parameters `n`, `Ap`, and `Ai`, in compressed-column form. The matrix `A` is `n`-by-`n`. The `Ap` array is of size `n+1`; the `j`th column of `A` has row indices `Ai[Ap[j] ... Ap[j+1]-1]`. The `Ai` array is of size `Ap[n]`. The first column pointer `Ap[0]` is zero. The row indices might not appear sorted in each column, but no duplicates will appear.

The output permutation is to be passed back in the Perm array, where Perm[k]=j means that row and column j of A will appear as the kth row and column of the permuted matrix factorized by KLU. The Perm array is already allocated when it is passed to the user function.

The user function may use, and optionally modify, the contents of the klu_common Common object. In particular, prior to calling KLU, the user application can set both Common.user_order and Common.user_data. The latter is a void * pointer that KLU does not use, except to pass to the user ordering function pointed to by Common.user_order. This is a mechanism for passing additional arguments to the user function.

An example user function is provided in the KLU/User directory, which provides an interface to the ordering method in CHOLMOD.

5.2 KLU Symbolic object

KLU performs its sparse LU factorization in two steps. The first is purely symbolic, and does not depend on the numerical values. This analysis returns a klu_symbolic object (klu_l_symbolic in the SuiteSparse_long version). The Symbolic object contains a pre-ordering which combines the block triangular form with the fill-reducing ordering, and an estimate of the number of nonzeros in the factors of each block. Its size is thus modest, only proportional to n, the dimension of A. It can be reused multiple times for the factorization of a sequence of matrices with identical nonzero pattern. Note: a *nonzero* in this sense is an entry present in the data structure of A; such entries may in fact be numerically zero.

5.3 KLU Numeric object

The Numeric object contains the numeric sparse LU factorization, including the final pivot permutations. To solve a linear system, both the Symbolic and Numeric objects are required.

5.4 A sparse matrix in KLU

The input matrix provided to KLU is in sparse compressed-column form, which is the same data structure used internally in MATLAB, except that the version used here allows for the row indices to appear in any ordering, and this version also allows explicit zero entries to appear in the data structure. The same data structure is used in CSparse, which is fully documented in [6]. If you wish to use a simpler input data structure, consider creating a triplet matrix in CSparse (or CXSparse if you use the long and/or complex versions of KLU), and then convert this data structure into a sparse compressed-column data structure, using the CSparse cs_compress and cs_dupl functions. Similar functions are available in CHOLMOD cholmod_triplet_to_sparse.

The matrix is described with four parameters:

- n: an integer scalar. The matrix is n-by-n. Note that KLU only operates on square matrices.
- Ap: an integer array of size n+1. The first entry is Ap[0]=0, and the last entry nz=Ap[n] is equal to the number of entries in the matrix.
- Ai: an integer array of size nz = Ap[n]. The row indices of entries in column j of A are located in Ai [Ap [j] ... Ap [j+1]-1]. The matrix is zero-based; row and column indices are in the range 0 to n-1.

- `Ax`: a `double` array of size `nz` for the real case, or `2*nz` for the complex case. For the complex case, the real and imaginary parts are interleaved, compatible with Fortran and the ANSI C99 Complex data type. KLU does not rely on the ANSI C99 data type, however, for portability reasons. The numerical values in column `j` of a real matrix are located in `Ax [Ap [j] ... Ap [j+1]-1]`. For a complex matrix, they appear in `Ax [2*Ap [j] ... 2*Ap [j+1]-1]`, as real/imaginary pairs (the real part appears first, followed by the imaginary part).

5.5 `klu_defaults`: set default parameters

This function sets the default parameters for KLU and clears the statistics. It may be used for either the real or complex cases. A value of 0 is returned if an error occurs, 1 otherwise. This function **must** be called before any other KLU function can be called.

```
#include "klu.h"
int ok ;
klu_common Common ;
ok = klu_defaults (&Common) ;                                   /* real or complex */
```

```
#include "klu.h"
SuiteSparse_long ok ;
klu_l_common Common ;
ok = klu_l_defaults (&Common) ;                                 /* real or complex */
```

5.6 `klu_analyze`: order and analyze a matrix

The following usage returns a `Symbolic` object that contains the fill-reducing ordering needed to factorize the matrix `A`. A NULL pointer is returned if a failure occurs. The error status for this function, and all others, is returned in `Common.status`. These functions may be used for both real and complex cases. The AMD ordering is used if `Common.ordering = 0`, COLAMD is used if it is 1, the natural ordering is used if it is 2, and the user-provided `Common.user_ordering` is used if it is 3.

```
#include "klu.h"
int n, Ap [n+1], Ai [nz] ;
klu_symbolic *Symbolic ;
klu_common Common ;
Symbolic = klu_analyze (n, Ap, Ai, &Common) ;                   /* real or complex */
```

```
#include "klu.h"
SuiteSparse_long n, Ap [n+1], Ai [nz] ;
klu_l_symbolic *Symbolic ;
klu_l_common Common ;
Symbolic = klu_l_analyze (n, Ap, Ai, &Common) ;                 /* real or complex */
```

5.7 `klu_analyze_given`: order and analyze a matrix

In this routine, the fill-reducing ordering is provided by the user (`Common.ordering` is ignored). Instead, the row permutation `P` and column permutation `Q` are used. These are integer arrays of size `n`. If NULL, a natural ordering is used (so to provide just a column ordering, pass `Q` as non-NULL and `P` as NULL). A NULL pointer is returned if an error occurs. These functions may be used for both real and complex cases.

```
#include "klu.h"
int n, Ap [n+1], Ai [nz], P [n], Q [n] ;
klu_symbolic *Symbolic ;
klu_common Common ;
Symbolic = klu_analyze_given (n, Ap, Ai, P, Q, &Common) ;               /* real or complex */
```

```
#include "klu.h"
SuiteSparse_long n, Ap [n+1], Ai [nz], P [n], Q [n] ;
klu_l_symbolic *Symbolic ;
klu_l_common Common ;
Symbolic = klu_l_analyze_given (n, Ap, Ai, P, Q, &Common) ;             /* real or complex */
```

5.8 `klu_factor`: numerical factorization

The `klu_factor` function factorizes a matrix, using a sparse left-looking method with threshold partial pivoting. The inputs `Ap` and `Ai` must be unchanged from the previous call to `klu_analyze` that created the `Symbolic` object. A NULL pointer is returned if an error occurs.

```
#include "klu.h"
int Ap [n+1], Ai [nz] ;
double Ax [nz], Az [2*nz] ;
klu_symbolic *Symbolic ;
klu_numeric *Numeric ;
klu_common Common ;
Numeric = klu_factor (Ap, Ai, Ax, Symbolic, &Common) ;                          /* real */
Numeric = klu_z_factor (Ap, Ai, Az, Symbolic, &Common) ;                        /* complex */
```

```
#include "klu.h"
SuiteSparse_long Ap [n+1], Ai [nz] ;
double Ax [nz], Az [2*nz] ;
klu_l_symbolic *Symbolic ;
klu_l_numeric *Numeric ;
klu_l_common Common ;
Numeric = klu_l_factor (Ap, Ai, Ax, Symbolic, &Common) ;                        /* real */
Numeric = klu_zl_factor (Ap, Ai, Az, Symbolic, &Common) ;                       /* complex */
```

5.9 `klu_solve`: solve a linear system

Solves the linear system $Ax = b$, using the `Symbolic` and `Numeric` objects. The right-hand side `B` is overwritten with the solution on output. The array `B` is stored in column major order, with a leading dimension of `ldim`, and `nrhs` columns. Thus, the real entry b_{ij} is stored in `B [i+j*ldim]`, where `ldim >= n` must hold. A complex entry b_{ij} is stored in `B [2*(i+j*ldim)]` and `B [2*(i+j*ldim)+1]` (for the real and imaginary parts, respectively). Returns 1 if successful, 0 if an error occurs.

```
#include "klu.h"
int ldim, nrhs, ok ;
double B [ldim*nrhs], Bz [2*ldim*nrhs] ;
klu_symbolic *Symbolic ;
klu_numeric *Numeric ;
klu_common Common ;
ok = klu_solve (Symbolic, Numeric, ldim, nrhs, B, &Common) ;                   /* real */
ok = klu_z_solve (Symbolic, Numeric, ldim, nrhs, Bz, &Common) ;                /* complex */
```

```
#include "klu.h"
SuiteSparse_long ldim, nrhs, ok ;
double B [ldim*nrhs], Bz [2*ldim*nrhs] ;
klu_symbolic *Symbolic ;
klu_numeric *Numeric ;
klu_common Common ;
ok = klu_l_solve (Symbolic, Numeric, ldim, nrhs, B, &Common) ;                    /* real */
ok = klu_zl_solve (Symbolic, Numeric, ldim, nrhs, Bz, &Common) ;                  /* complex */
```

5.10 klu_tsolve: solve a transposed linear system

Solves the linear system $A^T x = b$ or $A^H x = b$. The conj_solve input is 0 for $A^T x = b$, or nonzero for $A^H x = b$. Otherwise, the function is identical to klu_solve.

```
#include "klu.h"
int ldim, nrhs, ok ;
double B [ldim*nrhs], Bz [2*ldim*nrhs] ;
klu_symbolic *Symbolic ;
klu_numeric *Numeric ;
klu_common Common ;
ok = klu_tsolve (Symbolic, Numeric, ldim, nrhs, B, &Common) ;                     /* real */
ok = klu_z_tsolve (Symbolic, Numeric, ldim, nrhs, Bz, conj_solve, &Common) ;      /* complex */
```

```
#include "klu.h"
SuiteSparse_long ldim, nrhs, ok ;
double B [ldim*nrhs], Bz [2*ldim*nrhs] ;
klu_symbolic *Symbolic ;
klu_numeric *Numeric ;
klu_common Common ;
ok = klu_l_tsolve (Symbolic, Numeric, ldim, nrhs, B, &Common) ;                   /* real */
ok = klu_zl_tsolve (Symbolic, Numeric, ldim, nrhs, Bz, conj_solve, &Common) ;     /* complex */
```

5.11 klu_refactor: numerical refactorization

The klu_refactor function takes as input the Numeric object created by klu_factor (or as modified by a previous call to klu_refactor). It factorizes a new matrix with the same nonzero pattern as that given to the call to klu_factor which created it. The same pivot order is used. Since this can lead to numeric instability, the use of klu_rcond, klu_rgrowth, or klu_condest is recommended to check the accuracy of the resulting factorization. The inputs Ap and Ai must be unmodified since the call to klu_factor that first created the Numeric object. This is function is much faster than klu_factor, and requires no dynamic memory allocation.

```
#include "klu.h"
int ok, Ap [n+1], Ai [nz] ;
double Ax [nz], Az [2*nz] ;
klu_symbolic *Symbolic ;
klu_numeric *Numeric ;
klu_common Common ;
ok = klu_refactor (Ap, Ai, Ax, Symbolic, Numeric, &Common) ;                      /* real */
ok = klu_z_refactor (Ap, Ai, Az, Symbolic, Numeric, &Common) ;                    /* complex */
```

```
#include "klu.h"
SuiteSparse_long ok, Ap [n+1], Ai [nz] ;
```

```
double Ax [nz], Az [2*nz] ;
klu_l_symbolic *Symbolic ;
klu_l_numeric *Numeric ;
klu_l_common Common ;
ok = klu_l_refactor (Ap, Ai, Ax, Symbolic, Numeric, &Common) ;          /* real */
ok = klu_zl_refactor (Ap, Ai, Az, Symbolic, Numeric, &Common) ;         /* complex */
```

5.12 klu_free_symbolic: destroy the Symbolic object

It is the user's responsibility to destroy the `Symbolic` object when it is no longer needed, or else a memory leak will occur. It is safe to pass a NULL `Symbolic` pointer. These functions may be used for both real and complex cases.

```
#include "klu.h"
klu_symbolic *Symbolic ;
klu_common Common ;
klu_free_symbolic (&Symbolic, &Common) ;                        /* real or complex */
```

```
#include "klu.h"
klu_l_symbolic *Symbolic ;
klu_l_common Common ;
klu_l_free_symbolic (&Symbolic, &Common) ;                      /* real or complex */
```

5.13 klu_free_numeric: destroy the Numeric object

It is the user's responsibility to destroy the `Numeric` object when it is no longer needed, or else a memory leak will occur. It is safe to pass a NULL `Numeric` pointer.

```
#include "klu.h"
klu_numeric *Numeric ;
klu_common Common ;
klu_free_numeric (&Numeric, &Common) ;                          /* real */
klu_z_free_numeric (&Numeric, &Common) ;                        /* complex */
```

```
#include "klu.h"
klu_l_numeric *Numeric ;
klu_l_common Common ;
klu_l_free_numeric (&Numeric, &Common) ;                        /* real */
klu_zl_free_numeric (&Numeric, &Common) ;                       /* complex */
```

5.14 klu_sort: sort the columns of L and U

The `klu_factor` function creates a `Numeric` object with factors `L` and `U` stored in a compressed-column form (not the same data structure as `A`, but similar). The columns typically contain lists of row indices in unsorted order. This function sorts these indices, for two purposes: (1) to return `L` and `U` to MATLAB, which expects its sparse matrices to have sorted columns, and (2) to slightly improve the performance of subsequent calls to `klu_solve` and `klu_tsolve`. Except within a MATLAB mexFunction (see `KLU/MATLAB/klu_mex.c`, the use of this function is optional.

```
#include "klu.h"
int ok ;
klu_symbolic *Symbolic ;
klu_numeric *Numeric ;
```

```
klu_common Common ;
ok = klu_sort (Symbolic, Numeric, &Common) ;                                    /* real */
ok = klu_z_sort (Symbolic, Numeric, &Common) ;                                  /* complex */
```

```
#include "klu.h"
SuiteSparse_long ok ;
klu_l_symbolic *Symbolic ;
klu_l_numeric *Numeric ;
klu_l_common Common ;
ok = klu_l_sort (Symbolic, Numeric, &Common) ;                                  /* real */
ok = klu_zl_sort (Symbolic, Numeric, &Common) ;                                 /* complex */
```

5.15 klu_flops: determine the flop count

This function determines the number of floating-point operations performed when the matrix was factorized by `klu_factor` or `klu_refactor`. The result is returned in `Common.flops`.

```
#include "klu.h"
int ok ;
klu_symbolic *Symbolic ;
klu_numeric *Numeric ;
klu_common Common ;
ok = klu_flops (Symbolic, Numeric, &Common) ;                                   /* real */
ok = klu_z_flops (Symbolic, Numeric, &Common) ;                                 /* complex */
```

```
#include "klu.h"
SuiteSparse_long ok ;
klu_l_symbolic *Symbolic ;
klu_l_numeric *Numeric ;
klu_l_common Common ;
ok = klu_l_flops (Symbolic, Numeric, &Common) ;                                 /* real */
ok = klu_zl_flops (Symbolic, Numeric, &Common) ;                                /* complex */
```

5.16 klu_rgrowth: determine the pivot growth

Computes the reciprocal pivot growth, $rgrowth = \min_j ((\max_i |c_{ij}|)/(\max_i |u_{ij}|))$, where c_{ij} is a scaled entry in a diagonal block of the block triangular form. In MATLAB notation:

```
rgrowth = min (max (abs (R\A(p,q) - F)) ./ max (abs (U)))
```

where the factorization is `L*U + F = R \ A(p,q)`. This function returns 0 if an error occurred, 1 otherwise. If `rgrowth` is very small, an inaccurate factorization may have been performed. The inputs `Ap`, `Ai`, and `Ax` (`Az` in the complex case) must be unchanged since the last call to `klu_factor` or `klu_refactor`. The result is returned in `Common.rgrowth`.

```
#include "klu.h"
int ok, Ap [n+1], Ai [nz] ;
double Ax [nz], Az [2*nz] ;
klu_symbolic *Symbolic ;
klu_numeric *Numeric ;
klu_common Common ;
ok = klu_rgrowth (Ap, Ai, Ax, Symbolic, Numeric, &Common) ;                     /* real */
ok = klu_z_rgrowth (Ap, Ai, Az, Symbolic, Numeric, &Common) ;                   /* complex */
```

```
#include "klu.h"
SuiteSparse_long ok, Ap [n+1], Ai [nz] ;
double Ax [nz], Az [2*nz] ;
klu_l_symbolic *Symbolic ;
klu_l_numeric *Numeric ;
klu_l_common Common ;
ok = klu_l_rgrowth (Ap, Ai, Ax, Symbolic, Numeric, &Common) ;                    /* real */
ok = klu_zl_rgrowth (Ap, Ai, Az, Symbolic, Numeric, &Common) ;                   /* complex */
```

5.17 klu_condest: accurate condition number estimation

This function is essentially the same as the MATLAB condest function. It computes an estimate of the 1-norm condition number, using Hager's method [15] and the generalization by Higham and Tisseur [16]. The inputs Ap, and Ax (Az in the complex case) must be unchanged since the last call to klu_factor or klu_refactor. The result is returned in Common.condest.

```
#include "klu.h"
int ok, Ap [n+1] ;
double Ax [nz], Az [2*nz] ;
klu_symbolic *Symbolic ;
klu_numeric *Numeric ;
klu_common Common ;
ok = klu_condest (Ap, Ax, Symbolic, Numeric, &Common) ;                          /* real */
ok = klu_z_condest (Ap, Az, Symbolic, Numeric, &Common) ;                        /* complex */
```

```
#include "klu.h"
SuiteSparse_long ok, Ap [n+1] ;
double Ax [nz], Az [2*nz] ;
klu_l_symbolic *Symbolic ;
klu_l_numeric *Numeric ;
klu_l_common Common ;
ok = klu_l_condest (Ap, Ax, Symbolic, Numeric, &Common) ;                        /* real */
ok = klu_zl_condest (Ap, Az, Symbolic, Numeric, &Common) ;                       /* complex */
```

5.18 klu_rcond: cheap reciprocal condition number estimation

This function returns the smallest diagonal entry of U divided by the largest, which is a very crude estimate of the reciprocal of the condition number of the matrix A. It is very cheap to compute, however. In MATLAB notation, rcond = min(abs(diag(U))) / max(abs(diag(U))). If the matrix is singular, rcond will be zero. The result is returned in Common.rcond.

```
#include "klu.h"
int ok ;
klu_symbolic *Symbolic ;
klu_numeric *Numeric ;
klu_common Common ;
ok = klu_rcond (Symbolic, Numeric, &Common) ;                                    /* real */
ok = klu_z_rcond (Symbolic, Numeric, &Common) ;                                  /* complex */
```

```
#include "klu.h"
SuiteSparse_long ok ;
klu_l_symbolic *Symbolic ;
klu_l_numeric *Numeric ;
klu_l_common Common ;
```

```
ok = klu_l_rcond (Symbolic, Numeric, &Common) ;                              /* real */
ok = klu_zl_rcond (Symbolic, Numeric, &Common) ;                             /* complex */
```

5.19 klu_scale: scale and check a sparse matrix

This function computes the row scaling factors of a matrix and checks to see if it is a valid sparse matrix. It can perform two kinds of scaling, computing either the largest magnitude in each row, or the sum of the magnitudes of the entries each row. KLU calls this function itself, depending upon the `Common.scale` parameter, where `scale < 0` means no scaling, `scale=1` means the sum, and `scale=2` means the maximum. That is, in MATLAB notation, `Rs = sum(abs(A'))` or `Rs = max(abs(A'))`. KLU then divides each row of `A` by its corresponding scale factor. The function returns 0 if the matrix is invalid, or 1 otherwise. A valid sparse matrix must meet the following conditions:

1. `n > 0`. Note that KLU does not handle empty (0-by-0) matrices.
2. `Ap`, `Ai`, and `Ax` (`Az` for the complex case) must not be NULL.
3. `Ap[0]=0`, and `Ap [j] <= Ap [j+1]` for all `j` in the range 0 to `n-1`.
4. The row indices in each column, `Ai [Ap [j] ... Ap [j+1]-1]`, must be in the range 0 to `n-1`, and no duplicates can appear. If the workspace `W` is NULL on input, the check for duplicate entries is skipped.

```
#include "klu.h"
int scale, ok, n, Ap [n+1], Ai [nz], W [n] ;
double Ax [nz], Az [2*nz], Rs [n] ;
klu_common Common ;
ok = klu_scale (scale, n, Ap, Ai, Ax, Symbolic, Numeric, &Common) ;          /* real */
ok = klu_z_scale (scale, n, Ap, Ai, Az, Symbolic, Numeric, &Common) ;        /* complex */
```

```
#include "klu.h"
SuiteSparse_long scale, ok, n, Ap [n+1], Ai [nz], W [n] ;
double Ax [nz], Az [2*nz], Rs [n] ;
klu_l_common Common ;
ok = klu_l_scale (scale, n, Ap, Ai, Ax, Symbolic, Numeric, &Common) ;        /* real */
ok = klu_zl_scale (scale, n, Ap, Ai, Az, Symbolic, Numeric, &Common) ;       /* complex */
```

5.20 klu_extract: extract the LU factorization

This function extracts the LU factorization into a set of data structures suitable for passing back to MATLAB, with matrices in conventional compressed-column form. The `klu_sort` function should be called first if the row indices should be returned sorted. The factorization is returned in caller-provided arrays; if any of them are NULL, that part of the factorization is not extracted (this is not an error). Returns 1 if successful, 0 otherwise.

The sizes of `Li`, `Lx`, and `Lz` are `Numeric->lnz`, `Ui`, `Ux`, and `Uz` are of size `Numeric->unz`, and `Fi`, `Fx`, and `Fz` are of size `Numeric->nzoff`. Note that in the complex versions, the real and imaginary parts are returned in separate arrays, to be compatible with how MATLAB stores complex matrices.

This function is not required to solve a linear system with KLU. KLU does not itself make use of the extracted LU factorization returned by this function. It is only provided to simplify the MATLAB interface to KLU, and it may be of use to the end user who wishes to examine the contents of the LU factors.

```
#include "klu.h"
int ok, Lp [n+1], Li [lnz], Up [n+1], Ui [unz], Fp [n+1], Fi [nzoff], P [n], Q [n], R [n] ;
double Lx [lnz], Lz [lnz], Ux [unz], Uz [unz], Fx [nzoff], Fz [nzoff], Rs [n] ;
klu_symbolic *Symbolic ;
klu_numeric *Numeric ;
klu_common Common ;
ok = klu_extract (Numeric, Symbolic,
    Lp, Li, Lx, Up, Ui, Ux, Fp, Fi, Fx, P, Q, Rs, R, &Common) ;                   /* real */
ok = klu_z_extract (Numeric, Symbolic,
    Lp, Li, Lx, Lz, Up, Ui, Ux, Uz, Fp, Fi, Fx, Fz, P, Q, Rs, R, &Common) ;       /* complex */
```

```
#include "klu.h"
SuiteSparse_long ok, Lp [n+1], Li [lnz], Up [n+1], Ui [unz], Fp [n+1],
    Fi [nzoff], P [n], Q [n], R [n] ;
double Lx [lnz], Lz [lnz], Ux [unz], Uz [unz], Fx [nzoff], Fz [nzoff], Rs [n] ;
klu_l_symbolic *Symbolic ;
klu_l_numeric *Numeric ;
klu_l_common Common ;
ok = klu_l_extract (Numeric, Symbolic,
    Lp, Li, Lx, Up, Ui, Ux, Fp, Fi, Fx, P, Q, Rs, R, &Common) ;                   /* real */
ok = klu_zl_extract (Numeric, Symbolic,
    Lp, Li, Lx, Lz, Up, Ui, Ux, Uz, Fp, Fi, Fx, Fz, P, Q, Rs, R, &Common) ;       /* complex */
```

5.21 `klu_malloc`, `klu_free`, `klu_realloc`: memory management

KLU uses a set of wrapper routines for `malloc`, `free`, and `realloc`. By default, these wrapper routines call the ANSI C versions of these functions. However, pointers to functions in `Common` can be modified after calling `klu_defaults` to allow the use of other memory management functions (such as the MATLAB `mxMalloc`, `mxFree`, and `mxRealloc`. These wrapper functions keep track of the current and peak memory usage of KLU. They can be called by the user.

`klu_malloc` is essentially the same as `p = malloc (n * sizeof (size))`, `klu_free` is essentially the same as `free(p)` except that `klu_free` returns NULL which can then be assigned to `p`. `klu_realloc` is similar to `realloc`, except that if the reallocation fails, `p` is returned unchanged. Failure conditions are returned in `Common.status`.

```
#include "klu.h"
size_t n, nnew, nold, size ;
void *p ;
klu_common Common ;
p = klu_malloc (n, size, &Common) ;
p = klu_free (p, n, size, &Common) ;
p = klu_realloc (nnew, nold, size, p, &Common) ;
```

```
#include "klu.h"
size_t n, nnew, nold, size ;
void *p ;
klu_l_common Common ;
p = klu_l_malloc (n, size, &Common) ;
p = klu_l_free (p, n, size, &Common) ;
p = klu_l_realloc (nnew, nold, size, p, &Common) ;
```

5.22 btf_maxtrans: maximum transversal

The BTF package includes three user-callable functions (each with int and SuiteSparse_long versions). They do not need to be called directly by an application that uses KLU. KLU will call these functions to perform the permutation into upper block triangular form.

The btf_maxtrans function finds a column permutation Q that gives A*Q a zero-free diagonal, if one exists. If row i is matched to column j, then Match[i]=j. If the matrix is structurally singular, there will be some unmatched rows. If row i is unmatched, then Match[i]=-1. If the matrix is square and structurally non-singular, then Q=Match is the column permutation. The btf_maxtrans function can accept as input a rectangular matrix; it operates on the bipartite graph of A. It returns the number of columns matched. Unlike the KLU user-callable functions, the BTF functions do not check its inputs at all; a segmentation fault will occur if any input pointers are NULL, for example.

The function can require up to $O($n*nnz(A)$)$ time (excluding the *cheap match* phase, which takes another $O($nnz(A)$)$ time. If maxwork > 0 on input, the work is limited to $O($maxwork*nnz(A)$)$ (excluding the cheap match), but the maximum transversal might not be found if the limit is reached.

The Work array is workspace required by the methods; its contents are undefined on input and output.

```
int nrow, ncol, Ap [ncol+1], Ai [nz], Match [nrow], Work [5*ncol], nmatch ;
double maxwork, work ;
nmatch = btf_maxtrans (nrow, ncol, Ap, Ai, maxwork, &work, Match, Work) ;
```

```
SuiteSparse_long nrow, ncol, Ap [ncol+1], Ai [nz], Match [nrow], Work [5*ncol], nmatch ;
double maxwork, work ;
nmatch = btf_l_maxtrans (nrow, ncol, Ap, Ai, maxwork, &work, Match, Work) ;
```

5.23 btf_strongcomp: strongly connected components

The btf_strongcomp function finds the strongly connected components of a directed graph, returning a symmetric permutation P. The matrix A must be square. The diagonal of A (or A*Q if a column permutation is given on input) is ignored. If Q is NULL on input, the matrix P*A*P' is in upper block triangular form. Otherwise, Q is modified on output so that P*A*Q is in upper block triangular form. The vector R gives the block boundaries, where the kth block consists of rows and columns R[k] through R[k+1]-1 in the permuted matrix. The function returns the number of strongly connected components found (the diagonal blocks in the block triangular form).

```
int n, Ap [n+1], Ai [nz], Q [n], P [n], R [n+1], Work [4*n], ncomp ;
ncomp = btf_strongcomp (n, Ap, Ai, Q, P, R, Work) ;
```

```
SuiteSparse_long n, Ap [n+1], Ai [nz], Q [n], P [n], R [n+1], Work [4*n], ncomp ;
ncomp = btf_l_strongcomp (n, Ap, Ai, Q, P, R, Work) ;
```

5.24 btf_order: permutation to block triangular form

The btf_order function combines the above two functions, first finding a maximum transversal and then permuting the resulting matrix into upper block triangular form. Unlike dmperm in MATLAB, it always reveals the maximum matching along the diagonal, even if the matrix is structurally singular.

On output, P and Q are the row and column permutations, where i = P[k] if row i of A is the kth row of P*A*Q, and j = BTF_UNFLIP(Q[k]) if column j of A is the kth column of P*A*Q. If Q[k] < 0, then the (k,k)th entry in P*A*Q is structurally zero. The vector R, and the return value, are the same as btf_strongcomp.

```
int n, Ap [n+1], Ai [nz], P [n], Q [n], R [n+1], nfound, Work [5*n], ncomp, nfound ;
double maxwork, work ;
ncomp = btf_order (n, Ap, Ai, maxwork, &work, P, Q, R, &nfound, Work) ;


SuiteSparse_long n, Ap [n+1], Ai [nz], P [n], Q [n], R [n+1], nfound, Work [5*n], ncomp, nfound ;
double maxwork, work ;
ncomp = btf_l_order (n, Ap, Ai, maxwork, &work, P, Q, R, &nfound, Work) ;
```

5.25 Sample C programs that use KLU

Here is a simple main program, klu_simple.c, that illustrates the basic usage of KLU. It uses KLU, and indirectly makes use of BTF and AMD. COLAMD is required to compile the demo, but it is not called by this example. It uses statically defined global variables for the sparse matrix A, which would not be typical of a complete application. It just makes for a simpler example.

```
/* klu_simple: a simple KLU demo; solution is x = (1,2,3,4,5) */

#include <stdio.h>
#include "klu.h"

int    n = 5 ;
int    Ap [ ] = {0, 2, 5, 9, 10, 12} ;
int    Ai [ ] = { 0,  1,  0,   2,  4,  1,  2,  3,   4,  2,  1,  4} ;
double Ax [ ] = {2., 3., 3., -1., 4., 4., -3., 1., 2., 2., 6., 1.} ;
double b [ ] = {8., 45., -3., 3., 19.} ;

int main (void)
{
    klu_symbolic *Symbolic ;
    klu_numeric *Numeric ;
    klu_common Common ;
    int i ;
    klu_defaults (&Common) ;
    Symbolic = klu_analyze (n, Ap, Ai, &Common) ;
    Numeric = klu_factor (Ap, Ai, Ax, Symbolic, &Common) ;
    klu_solve (Symbolic, Numeric, 5, 1, b, &Common) ;
    klu_free_symbolic (&Symbolic, &Common) ;
    klu_free_numeric (&Numeric, &Common) ;
    for (i = 0 ; i < n ; i++) printf ("x [%d] = %g\n", i, b [i]) ;
    return (0) ;
}
```

The Ap, Ai, and Ax arrays represent the matrix

$$A = \begin{bmatrix} 2 & 3 & 0 & 0 & 0 \\ 3 & 0 & 4 & 0 & 6 \\ 0 & -1 & -3 & 2 & 0 \\ 0 & 0 & 1 & 0 & 0 \\ 0 & 4 & 2 & 0 & 1 \end{bmatrix}.$$

The solution to $Ax = b$ is $x = [1\,2\,3\,4\,5]^T$. The program uses default control settings (no scaling, permutation to block triangular form, and the AMD ordering). It ignores the error codes in the return values and `Common.status`.

The block triangular form found by `btf_order` for this matrix is given below

$$PAQ = \left[\begin{array}{c|ccc|c} 2 & 0 & 0 & -1 & -3 \\ \hline & 2 & 0 & 3 & 0 \\ & 3 & 6 & 0 & 4 \\ & 0 & 1 & 4 & 1 \\ \hline & & & & 1 \end{array}\right].$$

This ordering is not modified by the AMD ordering because the 3-by-3 matrix $A_{22} + A_{22}^T$ happens to be a dense matrix. No partial pivoting happens to occur during LU factorization; all pivots are selected along the diagonal of each block. The matrix contains two singletons, which are the original entries $a_{34} = 2$ and $a_{43} = 1$, and one 3-by-3 diagonal block (in which a single fill-in entry occurs during factorization: the u_{23} entry of this 3-by-3 matrix).

For a more complete program that uses KLU, see `KLU/Demo/kludemo.c` for an `int` version, and `KLU/Demo/kluldemo.c` for a version that uses `SuiteSparse_long` instead. The top-level main routine uses CHOLMOD to read in a compressed-column sparse matrix from a Matrix Market file, because KLU does not include such a function. Otherwise, no CHOLMOD functions are used. Unlike `klu_simple.c`, CHOLMOD is required to run the `kludemo.c` and `kluldemo.c` programs.

6 Installation

Installation of the C-callable interface requires the `make` utility, in Linux/Unix. Alternatively, you can use the Cygwin `make` in Windows. The MATLAB installation in any platform, including Windows is simple; just type `klu_install` to compile and install KLU, BTF, AMD, and COLAMD.

For `make`, system-dependent configurations are in the `../SuiteSparse_config/SuiteSparse_config.mk` file. You can edit that file to customize the compilation. The default settings will work on most systems. Sample configuration files are provided for Linux, Sun Solaris, SGI IRIX, IBM AIX, and the DEC/Compaq Alpha.

To compile and install the C-callable KLU, BTF, AMD, and COLAMD libraries, go to the `KLU` directory and type `make`. The KLU and BTF libraries are placed in `KLU/Lib/libklu.a` and `BTF/Lib/libbtf.a`. Two KLU demo programs will be compiled and tested in the `KLU/Demo` directory. You can compare the output of `make` with the results in the KLU distribution, `kludemo.out`.

Typing `make clean` will remove all but the final compiled libraries and demo programs. Typing `make purge` or `make distclean` removes all files not in the original distribution. If you compile KLU or BTF and then later change the `../SuiteSparse_config/SuiteSparse_config.mk` file then you should type `make purge` and then `make` to recompile.

When you compile your program that uses the C-callable KLU library, you need to add the `KLU/Lib/libklu.a`, `BTF/Lib/libbtf.a`, `AMD/Lib/libamd.a`, and `COLAMD/Lib/libamd.a` libraries, a nd you need to tell your compiler to look in the `KLU/Include` and `BTF/Include` directory for include files.

If all you want to use is the KLU mexFunction in MATLAB, you can skip the use of the `make` command entirely. Simply type `klu_install` in the MATLAB command window while in the `KLU/MATLAB` directory. This works on any system with MATLAB, including Windows. Alternately, type `make` in the `KLU/MATLAB` directory.

7 The KLU routines

The file KLU/Include/klu.h listed below describes each user-callable routine in the C version of KLU, and gives details on their use.

```
/* ========================================================================== */
/* === klu include file ===================================================== */
/* ========================================================================== */

/* Include file for user programs that call klu_* routines */

#ifndef _KLU_H
#define _KLU_H

/* make it easy for C++ programs to include KLU */
#ifdef __cplusplus
extern "C" {
#endif

#include "amd.h"
#include "colamd.h"
#include "btf.h"

/* -------------------------------------------------------------------------- */
/* Symbolic object - contains the pre-ordering computed by klu_analyze */
/* -------------------------------------------------------------------------- */

typedef struct
{
    /* A (P,Q) is in upper block triangular form.  The kth block goes from
     * row/col index R [k] to R [k+1]-1.  The estimated number of nonzeros
     * in the L factor of the kth block is Lnz [k].
     */

    /* only computed if the AMD ordering is chosen: */
    double symmetry ;   /* symmetry of largest block */
    double est_flops ;  /* est. factorization flop count */
    double lnz, unz ;   /* estimated nz in L and U, including diagonals */
    double *Lnz ;       /* size n, but only Lnz [0..nblocks-1] is used */

    /* computed for all orderings: */
    int
        n,              /* input matrix A is n-by-n */
        nz,             /* # entries in input matrix */
        *P,             /* size n */
        *Q,             /* size n */
        *R,             /* size n+1, but only R [0..nblocks] is used */
        nzoff,          /* nz in off-diagonal blocks */
        nblocks,        /* number of blocks */
        maxblock,       /* size of largest block */
        ordering,       /* ordering used (AMD, COLAMD, or GIVEN) */
        do_btf ;        /* whether or not BTF preordering was requested */

    /* only computed if BTF preordering requested */
    int structural_rank ;   /* 0 to n-1 if the matrix is structurally rank
                        * deficient.  -1 if not computed.  n if the matrix has
                        * full structural rank */

} klu_symbolic ;
```

```
typedef struct          /* 64-bit version (otherwise same as above) */
{
    double symmetry, est_flops, lnz, unz ;
    double *Lnz ;
    SuiteSparse_long n, nz, *P, *Q, *R, nzoff, nblocks, maxblock, ordering,
        do_btf, structural_rank ;

} klu_l_symbolic ;

/* -------------------------------------------------------------------------- */
/* Numeric object - contains the factors computed by klu_factor */
/* -------------------------------------------------------------------------- */

typedef struct
{
    /* LU factors of each block, the pivot row permutation, and the
     * entries in the off-diagonal blocks */

    int n ;             /* A is n-by-n */
    int nblocks ;       /* number of diagonal blocks */
    int lnz ;           /* actual nz in L, including diagonal */
    int unz ;           /* actual nz in U, including diagonal */
    int max_lnz_block ; /* max actual nz in L in any one block, incl. diag */
    int max_unz_block ; /* max actual nz in U in any one block, incl. diag */
    int *Pnum ;         /* size n. final pivot permutation */
    int *Pinv ;         /* size n. inverse of final pivot permutation */

    /* LU factors of each block */
    int *Lip ;          /* size n. pointers into LUbx[block] for L */
    int *Uip ;          /* size n. pointers into LUbx[block] for U */
    int *Llen ;         /* size n. Llen [k] = # of entries in kth column of L */
    int *Ulen ;         /* size n. Ulen [k] = # of entries in kth column of U */
    void **LUbx ;       /* L and U indices and entries (excl. diagonal of U) */
    size_t *LUsize ;    /* size of each LUbx [block], in sizeof (Unit) */
    void *Udiag ;       /* diagonal of U */

    /* scale factors; can be NULL if no scaling */
    double *Rs ;        /* size n. Rs [i] is scale factor for row i */

    /* permanent workspace for factorization and solve */
    size_t worksize ;   /* size (in bytes) of Work */
    void *Work ;        /* workspace */
    void *Xwork ;       /* alias into Numeric->Work */
    int *Iwork ;        /* alias into Numeric->Work */

    /* off-diagonal entries in a conventional compressed-column sparse matrix */
    int *Offp ;         /* size n+1, column pointers */
    int *Offi ;         /* size nzoff, row indices */
    void *Offx ;        /* size nzoff, numerical values */
    int nzoff ;

} klu_numeric ;

typedef struct          /* 64-bit version (otherwise same as above) */
{
    SuiteSparse_long n, nblocks, lnz, unz, max_lnz_block, max_unz_block, *Pnum,
        *Pinv, *Lip, *Uip, *Llen, *Ulen ;
    void **LUbx ;
```

```
    size_t *LUsize ;
    void *Udiag ;
    double *Rs ;
    size_t worksize ;
    void *Work, *Xwork ;
    SuiteSparse_long *Iwork ;
    SuiteSparse_long *Offp, *Offi ;
    void *Offx ;
    SuiteSparse_long nzoff ;

} klu_l_numeric ;

/* -------------------------------------------------------------------------- */
/* KLU control parameters and statistics */
/* -------------------------------------------------------------------------- */

/* Common->status values */
#define KLU_OK 0
#define KLU_SINGULAR (1)            /* status > 0 is a warning, not an error */
#define KLU_OUT_OF_MEMORY (-2)
#define KLU_INVALID (-3)
#define KLU_TOO_LARGE (-4)          /* integer overflow has occured */

typedef struct klu_common_struct
{

    /* ---------------------------------------------------------------------- */
    /* parameters */
    /* ---------------------------------------------------------------------- */

    double tol ;            /* pivot tolerance for diagonal preference */
    double memgrow ;        /* realloc memory growth size for LU factors */
    double initmem_amd ;    /* init. memory size with AMD: c*nnz(L) + n */
    double initmem ;        /* init. memory size: c*nnz(A) + n */
    double maxwork ;        /* maxwork for BTF, <= 0 if no limit */

    int btf ;               /* use BTF pre-ordering, or not */
    int ordering ;          /* 0: AMD, 1: COLAMD, 2: user P and Q,
                             * 3: user function */
    int scale ;             /* row scaling: -1: none (and no error check),
                             * 0: none, 1: sum, 2: max */

    /* pointer to user ordering function */
    int (*user_order) (int, int *, int *, int *, struct klu_common_struct *) ;

    /* pointer to user data, passed unchanged as the last parameter to the
     * user ordering function (optional, the user function need not use this
     * information). */
    void *user_data ;

    int halt_if_singular ;      /* how to handle a singular matrix:
        * FALSE: keep going.  Return a Numeric object with a zero U(k,k).  A
        *   divide-by-zero may occur when computing L(:,k).  The Numeric object
        *   can be passed to klu_solve (a divide-by-zero will occur).  It can
        *   also be safely passed to klu_refactor.
        * TRUE: stop quickly.  klu_factor will free the partially-constructed
        *   Numeric object.  klu_refactor will not free it, but will leave the
        *   numerical values only partially defined.  This is the default. */
```

```
    /* ---------------------------------------------------------------------- */
    /* statistics */
    /* ---------------------------------------------------------------------- */

    int status ;                /* KLU_OK if OK, < 0 if error */
    int nrealloc ;              /* # of reallocations of L and U */

    int structural_rank ;       /* 0 to n-1 if the matrix is structurally rank
        * deficient (as determined by maxtrans).  -1 if not computed.  n if the
        * matrix has full structural rank.  This is computed by klu_analyze
        * if a BTF preordering is requested. */

    int numerical_rank ;        /* First k for which a zero U(k,k) was found,
        * if the matrix was singular (in the range 0 to n-1).  n if the matrix
        * has full rank. This is not a true rank-estimation.  It just reports
        * where the first zero pivot was found.  -1 if not computed.
        * Computed by klu_factor and klu_refactor. */

    int singular_col ;          /* n if the matrix is not singular.  If in the
        * range 0 to n-1, this is the column index of the original matrix A that
        * corresponds to the column of U that contains a zero diagonal entry.
        * -1 if not computed.  Computed by klu_factor and klu_refactor. */

    int noffdiag ;      /* # of off-diagonal pivots, -1 if not computed */

    double flops ;      /* actual factorization flop count, from klu_flops */
    double rcond ;      /* crude reciprocal condition est., from klu_rcond */
    double condest ;    /* accurate condition est., from klu_condest */
    double rgrowth ;    /* reciprocal pivot rgrowth, from klu_rgrowth */
    double work ;       /* actual work done in BTF, in klu_analyze */

    size_t memusage ;   /* current memory usage, in bytes */
    size_t mempeak ;    /* peak memory usage, in bytes */

} klu_common ;

typedef struct klu_l_common_struct /* 64-bit version (otherwise same as above)*/
{

    double tol, memgrow, initmem_amd, initmem, maxwork ;
    SuiteSparse_long btf, ordering, scale ;
    SuiteSparse_long (*user_order) (SuiteSparse_long, SuiteSparse_long *,
        SuiteSparse_long *, SuiteSparse_long *,
        struct klu_l_common_struct *) ;
    void *user_data ;
    SuiteSparse_long halt_if_singular ;
    SuiteSparse_long status, nrealloc, structural_rank, numerical_rank,
        singular_col, noffdiag ;
    double flops, rcond, condest, rgrowth, work ;
    size_t memusage, mempeak ;

} klu_l_common ;

/* -------------------------------------------------------------------------- */
/* klu_defaults: sets default control parameters */
/* -------------------------------------------------------------------------- */

int klu_defaults
(
```

```
    klu_common *Common
) ;

SuiteSparse_long klu_l_defaults (klu_l_common *Common) ;

/* -------------------------------------------------------------------------- */
/* klu_analyze:  orders and analyzes a matrix */
/* -------------------------------------------------------------------------- */

/* Order the matrix with BTF (or not), then order each block with AMD, COLAMD,
 * a natural ordering, or with a user-provided ordering function */

klu_symbolic *klu_analyze
(
    /* inputs, not modified */
    int n,              /* A is n-by-n */
    int Ap [ ],         /* size n+1, column pointers */
    int Ai [ ],         /* size nz, row indices */
    klu_common *Common
) ;

klu_l_symbolic *klu_l_analyze (SuiteSparse_long, SuiteSparse_long *,
    SuiteSparse_long *, klu_l_common *Common) ;


/* -------------------------------------------------------------------------- */
/* klu_analyze_given: analyzes a matrix using given P and Q */
/* -------------------------------------------------------------------------- */

/* Order the matrix with BTF (or not), then use natural or given ordering
 * P and Q on the blocks.  P and Q are interpretted as identity
 * if NULL. */

klu_symbolic *klu_analyze_given
(
    /* inputs, not modified */
    int n,              /* A is n-by-n */
    int Ap [ ],         /* size n+1, column pointers */
    int Ai [ ],         /* size nz, row indices */
    int P [ ],          /* size n, user's row permutation (may be NULL) */
    int Q [ ],          /* size n, user's column permutation (may be NULL) */
    klu_common *Common
) ;

klu_l_symbolic *klu_l_analyze_given (SuiteSparse_long, SuiteSparse_long *,
    SuiteSparse_long *, SuiteSparse_long *, SuiteSparse_long *,
    klu_l_common *) ;


/* -------------------------------------------------------------------------- */
/* klu_factor:  factors a matrix using the klu_analyze results */
/* -------------------------------------------------------------------------- */

klu_numeric *klu_factor /* returns KLU_OK if OK, < 0 if error */
(
    /* inputs, not modified */
    int Ap [ ],         /* size n+1, column pointers */
    int Ai [ ],         /* size nz, row indices */
    double Ax [ ],      /* size nz, numerical values */
```

```
    klu_symbolic *Symbolic,
    klu_common *Common
) ;

klu_numeric *klu_z_factor      /* returns KLU_OK if OK, < 0 if error */
(
     /* inputs, not modified */
     int Ap [ ],        /* size n+1, column pointers */
     int Ai [ ],        /* size nz, row indices */
     double Ax [ ],     /* size 2*nz, numerical values (real,imag pairs) */
     klu_symbolic *Symbolic,
     klu_common *Common
) ;

/* long / real version */
klu_l_numeric *klu_l_factor (SuiteSparse_long *, SuiteSparse_long *, double *,
    klu_l_symbolic *, klu_l_common *) ;

/* long / complex version */
klu_l_numeric *klu_zl_factor (SuiteSparse_long *, SuiteSparse_long *, double *,
    klu_l_symbolic *, klu_l_common *) ;


/* -------------------------------------------------------------------------- */
/* klu_solve: solves Ax=b using the Symbolic and Numeric objects */
/* -------------------------------------------------------------------------- */

int klu_solve
(
    /* inputs, not modified */
    klu_symbolic *Symbolic,
    klu_numeric *Numeric,
    int ldim,               /* leading dimension of B */
    int nrhs,               /* number of right-hand-sides */

    /* right-hand-side on input, overwritten with solution to Ax=b on output */
    double B [ ],           /* size ldim*nrhs */
    klu_common *Common
) ;

int klu_z_solve
(
     /* inputs, not modified */
     klu_symbolic *Symbolic,
     klu_numeric *Numeric,
     int ldim,               /* leading dimension of B */
     int nrhs,               /* number of right-hand-sides */

     /* right-hand-side on input, overwritten with solution to Ax=b on output */
     double B [ ],          /* size 2*ldim*nrhs */
     klu_common *Common
) ;

SuiteSparse_long klu_l_solve (klu_l_symbolic *, klu_l_numeric *,
    SuiteSparse_long, SuiteSparse_long, double *, klu_l_common *) ;

SuiteSparse_long klu_zl_solve (klu_l_symbolic *, klu_l_numeric *,
    SuiteSparse_long, SuiteSparse_long, double *, klu_l_common *) ;
```

```
/* -------------------------------------------------------------------------- */
/* klu_tsolve: solves A'x=b using the Symbolic and Numeric objects */
/* -------------------------------------------------------------------------- */

int klu_tsolve
(
    /* inputs, not modified */
    klu_symbolic *Symbolic,
    klu_numeric *Numeric,
    int ldim,               /* leading dimension of B */
    int nrhs,               /* number of right-hand-sides */

    /* right-hand-side on input, overwritten with solution to Ax=b on output */
    double B [ ],           /* size ldim*nrhs */
    klu_common *Common
) ;

int klu_z_tsolve
(
    /* inputs, not modified */
    klu_symbolic *Symbolic,
    klu_numeric *Numeric,
    int ldim,               /* leading dimension of B */
    int nrhs,               /* number of right-hand-sides */

    /* right-hand-side on input, overwritten with solution to Ax=b on output */
    double B [ ],           /* size 2*ldim*nrhs */
    int conj_solve,         /* TRUE: conjugate solve, FALSE: solve A.'x=b */
    klu_common *Common

) ;

SuiteSparse_long klu_l_tsolve (klu_l_symbolic *, klu_l_numeric *,
    SuiteSparse_long, SuiteSparse_long, double *, klu_l_common *) ;

SuiteSparse_long klu_zl_tsolve (klu_l_symbolic *, klu_l_numeric *,
    SuiteSparse_long, SuiteSparse_long, double *, SuiteSparse_long,
    klu_l_common * ) ;


/* -------------------------------------------------------------------------- */
/* klu_refactor: refactorizes matrix with same ordering as klu_factor */
/* -------------------------------------------------------------------------- */

int klu_refactor            /* return TRUE if successful, FALSE otherwise */
(
    /* inputs, not modified */
    int Ap [ ],         /* size n+1, column pointers */
    int Ai [ ],         /* size nz, row indices */
    double Ax [ ],      /* size nz, numerical values */
    klu_symbolic *Symbolic,
    /* input, and numerical values modified on output */
    klu_numeric *Numeric,
    klu_common *Common
) ;

int klu_z_refactor          /* return TRUE if successful, FALSE otherwise */
(
```

```
     /* inputs, not modified */
     int Ap [ ],        /* size n+1, column pointers */
     int Ai [ ],        /* size nz, row indices */
     double Ax [ ],     /* size 2*nz, numerical values */
     klu_symbolic *Symbolic,
     /* input, and numerical values modified on output */
     klu_numeric *Numeric,
     klu_common *Common
) ;

SuiteSparse_long klu_l_refactor (SuiteSparse_long *, SuiteSparse_long *,
    double *, klu_l_symbolic *, klu_l_numeric *, klu_l_common *) ;

SuiteSparse_long klu_zl_refactor (SuiteSparse_long *, SuiteSparse_long *,
    double *, klu_l_symbolic *, klu_l_numeric *, klu_l_common *) ;


/* -------------------------------------------------------------------------- */
/* klu_free_symbolic: destroys the Symbolic object */
/* -------------------------------------------------------------------------- */

int klu_free_symbolic
(
    klu_symbolic **Symbolic,
    klu_common *Common
) ;

SuiteSparse_long klu_l_free_symbolic (klu_l_symbolic **, klu_l_common *) ;


/* -------------------------------------------------------------------------- */
/* klu_free_numeric: destroys the Numeric object */
/* -------------------------------------------------------------------------- */

/* Note that klu_free_numeric and klu_z_free_numeric are identical; each can
 * free both kinds of Numeric objects (real and complex) */

int klu_free_numeric
(
    klu_numeric **Numeric,
    klu_common *Common
) ;

int klu_z_free_numeric
(
     klu_numeric **Numeric,
     klu_common *Common
) ;

SuiteSparse_long klu_l_free_numeric (klu_l_numeric **, klu_l_common *) ;
SuiteSparse_long klu_zl_free_numeric (klu_l_numeric **, klu_l_common *) ;


/* -------------------------------------------------------------------------- */
/* klu_sort: sorts the columns of the LU factorization */
/* -------------------------------------------------------------------------- */

/* this is not needed except for the MATLAB interface */
```

```
int klu_sort
(
    /* inputs, not modified */
    klu_symbolic *Symbolic,
    /* input/output */
    klu_numeric *Numeric,
    klu_common *Common
) ;

int klu_z_sort
(
    /* inputs, not modified */
    klu_symbolic *Symbolic,
    /* input/output */
    klu_numeric *Numeric,
    klu_common *Common
) ;

SuiteSparse_long klu_l_sort (klu_l_symbolic *, klu_l_numeric *,
    klu_l_common *) ;
SuiteSparse_long klu_zl_sort (klu_l_symbolic *, klu_l_numeric *,
    klu_l_common *) ;


/* -------------------------------------------------------------------------- */
/* klu_flops: determines # of flops performed in numeric factorzation */
/* -------------------------------------------------------------------------- */

int klu_flops
(
    /* inputs, not modified */
    klu_symbolic *Symbolic,
    klu_numeric *Numeric,
    /* input/output */
    klu_common *Common
) ;

int klu_z_flops
(
    /* inputs, not modified */
    klu_symbolic *Symbolic,
    klu_numeric *Numeric,
    /* input/output */
    klu_common *Common
) ;

SuiteSparse_long klu_l_flops (klu_l_symbolic *, klu_l_numeric *,
    klu_l_common *) ;
SuiteSparse_long klu_zl_flops (klu_l_symbolic *, klu_l_numeric *,
    klu_l_common *) ;


/* -------------------------------------------------------------------------- */
/* klu_rgrowth : compute the reciprocal pivot growth */
/* -------------------------------------------------------------------------- */

/* Pivot growth is computed after the input matrix is permuted, scaled, and
 * off-diagonal entries pruned.  This is because the LU factorization of each
 * block takes as input the scaled diagonal blocks of the BTF form.  The
```

```
 * reciprocal pivot growth in column j of an LU factorization of a matrix C
 * is the largest entry in C divided by the largest entry in U; then the overall
 * reciprocal pivot growth is the smallest such value for all columns j.  Note
 * that the off-diagonal entries are not scaled, since they do not take part in
 * the LU factorization of the diagonal blocks.
 *
 * In MATLAB notation:
 *
 * rgrowth = min (max (abs ((R \ A(p,q)) - F)) ./ max (abs (U))) */

int klu_rgrowth
(
    int Ap [ ],
    int Ai [ ],
    double Ax [ ],
    klu_symbolic *Symbolic,
    klu_numeric *Numeric,
    klu_common *Common          /* Common->rgrowth = reciprocal pivot growth */
) ;

int klu_z_rgrowth
(
    int Ap [ ],
    int Ai [ ],
    double Ax [ ],
    klu_symbolic *Symbolic,
    klu_numeric *Numeric,
    klu_common *Common          /* Common->rgrowth = reciprocal pivot growth */
) ;

SuiteSparse_long klu_l_rgrowth (SuiteSparse_long *, SuiteSparse_long *,
    double *, klu_l_symbolic *, klu_l_numeric *, klu_l_common *) ;

SuiteSparse_long klu_zl_rgrowth (SuiteSparse_long *, SuiteSparse_long *,
    double *, klu_l_symbolic *, klu_l_numeric *, klu_l_common *) ;


/* -------------------------------------------------------------------------- */
/* klu_condest */
/* -------------------------------------------------------------------------- */

/* Computes a reasonably accurate estimate of the 1-norm condition number, using
 * Hager's method, as modified by Higham and Tisseur (same method as used in
 * MATLAB's condest */

int klu_condest
(
    int Ap [ ],             /* size n+1, column pointers, not modified */
    double Ax [ ],          /* size nz = Ap[n], numerical values, not modified*/
    klu_symbolic *Symbolic, /* symbolic analysis, not modified */
    klu_numeric *Numeric,   /* numeric factorization, not modified */
    klu_common *Common      /* result returned in Common->condest */
) ;

int klu_z_condest
(
    int Ap [ ],
    double Ax [ ],          /* size 2*nz */
    klu_symbolic *Symbolic,
```

```
    klu_numeric *Numeric,
    klu_common *Common      /* result returned in Common->condest */
) ;

SuiteSparse_long klu_l_condest (SuiteSparse_long *, double *, klu_l_symbolic *,
    klu_l_numeric *, klu_l_common *) ;

SuiteSparse_long klu_zl_condest (SuiteSparse_long *, double *, klu_l_symbolic *,
    klu_l_numeric *, klu_l_common *) ;


/* -------------------------------------------------------------------------- */
/* klu_rcond: compute min(abs(diag(U))) / max(abs(diag(U))) */
/* -------------------------------------------------------------------------- */

int klu_rcond
(
    klu_symbolic *Symbolic,         /* input, not modified */
    klu_numeric *Numeric,           /* input, not modified */
    klu_common *Common              /* result in Common->rcond */
) ;

int klu_z_rcond
(
    klu_symbolic *Symbolic,         /* input, not modified */
    klu_numeric *Numeric,           /* input, not modified */
    klu_common *Common              /* result in Common->rcond */
) ;

SuiteSparse_long klu_l_rcond (klu_l_symbolic *, klu_l_numeric *,
    klu_l_common *) ;

SuiteSparse_long klu_zl_rcond (klu_l_symbolic *, klu_l_numeric *,
    klu_l_common *) ;


/* -------------------------------------------------------------------------- */
/* klu_scale */
/* -------------------------------------------------------------------------- */

int klu_scale           /* return TRUE if successful, FALSE otherwise */
(
    /* inputs, not modified */
    int scale,          /* <0: none, no error check; 0: none, 1: sum, 2: max */
    int n,
    int Ap [ ],         /* size n+1, column pointers */
    int Ai [ ],         /* size nz, row indices */
    double Ax [ ],
    /* outputs, not defined on input */
    double Rs [ ],
    /* workspace, not defined on input or output */
    int W [ ],          /* size n, can be NULL */
    klu_common *Common
) ;

int klu_z_scale         /* return TRUE if successful, FALSE otherwise */
(
    /* inputs, not modified */
    int scale,          /* <0: none, no error check; 0: none, 1: sum, 2: max */
```

```
    int n,
    int Ap [ ],         /* size n+1, column pointers */
    int Ai [ ],         /* size nz, row indices */
    double Ax [ ],
    /* outputs, not defined on input */
    double Rs [ ],
    /* workspace, not defined on input or output */
    int W [ ],          /* size n, can be NULL */
    klu_common *Common
) ;

SuiteSparse_long klu_l_scale (SuiteSparse_long, SuiteSparse_long,
    SuiteSparse_long *, SuiteSparse_long *, double *,
    double *, SuiteSparse_long *, klu_l_common *) ;

SuiteSparse_long klu_zl_scale (SuiteSparse_long, SuiteSparse_long,
    SuiteSparse_long *, SuiteSparse_long *, double *,
    double *, SuiteSparse_long *, klu_l_common *) ;


/* -------------------------------------------------------------------------- */
/* klu_extract  */
/* -------------------------------------------------------------------------- */

int klu_extract     /* returns TRUE if successful, FALSE otherwise */
(
    /* inputs: */
    klu_numeric *Numeric,
    klu_symbolic *Symbolic,

    /* outputs, either allocated on input, or ignored otherwise */

    /* L */
    int *Lp,        /* size n+1 */
    int *Li,        /* size Numeric->lnz */
    double *Lx,     /* size Numeric->lnz */

    /* U */
    int *Up,        /* size n+1 */
    int *Ui,        /* size Numeric->unz */
    double *Ux,     /* size Numeric->unz */

    /* F */
    int *Fp,        /* size n+1 */
    int *Fi,        /* size Numeric->nzoff */
    double *Fx,     /* size Numeric->nzoff */

    /* P, row permutation */
    int *P,         /* size n */

    /* Q, column permutation */
    int *Q,         /* size n */

    /* Rs, scale factors */
    double *Rs,     /* size n */

    /* R, block boundaries */
    int *R,         /* size Symbolic->nblocks+1 (nblocks is at most n) */
```

```
    klu_common *Common
) ;


int klu_z_extract           /* returns TRUE if successful, FALSE otherwise */
(
    /* inputs: */
    klu_numeric *Numeric,
    klu_symbolic *Symbolic,

    /* outputs, all of which must be allocated on input */

    /* L */
    int *Lp,        /* size n+1 */
    int *Li,        /* size nnz(L) */
    double *Lx,     /* size nnz(L) */
    double *Lz,     /* size nnz(L) for the complex case, ignored if real */

    /* U */
    int *Up,        /* size n+1 */
    int *Ui,        /* size nnz(U) */
    double *Ux,     /* size nnz(U) */
    double *Uz,     /* size nnz(U) for the complex case, ignored if real */

    /* F */
    int *Fp,        /* size n+1 */
    int *Fi,        /* size nnz(F) */
    double *Fx,     /* size nnz(F) */
    double *Fz,     /* size nnz(F) for the complex case, ignored if real */

    /* P, row permutation */
    int *P,         /* size n */

    /* Q, column permutation */
    int *Q,         /* size n */

    /* Rs, scale factors */
    double *Rs,     /* size n */

    /* R, block boundaries */
    int *R,         /* size Symbolic->nblocks+1 (nblocks is at most n) */

    klu_common *Common
) ;

SuiteSparse_long klu_l_extract (klu_l_numeric *, klu_l_symbolic *,
    SuiteSparse_long *, SuiteSparse_long *, double *,
    SuiteSparse_long *, SuiteSparse_long *, double *,
    SuiteSparse_long *, SuiteSparse_long *, double *,
    SuiteSparse_long *, SuiteSparse_long *, double *,
    SuiteSparse_long *, klu_l_common *) ;

SuiteSparse_long klu_zl_extract (klu_l_numeric *, klu_l_symbolic *,
    SuiteSparse_long *, SuiteSparse_long *, double *, double *,
    SuiteSparse_long *, SuiteSparse_long *, double *, double *,
    SuiteSparse_long *, SuiteSparse_long *, double *, double *,
    SuiteSparse_long *, SuiteSparse_long *, double *,
    SuiteSparse_long *, klu_l_common *) ;
```

```
/* -------------------------------------------------------------------------- */
/* KLU memory management routines */
/* -------------------------------------------------------------------------- */

void *klu_malloc        /* returns pointer to the newly malloc'd block */
(
    /* ---- input ---- */
    size_t n,           /* number of items */
    size_t size,        /* size of each item */
    /* --------------- */
    klu_common *Common
) ;

void *klu_free          /* always returns NULL */
(
    /* ---- in/out --- */
    void *p,            /* block of memory to free */
    size_t n,           /* number of items */
    size_t size,        /* size of each item */
    /* --------------- */
    klu_common *Common
) ;

void *klu_realloc       /* returns pointer to reallocated block */
(
    /* ---- input ---- */
    size_t nnew,        /* requested # of items in reallocated block */
    size_t nold,        /* current size of block, in # of items */
    size_t size,        /* size of each item */
    /* ---- in/out --- */
    void *p,            /* block of memory to realloc */
    /* --------------- */
    klu_common *Common
) ;

void *klu_l_malloc (size_t, size_t, klu_l_common *) ;
void *klu_l_free (void *, size_t, size_t, klu_l_common *) ;
void *klu_l_realloc (size_t, size_t, size_t, void *, klu_l_common *) ;


/* ========================================================================== */
/* === KLU version ========================================================== */
/* ========================================================================== */

/* All versions of KLU include these definitions.
 * As an example, to test if the version you are using is 1.2 or later:
 *
 *      if (KLU_VERSION >= KLU_VERSION_CODE (1,2)) ...
 *
 * This also works during compile-time:
 *
 *      #if (KLU >= KLU_VERSION_CODE (1,2))
 *          printf ("This is version 1.2 or later\n") ;
 *      #else
 *          printf ("This is an early version\n") ;
 *      #endif
 */
```

```
#define KLU_DATE "Oct 23, 2014"
#define KLU_VERSION_CODE(main,sub) ((main) * 1000 + (sub))
#define KLU_MAIN_VERSION 1
#define KLU_SUB_VERSION 3
#define KLU_SUBSUB_VERSION 2
#define KLU_VERSION KLU_VERSION_CODE(KLU_MAIN_VERSION,KLU_SUB_VERSION)

#ifdef __cplusplus
}
#endif
#endif
```

8 The BTF routines

The file `BTF/Include/btf.h` listed below describes each user-callable routine in the C version of BTF, and gives details on their use.

```
/* ========================================================================== */
/* === BTF package ========================================================== */
/* ========================================================================== */

/* BTF_MAXTRANS:  find a column permutation Q to give A*Q a zero-free diagonal
 * BTF_STRONGCOMP:  find a symmetric permutation P to put P*A*P' into block
 *      upper triangular form.
 * BTF_ORDER: do both of the above (btf_maxtrans then btf_strongcomp).
 *
 * By Tim Davis.  Copyright (c) 2004-2007, University of Florida.
 * with support from Sandia National Laboratories.  All Rights Reserved.
 */


/* ========================================================================== */
/* === BTF_MAXTRANS ========================================================= */
/* ========================================================================== */

/* BTF_MAXTRANS: finds a permutation of the columns of a matrix so that it has a
 * zero-free diagonal.  The input is an m-by-n sparse matrix in compressed
 * column form.  The array Ap of size n+1 gives the starting and ending
 * positions of the columns in the array Ai.  Ap[0] must be zero. The array Ai
 * contains the row indices of the nonzeros of the matrix A, and is of size
 * Ap[n].  The row indices of column j are located in Ai[Ap[j] ... Ap[j+1]-1].
 * Row indices must be in the range 0 to m-1.  Duplicate entries may be present
 * in any given column.  The input matrix  is not checked for validity (row
 * indices out of the range 0 to m-1 will lead to an undeterminate result -
 * possibly a core dump, for example).  Row indices in any given column need
 * not be in sorted order.  However, if they are sorted and the matrix already
 * has a zero-free diagonal, then the identity permutation is returned.
 *
 * The output of btf_maxtrans is an array Match of size n.  If row i is matched
 * with column j, then A(i,j) is nonzero, and then Match[i] = j.  If the matrix
 * is structurally nonsingular, all entries in the Match array are unique, and
 * Match can be viewed as a column permutation if A is square.  That is, column
 * k of the original matrix becomes column Match[k] of the permuted matrix.  In
 * MATLAB, this can be expressed as (for non-structurally singular matrices):
 *
 *      Match = maxtrans (A) ;
 *      B = A (:, Match) ;
 *
 * except of course here the A matrix and Match vector are all 0-based (rows
 * and columns in the range 0 to n-1), not 1-based (rows/cols in range 1 to n).
 * The MATLAB dmperm routine returns a row permutation.  See the maxtrans
 * mexFunction for more details.
 *
 * If row i is not matched to any column, then Match[i] is == -1.  The
 * btf_maxtrans routine returns the number of nonzeros on diagonal of the
 * permuted matrix.
 *
 * In the MATLAB mexFunction interface to btf_maxtrans, 1 is added to the Match
 * array to obtain a 1-based permutation.  Thus, in MATLAB where A is m-by-n:
 *
 *      q = maxtrans (A) ;      % has entries in the range 0:n
```

```
 *      q                       % a column permutation (only if sprank(A)==n)
 *      B = A (:, q) ;          % permuted matrix (only if sprank(A)==n)
 *      sum (q > 0) ;           % same as "sprank (A)"
 *
 * This behaviour differs from p = dmperm (A) in MATLAB, which returns the
 * matching as p(j)=i if row i and column j are matched, and p(j)=0 if column j
 * is unmatched.
 *
 *      p = dmperm (A) ;        % has entries in the range 0:m
 *      p                       % a row permutation (only if sprank(A)==m)
 *      B = A (p, :) ;          % permuted matrix (only if sprank(A)==m)
 *      sum (p > 0) ;           % definition of sprank (A)
 *
 * This algorithm is based on the paper "On Algorithms for obtaining a maximum
 * transversal" by Iain Duff, ACM Trans. Mathematical Software, vol 7, no. 1,
 * pp. 315-330, and "Algorithm 575: Permutations for a zero-free diagonal",
 * same issue, pp. 387-390.  Algorithm 575 is MC21A in the Harwell Subroutine
 * Library.  This code is not merely a translation of the Fortran code into C.
 * It is a completely new implementation of the basic underlying method (depth
 * first search over a subgraph with nodes corresponding to columns matched so
 * far, and cheap matching).  This code was written with minimal observation of
 * the MC21A/B code itself.  See comments below for a comparison between the
 * maxtrans and MC21A/B codes.
 *
 * This routine operates on a column-form matrix and produces a column
 * permutation.  MC21A uses a row-form matrix and produces a row permutation.
 * The difference is merely one of convention in the comments and interpretation
 * of the inputs and outputs.  If you want a row permutation, simply pass a
 * compressed-row sparse matrix to this routine and you will get a row
 * permutation (just like MC21A).  Similarly, you can pass a column-oriented
 * matrix to MC21A and it will happily return a column permutation.
 */

#ifndef _BTF_H
#define _BTF_H

/* make it easy for C++ programs to include BTF */
#ifdef __cplusplus
extern "C" {
#endif

#include "SuiteSparse_config.h"

int btf_maxtrans    /* returns # of columns matched */
(
    /* --- input, not modified: --- */
    int nrow,       /* A is nrow-by-ncol in compressed column form */
    int ncol,
    int Ap [ ],     /* size ncol+1 */
    int Ai [ ],     /* size nz = Ap [ncol] */
    double maxwork, /* maximum amount of work to do is maxwork*nnz(A); no limit
                     * if <= 0 */

    /* --- output, not defined on input --- */
    double *work,   /* work = -1 if maxwork > 0 and the total work performed
                     * reached the maximum of maxwork*nnz(A).
                     * Otherwise, work = the total work performed. */

    int Match [ ],  /* size nrow.  Match [i] = j if column j matched to row i
```

```
                     * (see above for the singular-matrix case) */

    /* --- workspace, not defined on input or output --- */
    int Work [ ]    /* size 5*ncol */
) ;

/* long integer version (all "int" parameters become "SuiteSparse_long") */
SuiteSparse_long btf_l_maxtrans (SuiteSparse_long, SuiteSparse_long,
    SuiteSparse_long *, SuiteSparse_long *, double, double *,
    SuiteSparse_long *, SuiteSparse_long *) ;


/* ========================================================================== */
/* === BTF_STRONGCOMP ======================================================= */
/* ========================================================================== */

/* BTF_STRONGCOMP finds the strongly connected components of a graph, returning
 * a symmetric permutation.  The matrix A must be square, and is provided on
 * input in compressed-column form (see BTF_MAXTRANS, above).  The diagonal of
 * the input matrix A (or A*Q if Q is provided on input) is ignored.
 *
 * If Q is not NULL on input, then the strongly connected components of A*Q are
 * found.  Q may be flagged on input, where Q[k] < 0 denotes a flagged column k.
 * The permutation is j = BTF_UNFLIP (Q [k]).  On output, Q is modified (the
 * flags are preserved) so that P*A*Q is in block upper triangular form.
 *
 * If Q is NULL, then the permutation P is returned so that P*A*P' is in upper
 * block triangular form.
 *
 * The vector R gives the block boundaries, where block b is in rows/columns
 * R[b] to R[b+1]-1 of the permuted matrix, and where b ranges from 1 to the
 * number of strongly connected components found.
 */

int btf_strongcomp  /* return # of strongly connected components */
(
    /* input, not modified: */
    int n,          /* A is n-by-n in compressed column form */
    int Ap [ ],     /* size n+1 */
    int Ai [ ],     /* size nz = Ap [n] */

    /* optional input, modified (if present) on output: */
    int Q [ ],      /* size n, input column permutation */

    /* output, not defined on input */
    int P [ ],      /* size n.  P [k] = j if row and column j are kth row/col
                     * in permuted matrix. */

    int R [ ],      /* size n+1.  block b is in rows/cols R[b] ... R[b+1]-1 */

    /* workspace, not defined on input or output */
    int Work [ ]    /* size 4n */
) ;

SuiteSparse_long btf_l_strongcomp (SuiteSparse_long, SuiteSparse_long *,
    SuiteSparse_long *, SuiteSparse_long *, SuiteSparse_long *,
    SuiteSparse_long *, SuiteSparse_long *) ;
```

```
/* ========================================================================== */
/* === BTF_ORDER ============================================================ */
/* ========================================================================== */

/* BTF_ORDER permutes a square matrix into upper block triangular form.  It
 * does this by first finding a maximum matching (or perhaps a limited matching
 * if the work is limited), via the btf_maxtrans function.  If a complete
 * matching is not found, BTF_ORDER completes the permutation, but flags the
 * columns of P*A*Q to denote which columns are not matched.  If the matrix is
 * structurally rank deficient, some of the entries on the diagonal of the
 * permuted matrix will be zero.  BTF_ORDER then calls btf_strongcomp to find
 * the strongly-connected components.
 *
 * On output, P and Q are the row and column permutations, where i = P[k] if
 * row i of A is the kth row of P*A*Q, and j = BTF_UNFLIP(Q[k]) if column j of
 * A is the kth column of P*A*Q.  If Q[k] < 0, then the (k,k)th entry in P*A*Q
 * is structurally zero.
 *
 * The vector R gives the block boundaries, where block b is in rows/columns
 * R[b] to R[b+1]-1 of the permuted matrix, and where b ranges from 1 to the
 * number of strongly connected components found.
 */

int btf_order       /* returns number of blocks found */
(
    /* --- input, not modified: --- */
    int n,          /* A is n-by-n in compressed column form */
    int Ap [ ],     /* size n+1 */
    int Ai [ ],     /* size nz = Ap [n] */
    double maxwork, /* do at most maxwork*nnz(A) work in the maximum
                     * transversal; no limit if <= 0 */

    /* --- output, not defined on input --- */
    double *work,   /* return value from btf_maxtrans */
    int P [ ],      /* size n, row permutation */
    int Q [ ],      /* size n, column permutation */
    int R [ ],      /* size n+1.  block b is in rows/cols R[b] ... R[b+1]-1 */
    int *nmatch,    /* # nonzeros on diagonal of P*A*Q */

    /* --- workspace, not defined on input or output --- */
    int Work [ ]    /* size 5n */
) ;

SuiteSparse_long btf_l_order (SuiteSparse_long, SuiteSparse_long *,
    SuiteSparse_long *, double , double *, SuiteSparse_long *,
    SuiteSparse_long *, SuiteSparse_long *, SuiteSparse_long *,
    SuiteSparse_long *) ;


/* ========================================================================== */
/* === BTF marking of singular columns ====================================== */
/* ========================================================================== */

/* BTF_FLIP is a "negation about -1", and is used to mark an integer j
 * that is normally non-negative.  BTF_FLIP (-1) is -1.  BTF_FLIP of
 * a number > -1 is negative, and BTF_FLIP of a number < -1 is positive.
 * BTF_FLIP (BTF_FLIP (j)) = j for all integers j.  UNFLIP (j) acts
 * like an "absolute value" operation, and is always >= -1.  You can test
 * whether or not an integer j is "flipped" with the BTF_ISFLIPPED (j)
```

```
 * macro.
 */

#define BTF_FLIP(j) (-(j)-2)
#define BTF_ISFLIPPED(j) ((j) < -1)
#define BTF_UNFLIP(j) ((BTF_ISFLIPPED (j)) ? BTF_FLIP (j) : (j))

/* ========================================================================== */
/* === BTF version ========================================================== */
/* ========================================================================== */

/* All versions of BTF include these definitions.
 * As an example, to test if the version you are using is 1.2 or later:
 *
 *      if (BTF_VERSION >= BTF_VERSION_CODE (1,2)) ...
 *
 * This also works during compile-time:
 *
 *      #if (BTF >= BTF_VERSION_CODE (1,2))
 *          printf ("This is version 1.2 or later\n") ;
 *      #else
 *          printf ("This is an early version\n") ;
 *      #endif
 */

#define BTF_DATE "Oct 10, 2014"
#define BTF_VERSION_CODE(main,sub) ((main) * 1000 + (sub))
#define BTF_MAIN_VERSION 1
#define BTF_SUB_VERSION 2
#define BTF_SUBSUB_VERSION 1
#define BTF_VERSION BTF_VERSION_CODE(BTF_MAIN_VERSION,BTF_SUB_VERSION)

#ifdef __cplusplus
}
#endif
#endif
```

References

[1] P. R. Amestoy, T. A. Davis, and I. S. Duff. An approximate minimum degree ordering algorithm. *SIAM J. Matrix Anal. Appl.*, 17:886–905, 1996.

[2] P. R. Amestoy, T. A. Davis, and I. S. Duff. Algorithm 837: AMD, an approximate minimum degree ordering algorithm. *ACM Trans. Math. Software*, 30:381–388, 2004.

[3] Y. Chen, T. A. Davis, W. W. Hager, and S. Rajamanickam. Algorithm 887: CHOLMOD, supernodal sparse Cholesky factorization and update/downdate. *ACM Trans. Math. Software*, 35(3), 2009.

[4] T. A. Davis. Algorithm 832: UMFPACK V4.3, an unsymmetric-pattern multifrontal method. *ACM Trans. Math. Software*, 30:196–199, 2002.

[5] T. A. Davis. A column pre-ordering strategy for the unsymmetric-pattern multifrontal method. *ACM Trans. Math. Software*, 30:165–195, 2004.

[6] T. A. Davis. *Direct Methods for Sparse Linear Systems.* SIAM, Philadelphia, PA, 2006.

[7] T. A. Davis, J. R. Gilbert, S. I. Larimore, and E. G. Ng. Algorithm 836: COLAMD, a column approximate minimum degree ordering algorithm. *ACM Trans. Math. Software*, 30:377–380, 2004.

[8] T. A. Davis, J. R. Gilbert, S. I. Larimore, and E. G. Ng. A column approximate minimum degree ordering algorithm. *ACM Trans. Math. Software*, 30:353–376, 2004.

[9] Timothy A. Davis and Ekanathan Palamadai Natarajan. Algorithm 907: KLU, a direct sparse solver for circuit simulation problems. *ACM Trans. Math. Softw.*, 37:36:1–36:17, September 2010.

[10] J. W. Demmel, S. C. Eisenstat, J. R. Gilbert, X. S. Li, and J. W. H. Liu. A supernodal approach to sparse partial pivoting. *SIAM J. Matrix Anal. Appl.*, 20:720–755, 1999.

[11] J. J. Dongarra, J. Du Croz, I. S. Duff, and S. Hammarling. A set of level-3 basic linear algebra subprograms. *ACM Trans. Math. Software*, 16:1–17, 1990.

[12] I. S. Duff. On algorithms for obtaining a maximum transversal. *ACM Trans. Math. Software*, 7:315–330, 1981.

[13] I. S. Duff and J. K. Reid. An implementation of Tarjan's algorithm for the block triangularization of a matrix. *ACM Trans. Math. Software*, 4:137–147, 1978.

[14] J. R. Gilbert and T. Peierls. Sparse partial pivoting in time proportional to arithmetic operations. *SIAM J. Sci. Statist. Comput.*, 9:862–874, 1988.

[15] W. W. Hager. Condition estimates. *SIAM J. Sci. Statist. Comput.*, 5:311–316, 1984.

[16] N. J. Higham and F. Tisseur. A block algorithm for matrix 1-norm estimation with an application to 1-norm pseudospectra. *SIAM J. Matrix Anal. Appl.*, 21:1185–1201, 2000.

[17] G. Karypis and V. Kumar. A fast and high quality multilevel scheme for partitioning irregular graphs. *SIAM J. Sci. Comput.*, 20, 1998.

[18] K. S. Kundert. Sparse matrix techniques and their applications to circuit simulation. In A. E. Ruehli, editor, *Circuit Analysis, Simulation and Design*. New York: North-Holland, 1986.

[19] K. S. Kundert and A. Sangiovanni-Vincentelli. User's guide: Sparse1.2. Technical report, Dept. of EE and CS, UC Berkeley, Oct. 1985.

[20] L. W Nagel and D. O. Pederson. SPICE (simulation program with integrated circuit emphasis). Technical Report Memorandum No. ERL-M382, University of California, Berkeley, 1973.

[21] E. Palamadai. KLU - a high performance sparse linear system solver for circuit simulation problems. Technical report, CISE Department, Univ. of Florida. M.S. Thesis.

[22] Thomas L. Quarles. *Analysis of Performance and Convergence Issues for Circuit Simulation.* PhD thesis, EECS Department, University of California, Berkeley, 1989.

[23] R. E. Tarjan. Depth first search and linear graph algorithms. *SIAM J. Comput.*, 1:146–160, 1972.
