# Supplementary material for: Inference for Stochastic Chemical Kinetics Using Moment Equations and System Size Expansion
Source: PLoS Comput Biol. 2016 Jul 22;12(7):e1005030. doi: 10.1371/journal.pcbi.1005030 (PMC4957800; doi:10.1371/journal.pcbi.1005030)
Supplement: S1 Code — This zip-file contains the MATLAB code for the simulation and application example presented in the paper. We provide implementations of all models, parameter estimation and uncertainty analysis to allow everybody to reproduce the results. (ZIP) [file pcbi.1005030.s002.zip › S1code/cvodewrap/SuiteSparse/KLU/Doc/palamadai_e.pdf]

KLU–A HIGH PERFORMANCE SPARSE LINEAR SOLVER FOR CIRCUIT SIMULATION PROBLEMS

By

EKANATHAN PALAMADAI NATARAJAN

A THESIS PRESENTED TO THE GRADUATE SCHOOL
OF THE UNIVERSITY OF FLORIDA IN PARTIAL FULFILLMENT
OF THE REQUIREMENTS FOR THE DEGREE OF
MASTER OF SCIENCE

UNIVERSITY OF FLORIDA

2005

Copyright 2005

by

Ekanathan Palamadai Natarajan

I dedicate this work to my mother Savitri who has been a source of inspiration and support to me.

ACKNOWLEDGMENTS

I would like to thank Dr. Timothy Davis, my advisor for introducing me to the area of sparse matrix algorithms and linear solvers. I started only with my background in numerical analysis and algorithms, a year and half back. The insights and knowledge I have gained since then in the area and in implementing a sparse linear solver like KLU would not have been possible but for Dr. Davis' guidance and help. I thank him for giving me an opportunity to work on KLU. I would like to thank Dr. Jose Fortes and Dr. Arunava Banerjee for their support and help and for serving on my committee.

I would like to thank CISE administrative staff for helping me at different times during my master's research work.

TABLE OF CONTENTS

page

ACKNOWLEDGMENTS . . . iv

LIST OF TABLES . . . vii

LIST OF FIGURES . . . viii

ABSTRACT . . . ix

CHAPTER

1 INTRODUCTION . . . 1

2 THEORY: SPARSE LU . . . 5

2.1 Dense LU . . . 5
2.2 Sparse LU . . . 7
2.3 Left Looking Gaussian Elimination . . . 8
2.4 Gilbert-Peierls' Algorithm . . . 10
2.4.1 Symbolic Analysis . . . 11
2.4.2 Numerical Factorization . . . 13
2.5 Maximum Transversal . . . 14
2.6 Block Triangular Form . . . 16
2.7 Symmetric Pruning . . . 18
2.8 Ordering . . . 19
2.9 Pivoting . . . 22
2.10 Scaling . . . 23
2.11 Growth Factor . . . 25
2.12 Condition Number . . . 27
2.13 Depth First Search . . . 30
2.14 Memory Fragmentation . . . 31
2.15 Complex Number Support . . . 33
2.16 Parallelism in KLU . . . 33

3 CIRCUIT SIMULATION: APPLICATION OF KLU . . . 35

3.1 Characteristics of Circuit Matrices . . . 37
3.2 Linear Systems in Circuit Simulation . . . 38
3.3 Performance Benchmarks . . . 39
3.4 Analyses and Findings . . . 41
3.5 Alternate Ordering Experiments . . . 42

3.6 Experiments with UF Sparse Matrix Collection 44
3.6.1 Different Ordering Schemes in KLU 44
3.6.2 Timing Different Phases in KLU 45
3.6.3 Ordering Quality among KLU, UMFPACK and Gilbert-Peierls 45
3.6.4 Performance Comparison between KLU and UMFPACK . . 48

4 USER GUIDE FOR KLU 51

4.1 The Primary KLU Structures 51
4.1.1 klu_common 51
4.1.2 klu_symbolic 53
4.1.3 klu_numeric 55
4.2 KLU Routines 58
4.2.1 klu_analyze 58
4.2.2 klu_analyze_given 59
4.2.3 klu_*factor 59
4.2.4 klu_*solve 60
4.2.5 klu_*tsolve 61
4.2.6 klu_*refactor 62
4.2.7 klu_defaults 63
4.2.8 klu_*rec_pivot_growth 63
4.2.9 klu_*estimate_cond_number 64
4.2.10 klu_free_symbolic 65
4.2.11 klu_free_numeric 66

REFERENCES 67

BIOGRAPHICAL SKETCH 69

LIST OF TABLES

Table page

3–1 Comparison between KLU and SuperLU on overall time and fill-in . . 39

3–2 Comparison between KLU and SuperLU on factor time and solve time 40

3–3 Ordering results using BTF+AMD in KLU on circuit matrices 41

3–4 Comparison of ordering results produced by BTF+AMD, AMD, MMD 43

3–5 Fill-in with four different schemes in KLU 46

3–6 Time in seconds, spent in different phases in KLU 47

3–7 Fill-in among KLU, UMFPACK and Gilbert-Peierls 49

3–8 Performance comparison between KLU and UMFPACK 50

LIST OF FIGURES

Figure page

2–1 Nonzero pattern of x when solving Lx = b 13

2–2 A matrix permuted to BTF form 16

2–3 A symmetric pruning scenario 18

2–4 A symmetric matrix and its graph representation 21

2–5 The matrix and its graph representation after one step of Gaussian elimination 21

2–6 A doubly bordered block diagonal matrix and its corresponding vertex separator tree 34

Abstract of Thesis Presented to the Graduate School
of the University of Florida in Partial Fulfillment of the
Requirements for the Degree of Master of Science

KLU–A HIGH PERFORMANCE SPARSE LINEAR SOLVER
FOR CIRCUIT SIMULATION PROBLEMS

By

Ekanathan Palamadai Natarajan

August 2005

Chair: Dr. Timothy A. Davis
Major Department: Computer and Information Science and Engineering

The thesis work focuses on KLU, a sparse high performance linear solver for circuit simulation matrices. During the circuit simulation process, one of the key steps is to solve sparse systems of linear equations of very high order. KLU targets solving these systems with efficient ordering mechanisms and high performance factorization and solve algorithms. KLU uses a hybrid ordering strategy that comprises an unsymmetric permutation to ensure zero free diagonal, a symmetric permutation to block upper triangular form and a fill reducing ordering such as approximate minimum degree.

The factorization is based on Gilbert-Peierls' left-looking algorithm with partial pivoting. KLU also includes features like symmetric pruning to cut down symbolic analysis costs. It offers to solve up to four right hand sides in a single solve step. In addition, it offers transpose and conjugate transpose solve capabilities and important diagnostic features to estimate the reciprocal pivot growth of the factorization and condition number of the input matrix.

The algorithm is implemented in the C language with MATLAB interfaces as well. The MATLAB interfaces enable a user to invoke KLU routines from within

the MATLAB environment. The implementation was tested on circuit matrices and the results determined. KLU achieves superior fill-in quality with its hybrid ordering strategy and achieves a good performance speed-up when compared with existing sparse linear solvers for circuit problems. The thesis highlights the work being done on exploiting parallelism in KLU as well.

CHAPTER 1
INTRODUCTION

Sparse is beautiful. Solving systems of linear equations of the form $Ax = b$ is a fundamental and important area of high performance computing. The matrix A is called the coefficient matrix and b the right hand side vector. The vector x is the solution to the equation. There are a number of methods available for solving such systems. Some of the popular ones are Gaussian elimination, QR factorization using Householder transformations or Givens rotations and Cholesky factorization. Gaussian elimination with partial pivoting is the most widely used algorithm for solving linear systems because of its stability and better time complexity. Cholesky can be used only when A is symmetric positive definite.

Some systems that are solved comprise a dense coefficient matrix A. By dense, we mean most of the elements in A are nonzero. There are high performance subroutines such as the BLAS [1, 2, 3, 4, 5] that can maximize flop count for such dense matrices. The interesting systems are those where the coefficient matrix A happens to be sparse. By sparse, we mean the matrix has few nonzero entries(hereafter referred to simply as 'nonzeros'). The adjective 'few' is not well-defined as we will see in chapter two. When matrices tend to be sparse, we need to find out effective ways to store the matrix in memory since we want to avoid storing zeros of the matrix. When we store only the nonzeros in the matrix, it has consequences in the factorization algorithm as well. One typical example would be we do not know before hand how nonzeros would appear in the L and U factors when we factorize the matrix. While we avoid storing the zeros, we also want to achieve good time complexity when solving sparse systems. If the time spent to

solve sparse systems remains same as for dense systems, we have not done any better.

KLU stands for Clark Kent LU, since it is based on Gilbert-Peierls' algorithm, a non-supernodal algorithm, which is the predecessor to SuperLU, a supernodal algorithm. KLU is a sparse high performance linear solver that employs hybrid ordering mechanisms and elegant factorization and solve algorithms. It achieves high quality fill-in rate and beats many existing solvers in run time, when used for matrices arising in circuit simulation.

There are several flavours of Gaussian elimination. A left-looking Gaussian elimination algorithm factorizes the matrix left to right computing columns of L and U. A right-looking version factorizes the matrix from top-left to bottom-right computing column of L and row of U. Both have their advantages and disadvantages. KLU uses a left looking algorithm called Gilbert-Peierls' algorithm. Gilbert-Peierls' comprises a graph theoretical symbolic analysis phase that identifies the nonzero pattern of each column of L and U factors and a left-looking numerical factorization phase with partial pivoting that calculates the numerical values of the factors. KLU uses Symmetric Pruning to cut down symbolic analysis cost. We shall look in detail on these features in chapter two.

A critical issue in linear solvers is Ordering. Ordering means permuting the rows and columns of a matrix, so that the fill-in in the L and U factors is reduced to a minimum. A fill-in is defined as a nonzero appearing in either of the matrices L or U, while the element in the corresponding position in A is a zero. L_{ij} or U_{ij} is a fill-in if A_{ij} is a zero. Fill-in has obvious consequences in memory in that the factorization algorithm could create dense L and U factors that can exhaust available memory. A good ordering algorithm yields a low fill-in in the factors. Finding the ordering that gives minimal fill-in is an NP complete problem. So

ordering algorithms use heuristics. KLU accomodates multiple ordering schemes like AMD, COLAMD and any user generated permutation.

There are other orderings for different purposes. For example, one could order a matrix to ensure that it has no zeros on the diagonal. Otherwise, the Gaussian elimination would fail. Another ordering scheme could reduce the factorization work. KLU employs two such orderings namely an unsymmetric ordering that ensures a zero free diagonal and a symmetric ordering that permutes the matrix into a block upper triangular form (BTF) that restricts factorization to only the diagonal blocks.

One of the key steps in the circuit simulation process is solving sparse linear systems. These systems originate from solving large systems of non linear equations using Newton's method and integrating large stiff systems of ordinary differential equations. These systems are of very high dimensions and a considerable fraction of simulation time is spent on solving these systems. Often the solve phase tends to be a bottleneck in the simulation process. Hence high performance sparse solvers that optimize memory usage and solution time are critical components of circuit simulation software. Some of the popular solvers in use in circuit simulation tools are Sparse1.3 and SuperLU. Sparse1.3 is used in SPICE circuit simulation package and SuperLU uses a supernodal factorization algorithm. Experimental results of KLU indicate that it is 1000 times faster than Sparse1.3 and 1.5 to 3 times faster than SuperLU.

Circuit matrices show some unique properties. They have a nearly zero free diagonal. They have a roughly symmetric pattern but have unsymmetric values. They are highly sparse and often have a few dense rows and columns. These dense rows/columns arise from voltage sources and current sources in the circuit. Circuit matrices show good amenability to BTF ordering. Though the nonzero pattern of original matrix is unsymmetric, the nonzero pattern of blocks produced by BTF

ordering tend to be symmetric. Since circuit matrices are extremely sparse, sparse matrix algorithms such as SuperLU [6] and UMFPACK [7, 8] that employ dense BLAS kernels are often inappropriate. Another unique characteristic of circuit matrices is that employing a good ordering strategy keeps the L and U factors sparse. However as we will see in experimental results, typical ordering strategies can lead to high fill-in.

In circuit simulation problems, typically the circuit matrix template is generated once and the numerical values of the matrix alone change. In other words, the nonzero pattern of the matrix does not change. This implies that we need to order and factor the matrix once to generate the ordering permutations and the nonzero patterns of L and U factors. For all subsequent matrices, we can use the same information and need only to recompute the numerical values of the L and U factors. This process of skipping analysis and factor phases is called refactorization.Refactorization leads to a significant reduction in run time.

Because of the unique characteristics of circuit matrices and their amenability to BTF ordering, KLU is a method well-suited to circuit simulation problems. KLU has been implemented in the C language. It offers a set of API for the analysis phase, factor phase, solve phase and refactor phase. It also offers the ability to solve upto four right hand sides in a single solve step. In addition, it offers transpose solve, conjugate transpose solve features and diagnostic tools like pivot growth estimator and condition number estimator. It also offers a MATLAB interface for the API so that KLU can be used from within the MATLAB environment.

CHAPTER 2
THEORY: SPARSE LU

2.1 Dense LU

Consider the problem of solving the linear system of n equations in n unknowns:

$$\begin{aligned} a_{11}x_1 + a_{12}x_2 + ... + a_{1n}x_n &= b_1 \\ a_{21}x_1 + a_{22}x_2 + ... + a_{2n}x_n &= b_2 \\ &\vdots \\ a_{n1}x_1 + a_{n2}x_2 + ... + a_{nn}x_n &= b_n \end{aligned} \tag{2–1}$$

or, in matrix notation,

$$\begin{bmatrix} a_{11} & a_{12} & \cdots & a_{1n} \\ a_{21} & a_{22} & \cdots & a_{2n} \\ \vdots & & & \\ a_{n1} & a_{n2} & \cdots & a_{nn} \end{bmatrix} * \begin{bmatrix} x_1 \\ x_2 \\ \vdots \\ x_n \end{bmatrix} = \begin{bmatrix} b_1 \\ b_2 \\ \vdots \\ b_n \end{bmatrix} \tag{2–2}$$

$$Ax = b$$

where $A = (a_{ij})$, $x = (x_1, x_2, ..., x_n)^T$ and $b = (b_1, ..., b_n)^T$. A well-known approach to solving this equation is Gaussian elimination. Gaussian elimination consists of a series of eliminations of unknowns x_i from the original system. Let us briefly review the elimination process. In the first step, the first equation of 2–1 is multiplied by $-\frac{a_{21}}{a_{11}}, -\frac{a_{31}}{a_{11}}, ... - \frac{a_{n1}}{a_{11}}$ and added with the second through n^{th} equation of 2–1 respectively. This would eliminate x_1 from second through the nth equations. After

the first step, the 2–2 would become

$$\begin{bmatrix} a_{11} & a_{12} & \cdots & a_{1n} \\ 0 & a_{22}^{(1)} & \cdots & a_{2n}^{(1)} \\ \vdots & & & \\ 0 & a_{n2}^{(1)} & \cdots & a_{nn}^{(1)} \end{bmatrix} * \begin{bmatrix} x_1 \\ x_2 \\ \vdots \\ x_n \end{bmatrix} = \begin{bmatrix} b_1 \\ b_2^{(1)} \\ \vdots \\ b_n^{(1)} \end{bmatrix} \tag{2–3}$$

where $a_{22}^{(1)} = a_{22} - \frac{a_{21}}{a_{11}} * a_{12}$, $a_32^{(1)} = a_{32} - \frac{a_{31}}{a_{11}} * a_{12}$ and so on. In the second step, x_2 will be eliminated by a similar process of computing multipliers and adding the multiplied second equation with the third through n^{th} equations. After n-1 eliminations, the matrix A is transformed to an upper triangular matrix U. The upper triangular system is then solved by back-substitution.

An equivalent interpretation of this elimination process is that we have factorized A into a lower triangular matrix L and an upper triangular matrix U where

$$L = \begin{bmatrix} 1 & 0 & 0 & \cdots & 0 \\ \frac{a_{21}}{a_{11}} & 1 & 0 & \cdots & 0 \\ \frac{a_{31}}{a_{11}} & \frac{a_{32}^{(1)}}{a_{22}^{(1)}} & 1 & \cdots & 0 \\ \vdots & & & & \\ \frac{a_{n1}}{a_{11}} & \frac{a_{n2}^{(1)}}{a_{22}^{(1)}} & \frac{a_{n3}^{(2)}}{a_{33}^{(2)}} & \cdots & 1 \end{bmatrix} \tag{2–4}$$

and

$$U = \begin{bmatrix} a_{11} & a_{12} & a_{13} & \cdots & a_{1n} \\ 0 & a_{22}^{(1)} & a_23^{(1)} & \cdots & a_{2n}^{(1)} \\ 0 & 0 & a_33^{(2)} & \cdots & a_{3n}^{(2)} \\ \vdots & & & & \\ 0 & 0 & 0 & \cdots & a_{nn}^{(n-1)} \end{bmatrix} \tag{2–5}$$

The column k of the lower triangular matrix L consists of the multipliers obtained during step k of the elimination process, with their sign negated.

Mathematically, $Ax = b$ can be rewritten as

$$\begin{aligned} (LU)x &= b \\ L(Ux) &= b \end{aligned} \tag{2–6}$$

Substituting $Ux = y$ in 2–6, we have

$$Ly = b \tag{2–7}$$

$$Ux = y \tag{2–8}$$

By solving these two lower triangular systems, we find the solution to the actual system.

The reason for triangularizing the system is to avoid finding the inverse of the original coefficient matrix A. Inverse finding is atleast thrice as expensive as Gaussian elimination in the dense case and often leads to more inaccuracies.

2.2 Sparse LU

A sparse matrix is defined as one that has few nonzeros in it. The quantification of the adjective 'few' is not specified. The decision as to what kind of algorithm to use (sparse or dense) depends on the fill-in properties of the matrices. However, sparse matrices typically have $O(n)$ nonzero entries. Dense matrices are typically represented by a two dimensional array.The zeros of a sparse matrix should not be stored if we want to save memory. This fact makes a two dimensional array unsuitable for representing sparse matrices. Sparse matrices are represented with a different kind of data structure. They can be represented in two different data structures viz.column compressed form or row compressed form.

A column compressed form consists of three vectors Ap, Ai and Ax. Ap consists of column pointers. It is of length $n+1$. The start of column k of the input matrix is given by Ap [k].

Ai consists of row indices of the elements. This is a zero based data structure with row indices in the interval [0,n). Ax consists of the actual numerical values of the elements.

Thus the elements of a column k of the matrix are held in

Ax [Ap [k]...Ap [k+1]). The corresponding row indices are held in

Ai [Ap [k]...Ap[k+1]).

Equivalently, a row compressed format stores a row pointer vector Ap, a column indices vector Ai and a value vector Ax. For example, the matrix

$$\begin{bmatrix} 5 & 0 & 0 \\ 4 & 2 & 0 \\ 3 & 1 & 8 \end{bmatrix}$$

when represented in column compressed format will be

Ap: 0 3 5 6

Ai: 0 1 2 1 2 2

Ax: 5 4 3 2 1 8

and when represented in row compressed format will be

Ap: 0 1 3 6

Ai: 0 0 1 0 1 2

Ax: 5 4 2 3 1 8

Let nnz represent the number of nonzeros in a matrix of dimension $n * n$. Then in a dense matrix representation, we will need n^2 memory to represent the matrix. In sparse matrix representation, we reduce it to $O(n + nnz)$ and typically $nnz \ll n^2$.

2.3 Left Looking Gaussian Elimination

Let us derive a left looking version of Gaussian elimination. Let an input matrix A of order $n * n$ be represented as a product of two triangular matrices L and U.

Let

$$\begin{bmatrix} A_{11} & \mathbf{a_{12}} & A_{13} \\ \mathbf{a_{21}} & a_{22} & \mathbf{a_{23}} \\ A_{31} & \mathbf{a_{32}} & A_{33} \end{bmatrix} = \begin{bmatrix} L_{11} & 0 & 0 \\ \mathbf{l_{21}} & 1 & 0 \\ L_{31} & \mathbf{l_{32}} & L_{33} \end{bmatrix} * \begin{bmatrix} U_{11} & \mathbf{u_{12}} & U_{13} \\ 0 & u_{22} & \mathbf{u_{23}} \\ 0 & 0 & U_{33} \end{bmatrix} \tag{2–9}$$

where A_{ij} is a block, $\mathbf{a_{ij}}$ is a vector and a_{ij} is a scalar. The dimensions of different elements in the matrices are as follows:

A_{11}, L_{11}, U_{11} are $k * k$ blocks

$\mathbf{a_{12}}, \mathbf{u_{12}}$ are $k * 1$ vectors

A_{13}, U_{13} are $k * n - (k + 1)$ blocks

$\mathbf{a_{21}}, \mathbf{l_{21}}$ are $1 * k$ row vectors

a_{22}, u_{22} are scalars

$\mathbf{a_{23}}, \mathbf{u_{23}}$ are $1 * n - (k + 1)$ row vectors

A_{31}, L_{31} are $n - (k + 1) * k$ blocks

$\mathbf{a_{32}}, \mathbf{l_{32}}$ are $n - (k + 1) * 1$ vectors

A_{33}, L_{33}, U_{33} are $n - (k + 1) * n - (k + 1)$ blocks.

From (2–9), we can arrive at the following set of equations.

$$L_{11} * U_{11} = A_{11} \tag{2–10}$$

$$L_{11} * \mathbf{u_{12}} = \mathbf{a_{12}} \tag{2–11}$$

$$L_{11} * U_{13} = A_{13} \tag{2–12}$$

$$\mathbf{l_{21}} * U_{11} = \mathbf{a_{21}} \tag{2–13}$$

$$\mathbf{l_{21}} * \mathbf{u_{12}} + u_{22} = a_{22} \tag{2–14}$$

$$\mathbf{l_{21}} * U_{13} + \mathbf{u_{23}} = \mathbf{a_{23}} \tag{2–15}$$

$$L_{31} * U_{11} = A_{31} \tag{2–16}$$

$$L_{31} * \mathbf{u_{12}} + \mathbf{l_{32}} * u_{22} = \mathbf{a_{32}} \tag{2–17}$$

$$L_{31} * U_{13} + \mathbf{l_{32}} * \mathbf{u_{23}} + L_{33} * U_{33} = A_{33} \tag{2–18}$$

From (2–11), (2–14) and (2–17), we can compute the 2nd column of L and U, assuming we have already computed L_{11}, $\mathbf{l_{21}}$ and L_{31}. We first solve the lower triangular system (2–11) for $\mathbf{u_{12}}$. Then, we solve for u_{22} using (2–14) by computing the sparse dot product

$$u_{22} = a_{22} - \mathbf{l_{21}} * \mathbf{u_{12}} \tag{2–19}$$

Finally we solve (2–17) for $\mathbf{l_{32}}$ as

$$\mathbf{l_{32}} = \frac{1}{u_{22}}(\mathbf{a_{32}} - L_{31} * \mathbf{u_{12}}) \tag{2–20}$$

This step of computing the 2nd column of L and U can be considered equivalent to solving a lower triangular system as follows:

$$\begin{bmatrix} L_{11} & 0 & 0 \\ \mathbf{l_{21}} & 1 & 0 \\ L_{31} & 0 & 1 \end{bmatrix} * \begin{bmatrix} \mathbf{u_{12}} \\ u_{22} \\ \mathbf{l_{32}} * u_{22} \end{bmatrix} = \begin{bmatrix} \mathbf{a_{12}} \\ a_{22} \\ \mathbf{a_{32}} \end{bmatrix} \tag{2–21}$$

This mechanism of computing column k of L and U by solving a lower triangular system $L * x = b$ is the key step in a left-looking factorization algorithm. As we will see later, Gilbert-Peierls' algorithm revolves around solving this lower triangular system. The algorithm is called a left-looking algorithm since column k of L and U are computed by using the already computed columns 1...k-1 of L. In other words, to compute column k of L and U, one looks only at the already computed columns 1...k-1 in L, that are to the left of the currently computed column k.

2.4 Gilbert-Peierls' Algorithm

Gilbert-Peierls' [9] proposed an algorithm for Gaussian elimination with partial pivoting in time proportional to the flop count of the elimination to factor an arbitrary non singular sparse matrix A as $PA = LU$. If $flops(LU)$ is the number

of nonzero multiplications performed when multiplying two matrices L and U, then Gaussian elimination uses exactly $flops(LU)$ multiplications and divisions to factor a matrix A into L and U. Given an input matrix and assuming no partial pivoting, it is possible to predict the nonzero pattern of its factors. However with partial pivoting, it is not possible to predict the exact nonzero pattern of the factors before hand. Finding an upper bound is possible, but the bound can be very loose [10]. Note that computing the nonzero pattern of L and U is a necessary part of Gaussian elimination involving sparse matrices since we do not use two dimensional arrays for representing them but sparse data structures. Gilbert-Peierls' algorithm aims at computing the nonzero pattern of the factors and the numerical values in a total time proportional to $O(flops(LU))$.

It consists of two stages for determining every column of L and U. The first stage is a symbolic analysis stage that computes the nonzero pattern of the column k of the factors. The second stage is the numerical factorization stage that involves solving the lower triangular system $Lx = b$, that we discussed in the section above.

2.4.1 Symbolic Analysis

A sparse Gaussian elimination algorithm with partial pivoting cannot know the exact nonzero structure of the factors ahead of all numerical computation, simply because partial pivoting at column k can introduce new nonzeros in columns $k+1 \cdots n$. Solving $Lx = b$ must be done in time proportional to the number of flops performed. Consider a simple column-oriented algorithm in MATLAB notation for solving $Lx = b$ as follows:

```
x = b
for j = 1:n
    if x(j) ~= 0
        x(j+1:n) = x(j+1:n) - L(j+1:n,j) * x(j)
    end
```

```
end
```

The above algorithm takes time $O(n + number\ \ of\ \ flops\ \ performed)$. The $O(n)$ term looks harmless, but $Lx = b$ is solved n times in the factorization of $A = LU$, leading to an unacceptable $O(n^2)$ term in the work to factorize A into L times U. To remove the $O(n)$ term, we must replace the algorithm with

```
x = b
for each j for which x(j) ~= 0
    x(j+1:n) = x(j+1:n) - L(j+1:n, j) * x(j)
end
```

This would reduce the $O(n)$ term to $O(\eta(b))$, where $\eta(b)$ is the number of nonzeros in b. Note that b is a column of the input matrix A. Thus to solve $Lx = b$, we need to know the nonzero pattern of x before we compute x itself. Symbolic analysis helps us determine the nonzero pattern of x.

Let us say we are computing column k of L and U. Let $G = G(L_k)$ be the directed graph of L with $k - 1$ vertices representing the already computed $k - 1$ columns. $G(L_k)$ has an edge $j \rightarrow i$ iff $l_{ij} \neq 0$. Let $\beta = \{i|b_i \neq 0\}$ and $X = \{i|x_i \neq 0\}$ Now the elements of X is given by

$$X = Reach_{G(L)}(\beta) \tag{2–22}$$

The nonzero pattern of X is computed by the determining the vertices that are reachable from the vertices of the set β. The reachability problem can be solved using a classical depth first search in $G(L_k)$ from the vertices of the set β. If $b_j \neq 0$, then $x_j \neq 0$. In addition if $L_{ij} \neq 0$, then $x_i \neq 0$ even if $b_i = 0$. This is because a $L_{ij} * x_j$ contributes to a nonzero in the equation when we solve for x_i. During the depth first search, Gilbert-Peierls' algorithm computes the topological order of X. This topological ordering is useful for eliminating unknowns in the Numerical factorization step.

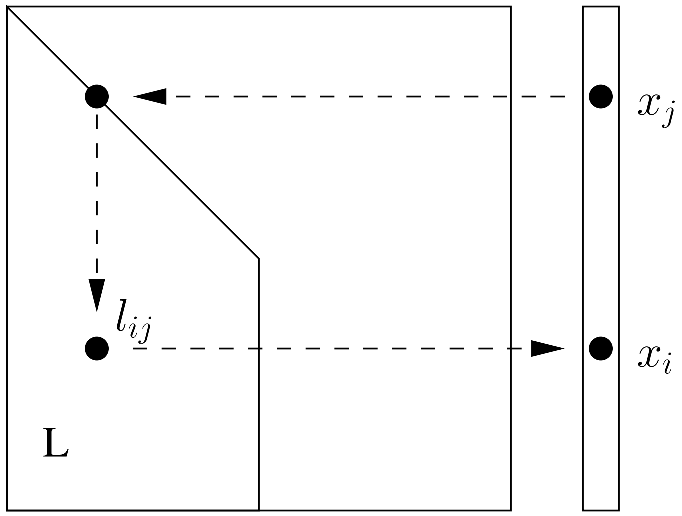

Figure 2–1: Nonzero pattern of x when solving Lx = b

The row indices vector L_i of columns $1 \cdots k-1$ of L represents the adjacency list of the graph $G(L_k)$. The depth first search takes time proportional to the number of vertices examined plus the number of edges traversed.

2.4.2 Numerical Factorization

Numerical factorization consists of solving the system (2–21) for each column k of L and U. Normally we would solve for the unknowns in (2–21) in the increasing order of the row index. The row indices/nonzero pattern computed by depth first search are not necessarily in increasing order. Sorting the indices would increase the time complexity above our $O(flops(LU))$ goal. However, the requirement of eliminating unknowns in increasing order can be relaxed to a topological order of the row indices. An unknown x_i can be computed, once all the unknowns x_j of which it is dependent on are computed. This is obvious when we write the equations comprising a lower triangular solve. Theoretically, the unknowns can be solved in any topological order. The depth first search algorithm gives one such topological order which is sufficient for our case. In our example, the depth first search would have finished exploring vertex i before it finishes exploring vertices j.

Hence a topological order given by depth first search would have j appearing before i. This is exactly what we need.

Gilbert-Peierls' algorithm starts with an identity L matrix. The entire left looking algorithm can be summarized in MATLAB notation as follows:

```
L = I
for k = 1:n
    x = L \ A(:,k)
    %(partial pivoting on x can be done here)
    U(1:k,k) = x(1:k)
    L(k:n,k) = x(k:n) / U(k,k)
end
```

where $x = L\backslash b$ denotes the solution of a sparse lower triangular system. In this case, b is the kth column of A. The total time complexity of Gilbert-Peierls' algorithm is $O(\eta(A) + flops(LU))$. $\eta(A)$ is the number of nonzeros in the matrix A and $flops(LU)$ is the flop count of the product of the matrices L and U. Typically $flops(LU)$ dominates the complexity and hence the claim of factorizing in time proportional to the flop count.

2.5 Maximum Transversal

Duff [11, 12] proposed an algorithm for determining the maximum transversal of a directed graph.The purpose of the algorithm is to find a row permutation that minimizes the zeros on the diagonal of the matrix. For non singular matrices, the algorithm ensures a zero free diagonal. KLU employs Duff's [11, 12] algorithm to find an unsymmetric permutation of the input matrix to determine a zero-free diagonal. A matrix cannot be permuted to have a zero free diagonal if and only if it is structurally singular. A matrix is structurally singular if there is no permutation of its nonzero pattern that makes it numerically nonsingular.

A transversal is defined as a set of nonzeros, no two of which lie in the same row or column, on the diagonal of the permuted matrix. A transversal of maximum length is the maximum transversal.

Duff's maximum transversal algorithm consists of representing the matrix as a graph with each vertex corresponding to a row in the matrix. An edge $i_k \rightarrow i_{k+1}$ exists in the graph if $A(i_k, j_{k+1})$ is a nonzero and $A(i_{k+1}, j_{k+1})$ is an element in the transversal set. A path between vertices i_0 and i_k would consist of a sequence of nonzeros $(i_0, j_1), (i_1, j_2), \cdots (i_{k-1}, j_k)$ where the current transversal would include $(i_1, j_1), (i_2, j_2), \cdots (i_k, j_k)$. If there is a nonzero in position (i_k, j_{k+1}) and no nonzero in row i_0 or column j_{k+1} is currently on the travseral, it increases the transerval by one by adding the nonzeros $(i_r, j_{r+1}), r = 0, 1, \cdots, k$ to the transversal and removing the nonzeros $(i_r, j_r), r = 1, 2, \cdots, k$ from the transversal. This adding and removing of nonzeros to and from the transversal is called reassignment chain or augmenting path.

A vertex or row is said to be assigned if a nonzero in the row is chosen for the transversal. The process of constructing augmenting paths is done by doing a depth first search from an unassigned row i_0 of the matrix and continue till a vertex i_k is reached where the path terminates because $A(i_k, j_{k+1})$ is a nonzero and column j_{K+1} is unassigned. Then the search backtracks to i_0 adding and removing transversal elements thus constructing an augmenting path.

Duff's maximum transversal transversal algorithm has a worst case time complexity of $O(n\tau)$ where τ is the number of nonzeros in the matrix and n is the order of the matrix. However in practice, the time complexity is close to $O(n + \tau)$.

The maximum transversal problem can be cast as a maximal matching problem on bipartite graphs. This is only to make a comparison. The maximal matching problem is stated as follows.

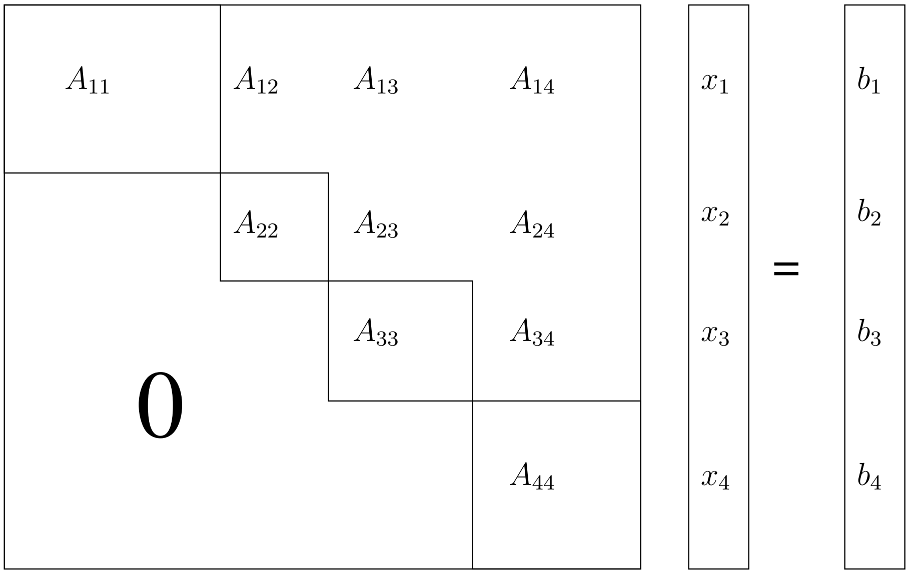

Figure 2–2: A matrix permuted to BTF form

Given an undirected graph $G = (V, E)$, a matching is a subset of the edges $M \subseteq E$ such that for all vertices $v \in V$, at most one edge of M is incident on v. A vertex $v \in V$ is matched if some edge in M is incident on v, otherwise, v is unmatched. A maximal matching is a matching of maximum cardinality, that is a matching M such that for any matching M', we have $|M| \geq |M'|$.

A maximal matching can be built incrementally, by picking an arbitraty edge e in the graph, deleting any edge that is sharing a vertex with e and repeating until the graph is out of edges.

2.6 Block Triangular Form

A block (upper) triangular matrix is similar to a upper triangular matrix except that the diagonals in the former are square blocks instead of scalars. Figure 2–2 shows a matrix permuted to the BTF form.

Converting the input matrix to block triangular form is important in that,

1. The part of the matrix below the block diagonal requires no factorization effort.

2. The diagonal blocks are independent of each other.Only the blocks need to be factorized. For example, in figure 2–2, the subsystem $A_{44}x_4 = b_4$ can be solved independently for x_4 and x_4 can be eliminated from the overall system. The system $A_{33}x_3 = b_3 - A_{34}x_4$ is then solved for x_3 and so on.
3. The off diagonal nonzeros do not contribute to any fill-in.

Finding a symmetric permutation of a matrix to its BTF form is equivalent to finding the strongly connected components of a graph. A strongly connected component of a directed graph $G = (V, E)$ is a maximal set of vertices $C \subseteq V$ such that for every pair of vertices u and v in C, we have both $u \to v$ and $v \to u$. The vertices u and v are reachable from each other.

The Algorithm employed in KLU for symmetric permutation of a matrix to a BTF form, is based on Duff and Reid's [13, 14] algorithm. Duff and Reid provide an implementation for Tarjan's [15] algorithm to determine the strongly connected components of a directed graph. The algorithm has a time complexity of $O(n + \tau)$ where n is the order of the matrix and τ is the number of off diagonal nonzeros in the matrix.

The algorithm essentially consists of doing a depth first search from unvisited nodes in the graph. It uses a stack to keep track of nodes being visited and uses a path of the nodes. When all edges in the path are explored, it generates Strongly connected components from the top of stack.

Duff's algorithm is different from the method proposed by Cormen, Leiserson, Rivest and Stein [16]. They suggest doing a depth first search on G , computing G^T and then running a depth first search on G^T on vertices in the decreasing order of their finish times from the first depth first search (the topological order from the first depth first search).

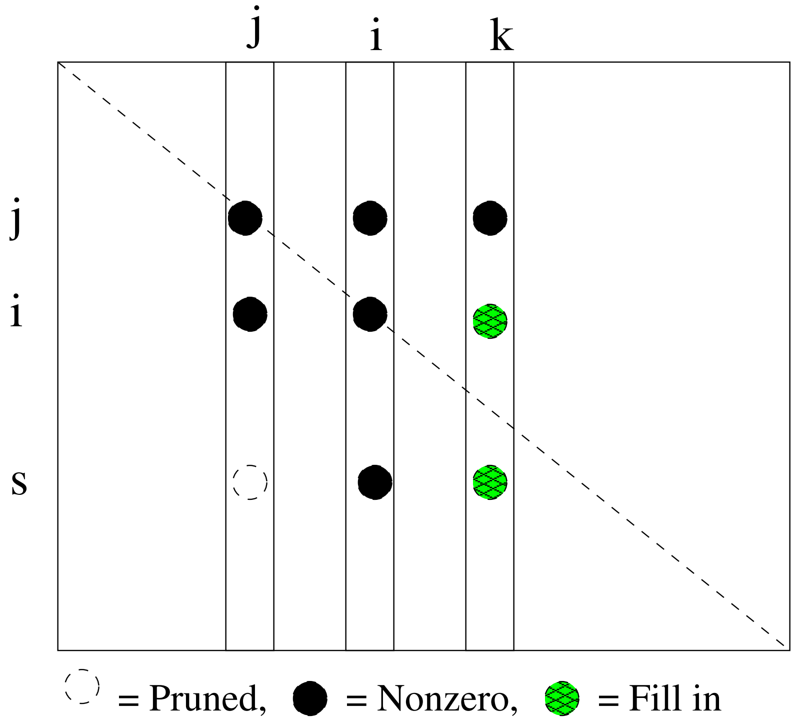

Figure 2–3: A symmetric pruning scenario

2.7 Symmetric Pruning

Eisenstat and Liu [17] proposed a method called Symmetric Pruning to exploit structural symmetry for cutting down the symbolic analysis time. The cost of depth first search can be cut down by pruning unnecessary edges in the graph of $L, G(L)$. The idea is to replace $G(L)$ by a reduced graph of L. Any graph H can be used in place of $G(L)$, provided that $i \rightarrow j$ exists in H iff $i \rightarrow j$ exists in $G(L)$. If A is symmetric, then the symmetric reduction is just the elimination tree.

The symmetric reduction is a subgraph of $G(L)$. It has fewer edges than $G(L)$ and is easier to compute by taking advantage of symmetry in the structure of the factors L and U. Even though the symmetric reduction removes edges, it still preserves the paths between vertices of the original graph.

Figure 2–3 shows a symmetric pruning example.

If $l_{ij} \neq 0, u_{ji} \neq 0$, then we can prune edges $j \rightarrow s$, where $s > i$. The reason behind this is that for any $a_{jk} \neq 0$, a_{sk} will fill in from column j of L for $s > k$.

The just computed column i of L is used to prune earlier columns. Any future depth first search from vertex j will not visit vertex s, since s would have been visited via i already.

Note that every column is pruned only once. KLU employs symmetric pruning to speed up the depth first search in the symbolic analysis stage.

2.8 Ordering

It is a widely used practice to precede the factorization step of a sparse linear system by an ordering phase. The purpose of the ordering is to generate a permutation P, that reduces the fill-in in the factorization phase of PAP^T. A fill-in is defined as a nonzero in a position (i, j) of the factor that was zero in the original matrix. In other words, we have a fill-in if $L_{ij} \neq 0$, where $A_{ij} = 0$.

The permuted matrix created by the ordering PAP^T creates much less fill-in in factorization phase than the unpermuted matrix A. The ordering mechanism typically takes into account only the structure of the input matrix, without considering the numerical values stored in the matrix. Partial pivoting during factorization changes the row permutation P and hence could potentially increase fill-in as opposed to what was estimated by the ordering scheme. We shall see more about pivoting in the following sections.

If the input matrix A is unsymmetric, then the permutation of the matrix $A + A^T$ can be used. Various minimum degree algorithms can be used for ordering. Some of the popular ordering schemes include approximate minimum degree(AMD) [18, 19], column approximate minimum degree(COLAMD) [20, 21] among others. COLAMD orders the matrix AA^T without forming it explicitly.

After permuting an input matrix A into BTF form using the maximum transversal and BTF orderings, KLU attempts to factorize each of the diagonal blocks. It applies the fill reducing ordering algorithm on the block before factorizing it. KLU supports both approximate minimum degree and column approximate

minimum degree. Besides, any given ordering algorithm can be plugged into KLU without much effort. Work is being done on integrating a Nested Dissection ordering strategy into KLU as well.

Of the various ordering schemes, AMD gives best results on circuit matrices. AMD finds a permutation P to reduce fill-in for the Cholesky factorization of PAP^T (of $P(A + A^T)P^T$, if A is unsymmetric). AMD assumes no numerical pivoting. AMD attempts to reduce an optimistic estimate of fill-in.

COLAMD is an unsymmetric ordering scheme, that computes a column permutation Q to reduce fill-in for Cholesky factorization of $(AQ)^T AQ$. COLAMD attempts to reduce a "pessimitic" estimate (upper bound) of fill-in.

Nested Dissection is another ordering scheme that creates permutation such that the input matrix is transformed into block diagonal form with vertex separators. This is a popular ordering scheme. However, it is unsuitable for circuit matrices when applied to the matrix as such. It can be used on the blocks generated by BTF pre-ordering.

The idea behind a minimum degree algorithm is as follows: A structurally symmetric matrix A can be represented by an equivalent undirected graph $G(V, E)$ with vertices corresponding to row/column indices. An edge $i \rightarrow j$ exists in G if $A_{ij} \neq 0$.

Consider the figure 2–4. If the matrix is factorized with vertex 1 as the pivot, then after the first Gaussian elimination step, the matrix would be transformed as in figure 2–5.

This first step of elimination can be considered equivalent to removing node 1 and all its edges from the graph and adding edges to connect all nodes adjacent to 1. In other words, the elimination has created a clique of the nodes adjacent to the eliminated node. Note that there are as many fill-ins in the reduced matrix as there are edges added in the clique formation. In the above example, we have

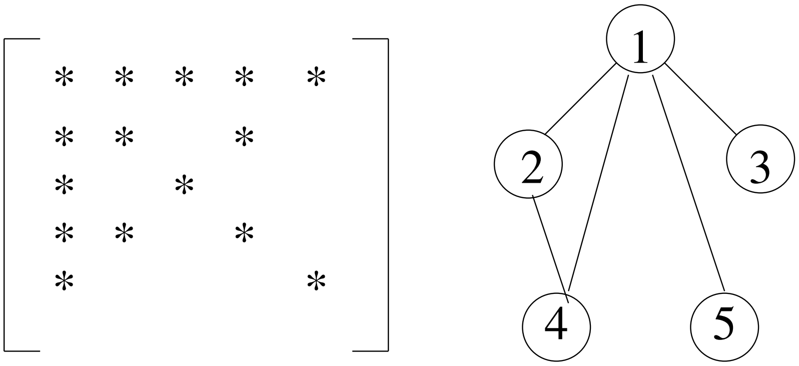

Figure 2–4: A symmetric matrix and its graph representation

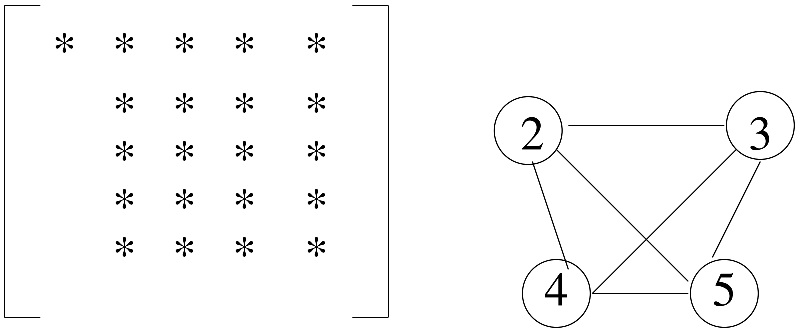

Figure 2–5: The matrix and its graph representation after one step of Gaussian elimination

chosen the wrong node as pivot, since node 1 has the maximum degree. Instead if we had chosen a node with minimum degree say 3 or 5 as pivot, then there would have been zero fill-in after the elimination since both 3 and 5 have degree 1.

This is the key idea in a minimum degree algorithm. It generates a permutation such that a node with minimum degree is eliminated in each step of Gaussian elimination, thus ensuring a minimal fill-in. The algorithm does not examine the numerical values in the node selection process. It could happen that during partial pivoting, a node other than the one suggested by the minimum degree algorithm must be chosen as pivot because of its numerical magnitude. That's exactly the reason why the fill-in estimate produced by the ordering algorithm could be less than that experienced in the factorization phase.

2.9 Pivoting

Gaussian elimination fails when the diagonal element in the input matrix happens to be zero. Consider a simple $2 * 2$ system,

$$A = \begin{bmatrix} 0 & a_{12} \\ a_{21} & a_{22} \end{bmatrix} * \begin{bmatrix} x_1 \\ x_2 \end{bmatrix} = \begin{bmatrix} b_1 \\ b_2 \end{bmatrix} \tag{2–23}$$

When solving the above system, Gaussian elimination computes the multiplier $-a_{21}/a_{11}$ [and multiplies row 1 with this multiplier and adds it to row 2] thus eliminating the coefficient element a_{21} from the matrix. This step obviously would fail, since a_{11} is zero. Now let's see a classical case when the diagonal element is nonzero but close to zero.

$$A = \begin{bmatrix} 0.0001 & 1 \\ 1 & 1 \end{bmatrix} \tag{2–24}$$

The multiplier is $-1/0.0001 = -10^4$. The factors L and U are

$$L = \begin{bmatrix} 1 & 0 \\ 10^4 & 1 \end{bmatrix} U = \begin{bmatrix} 0.0001 & 1 \\ 0 & -10^4 \end{bmatrix} \quad (2\text{–}25)$$

The element u_{22} has the actual value $1 - 10^4$. However assuming a four digit arithmetic, it would be rounded off to -10^4. Note that the product of L and U is

$$L * U = \begin{bmatrix} 0.0001 & 1 \\ 1 & 0 \end{bmatrix} \quad (2\text{–}26)$$

which is different from the original matrix. The reason for this problem is that the multiplier computed is so large that when added with the small element a_{22} with value 1, it obscured the tiny value present in a_{22}.

We can solve these problems with pivoting. In the above two examples, we could interchange rows 1 and 2, to solve the problem. This mechanism of interchanging rows(and columns) and picking a large element as the diagonal, to avoid numerical failures or inaccuracies is called pivoting. To pick a numerically large element as pivot, we could look at the elements in the current column or we could look at the entire submatrix (across both rows and columns). The former is called partial pivoting and the latter is called complete pivoting.

For dense matrices, partial pivoting adds a time complexity of $O(n^2)$ comparisons to Gaussian elimination and complete pivoting adds $O(n^3)$ comparisons. Complete pivoting is expensive and hence is generally avoided, except for special cases. KLU employs partial pivoting with diagonal preference. As long as the diagonal element is atleast a constant threshold times the largest element in the column, we choose the diagonal as the pivot. This constant threshold is called pivot tolerance.

2.10 Scaling

The case where small elements in the matrix get obscured during the elimination process and accuracy of the results gets skewed because of numerical addition

is not completely overcome by the pivoting process. Let us see an example of this case.

Consider the $2 * 2$ system

$$A = \begin{bmatrix} 10 & 10^5 \\ 1 & 1 \end{bmatrix} * \begin{bmatrix} x_1 \\ x_2 \end{bmatrix} = \begin{bmatrix} 10^5 \\ 2 \end{bmatrix} \tag{2–27}$$

When we apply Gaussian elimination with partial pivoting to the above system, the entry a_{11} is largest in the first column and hence would continue to be the pivot.After the first step of elimination assuming a four digit arithmetic, we would have

$$A = \begin{bmatrix} 10 & 10^5 \\ 0 & -10^4 \end{bmatrix} * \begin{bmatrix} x_1 \\ x_2 \end{bmatrix} = \begin{bmatrix} 10^5 \\ -10^4 \end{bmatrix} \tag{2–28}$$

The solution from the above elimination is $x_1 = 1, x_2 = 0$. However the correct solution is close to $x_1 = 1, x_2 = 1$.

If we divide each row of the matrix by the largest element in that row(and the corresponding element in the right hand side as well), prior to Gaussian elimination we would have

$$A = \begin{bmatrix} 10^{-4} & 1 \\ 1 & 1 \end{bmatrix} * \begin{bmatrix} x_1 \\ x_2 \end{bmatrix} = \begin{bmatrix} 1 \\ 2 \end{bmatrix} \tag{2–29}$$

Now if we apply partial pivoting we would have,

$$A = \begin{bmatrix} 1 & 1 \\ 10^{-4} & 1 \end{bmatrix} * \begin{bmatrix} x_1 \\ x_2 \end{bmatrix} = \begin{bmatrix} 2 \\ 1 \end{bmatrix} \tag{2–30}$$

And after an elimination step, the result would be

$$A = \begin{bmatrix} 1 & 1 \\ 0 & 1 - 10^{-4} \end{bmatrix} * \begin{bmatrix} x_1 \\ x_2 \end{bmatrix} = \begin{bmatrix} 2 \\ 1 - 10^{-4} \end{bmatrix} \tag{2–31}$$

which yields the correct solution $x_1 = 1, x_2 = 1$. The process of balancing out the numerical enormity or obscurity on each row or column is termed as scaling. In the above example, we have scaled with respect to the maximum value in a row which is row scaling. Another variant would be to scale with respect to the sum of absolute values of all elements across a row.

In column scaling, we would scale with respect to the maximum value in a column or the sum of absolute values of all elements in a cloumn.

Row scaling can be considered equivalent to finding an invertible diagonal matrix D_1 such that all the rows in the matrix $D^{-1}A$ have equally large numerical values.

Once we have such a D_1, the solution of the original system $Ax = b$ is equivalent to solving the system $\tilde{A}x = \tilde{b}$ where $\tilde{A} = D^{-1}A$ and $\tilde{b} = D^{-1}b$.

Equilibration is another popular term used for scaling.

In KLU, the diagonal elements of the diagonal matrix D_1 are either the largest elements in the rows of the original matrix or the sum of the absolute values of the elements in the rows. Besides scaling can be turned off as well, if the simulation environment does not need scaling. Scaling though it offers better numerical results when solving systems, is not mandatory. Its usage depends on the data values that constitute the system and if the values are already balanced, scaling might not be necessary.

2.11 Growth Factor

Pivot growth factor is a key diagnostic estimate in determining the stability of Gaussian elimination. Stability of Numerical Algorithms is an important factor in determining the accuracy of the solution. Study of stability is done by a process

called Roundoff Error analysis. Roundoff error analysis comprises two sub types called Forward error analysis and Backward error analysis. If the computed solution x̃ is close to the exact solution x, then the algorithm is said to be Forward stable. If the algorithm computes an exact solution to a nearby problem, then the algorithm is said to be Backward stable.Backward stability is the most widely used technique in studying stability of systems. Often the data generated for solving systems have impurity in them or they are distorted by a small amount. Under such circumstances we are interested that the algorithm produce an exact solution to this nearby problem and hence the relevance of backward stability assumes significance.

Pivot growth factor is formally defined as

$$\rho = \frac{\overset{max}{k} \; \overset{max}{ij} \; |a_{ij}^{(k)}|}{\overset{max}{ij} \; |a_{ij}|} \tag{2–32}$$

where $a_{ij}^{(k)}$ is an entry in the reduced matrix $A^{(k)}$ after the kth elimination step. From (2–32), we find that if the entries of the reduced matrix grow arbitrarily, we would have a high growth factor. This arbitrary growth would again lead to inaccuracies in the results. Consider the following $2 * 2$ system.

$$A = \begin{bmatrix} 10^{-4} & 1 \\ 1 & 1 \end{bmatrix} * \begin{bmatrix} x_1 \\ x_2 \end{bmatrix} = \begin{bmatrix} 1 \\ 2 \end{bmatrix} \tag{2–33}$$

After one step of Gaussian elimination assuming four digit arithmetic, we would have the reduced system

$$A = \begin{bmatrix} 10^{-4} & 1 \\ 0 & 1 - 10^{4} \end{bmatrix} * \begin{bmatrix} x_1 \\ x_2 \end{bmatrix} = \begin{bmatrix} 1 \\ 2 - 10^{4} \end{bmatrix} \tag{2–34}$$

Solving the system yields $x_1 = 0, x_2 = 1$ which is different from the actual solution $x_1 = 1, x_2 = 1$.The pivot growth factor of the above system is

$\rho = \frac{max(1,10^4)}{1} = 10^4$

Thus a large pivot growth clearly indicates the inaccuracy in the result. Partial pivoting generally avoids large growth factors. In the above example, if we had applying partial pivoting, we would have got the correct results. But this is not assured and there are cases where partial pivoting might not result in an acceptable growth factor. This necessitates the estimation of the growth factor as a diagnostic tool to detect cases where Gaussian elimination could be unstable.

Pivot growth factor is calculated usually in terms of its reciprocal, to avoid numerical overflow problems when the value is very large. (2–32) is a harder to compute equation since it involves calculating the maximum of reduced matrix after every step of elimination. The other definitions of reciprocal growth factor that are easy to compute are as follows:

$$\frac{1}{\rho} = \overset{min}{j} \frac{(\overset{max}{i} |a_{ij}|)}{(\overset{max}{i} |u_{ij}|)} \tag{2–35}$$

$$\frac{1}{\rho} = \frac{(\overset{max}{ij} |a_{ij}|)}{(\overset{max}{ij} |u_{ij}|)} \tag{2–36}$$

Equation (2–35) is the definition implemented in KLU and it is a column scaling invariant. It helps unmask a large pivot growth that could be totally masked because of column scaling.

2.12 Condition Number

Growth factor is a key estimate in determining the stability of the algorithm. Condition number is a key estimate in determining the amenability or conditioning of a given problem. It is not guaranteed that a highly stable algorithm can yield accurate results for all problems it can solve. The conditioning of the problem has a dominant effect on the accuracy of the solution.

Note that while stability deals with the algorithm, conditioning deals with the problem itself. In practical applications like circuit simulation, the data of

a problem come from experimental observations. Typically such data have a factor of error or impurities or noise associated with them. Roundoff errors and discretization errors also contribute to impurities in the data. Conditioning of a problem deals with determining how the solution of the problem changes in the presence of impurities.

The preceding discussion shows that one often deals with solving problems not with the original data but that with perturbed data. The analysis of effect of perturbation of the problem on the solution is called Perturbation analysis. It helps in determining whether a given problem produces a little or huge variation in solution when perturbed. Let us see what we mean by well or ill conditioned problems.

A problem is said to be **ill conditioned** if a small relative error in data leads to a large relative error in solution irrespective of the algorithm employed. A problem is said to be **well conditioned** if a small relative error in data does not lead to a large relative error in solution.

Accuracy of the computed solution is of primary importance in numerical analysis. Stability of the algorithm and the Conditioning of the given problem are the two factors that directly determine accuracy.A highly stable algorithm well armored with scaling, partial pivoting and other concepts cannot be guaranteed to yield an accurate solution to an ill-conditioned problem.

A backward stable algorithm applied to a well-conditioned problem should yield a solution close to the exact solution. This follows from the definitions of backward stability and well-conditioning, where backward stability assures exact solution to a nearby problem and well-conditioned problem assures that the computed solution to perturbed data is relatively close to the exact solution of the actual problem.

Mathematically, let X be some problem. Let X(d) be the solution to the problem for some input d. Let δd denote a small perturbation in the input d. Now if the relative error in the solution

$$\frac{|X(d+\delta d) - X(d)|}{|X(d)|}$$

exceeds the relative error in the input

$$\frac{|\delta d|}{|d|}$$

then the problem is ill conditioned and well conditioned otherwise.

Condition number is a measure of the conditioning of the problem. It shows whether a problem is well or ill conditioned.For the linear system problems of the form $Ax = b$, the condition number is defined as

$$Cond(A) = \|A\|\|A^{-1}\| \tag{2–37}$$

Equation (2–37) is arrived at by theory that deals with perturbations either in the input matrix A or the right hand side b or both the matrix and right hand side. Equation (2–37) can be defined with respect to any norm viz. 1, 2 or ∞. The system $Ax = b$ is said to be ill-conditioned if the condition number from (2–37) is quite large. Otherwise it is said to be well-conditioned.

A naive way to compute the condition number would be to compute the inverse of the matrix, compute the norm of the matrix and its inverse and compute the product. However, computing the inverse is atleast thrice as expensive as solving the linear system $Ax = b$ and hence should be avoided.

Hager [22] developed a method for estimating the 1-norm of $\|A^{-1}\|$ and the corresponding 1-norm condition number. Hager proposed an optimization approach for estimating $\|A^{-1}\|_1$. The 1-norm of a matrix is formally defined as

$$\|A\|_1 = max\frac{\|Ax\|_1}{\|x\|_1} \tag{2–38}$$

Hager's algorithm can be briefly described as follows: For $A \in R^{n*n}$, a convex function is defined as

$$F(x) = \|Ax\|_1 \tag{2–39}$$

over the convex set

$$S = \{x \in R^n : \|x\|_1 \leq 1\}$$

Then $\|A\|_1$ is the global maximum of (2–39).

The algorithm involves computing Ax and $A^T x$ or computing matrix-vector products. When we want to compute $\|A^{-1}\|_1$, it involves computing $A^{-1}x$ and $(A^{-1})^T x$ which is equivalent to solving $Ax = b$ and $A^T x = b$. We can use KLU to efficiently solve these systems.

Higham [23] presents refinements to Hager's algorithm and restricts the number of iterations to five. Higham further presents a simple device and using the higher of the estimates from this device and Hager's algorithm to ensure the esimate is large enough. This device involves solving the linear system $Ax = b$ where

$$b_i = (-1)^{i+1}(1 + \frac{i-1}{n-1}), i = 1, 2, ...n$$

The final estimate is chosen as the maximum from Hager's algorithm and $\frac{2\|x\|_1}{3n}$.

KLU's condition number estimator is based on Higham's refinement of Hager's algorithm.

2.13 Depth First Search

As we discussed earlier, the nonzero pattern of the kth column of L is determined by the Reachability of the row-indices of kth column of A in the graph of L.

The reachability is determined by a depth-first search traversal of the graph of L. The topological order for elimination of variables when solving the lower triangular system $Lx = b$ is also determined by the depth-first search traversal. A classical depth first search algorithm is a recursive one. One of the major problems in a recursive implementation of depth-first search is Stack overflow. Each process is allocated a stack space upon execution. When there is a high number of recursive calls, the stack space is exhausted and the process terminates abruptly. This is a definite possibility in the context of our depth-first search algorithm when we have a dense column of a matrix of a very high dimension.

The solution to stack overflow caused by recursion is to replace recursion by iteration. With an iterative or non-recursive function, the entire depth first search happens in a single function stack. The iterative solution uses an array of row indices called pstack. When descending to an adjacent node during the search, the row index of the next adjacent node is stored in the pstack at the position(row/column index) corresponding to the current node. When the search returns to the current node, we know that we next need to descend into the node stored in the pstack at the position corresponding to the current node. Using this extra O(n) memory, the iterative version completes the depth first search in a single function stack.

This is an important improvement from the recursive version since it avoids the stack overflow problem that would have been a bottleneck when solving high dimension systems.

2.14 Memory Fragmentation

The data structures for L and U are the ones used to represent sparse matrices. These comprise 3 vectors.

1. Vector of column pointers
2. Vector of row indices

3. Vector of numerical values

There are overall, six vectors needed for the two matrices L and U. Of these, the two vectors of column pointers are of pre-known size namely the size of a block. The remaining four vectors of row indices and numerical values depend on the fill-in estimated by AMD. However AMD gives an optimistic estimate of fill-in.Hence we need to dynamically grow memory for these vectors during the factorization phase if we determine that the fill-in is higher than estimated. The partial pivoting strategy can alter the row ordering determined by AMD and hence is another source of higher fill-in than the estimate from AMD.

Dynamically growing these four vectors suffers from the problem of external memory fragmentation. In external fragmentation, free memory is scattered in the memory space. A call for more memory fails because of non-availability of contiguous free memory space. If the scattered free memory areas were contiguous, the memory request would have succeeded. In the context of our problem, the memory request to grow the four vectors could either fail if we run into external fragmentation or succeed when there is enough free space available.

When we reallocate or grow memory, there are two types of success cases. In the first case called cheap reallocation, there is enough free memory space abutting the four vectors. Here the memory occupied by a vector is just extended or its end boundary is increased. The start boundary remains the same. In the second case called costly reallocation, there is not enough free memory space abutting a vector. Hence a fresh memory is allocated in another region for the new size of vector and the contents are copied from old location. Finally the old location is freed.

With four vectors to grow, there is a failure case because of external fragmentation and a costly success case because of costly reallocation. To reduce the failure case and avoid the costly success case, we have coalesced the four vectors into a single vector. This new data structure is byte aligned on double boundary. For

every column of L and U, the vector contains the row indices and numerical values of L , followed by the row indices and numerical values of U. Multiple integer row indices are stored in a single double location . The actual number of integers that can be stored in a double location varies with platform and is determined dynamically. The common technique of using integer pointer to point to location aligned on double boundary, is employed to retrieve or save the row indices.

In addition to this coalesced data structure containing the row indices and numerical values, two more length vectors of size n are needed to contain the length of each column of L and U. These length vectors are preallocated once and need not be grown dynamically.

Some Memory management schemes never do cheap reallocation. In such schemes, the new data structure serves to reduce external fragmentation only.

2.15 Complex Number Support

KLU supports complex matrices and complex right hand sides. KLU also supports solving the transpose system $A^T x = b$ for real matrices and solving the conjugate transpose system $A^H x = b$ for complex matrices. Initially it relied on the C99 language support for complex numbers. However the C99 specification is not supported across operating systems. For example, earlier versions of Sun Solaris do not support C99. To avoid these compatibility issues, KLU no longer relies on C99 and has its own complex arithmetic implementation.

2.16 Parallelism in KLU

When solving a system $Ax = b$ using KLU, we use BTF pre-ordering to convert A into a block upper triangular form. We apply AMD on each block and factorize each block one after the other serially. Alternatively, nested dissection can be applied to each block. Nested dissection ordering converts a block to a doubly bordered block diagonal form. A doubly bordered block diagonal form is similar to a block upper triangular form but has nonzeros on the sub diagonal region. These

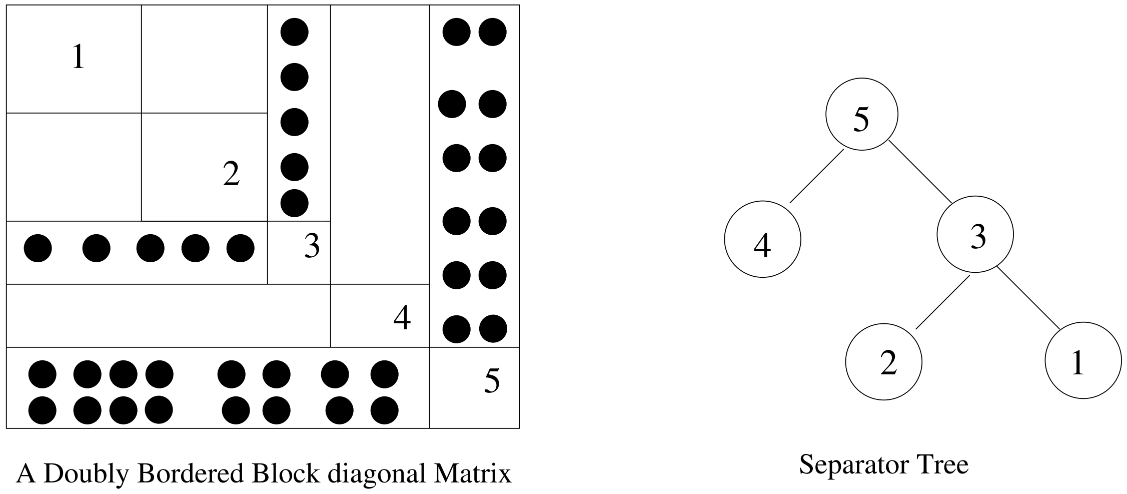

Figure 2–6: A doubly bordered block diagonal matrix and its corresponding vertex separator tree

nonzeros form a horizontal strip resembling a border. Similarly the nonzeros in the region above the diagonal form a corresponding vertical strip.

The doubly bordered block diagonal form can be thought of as a separator tree. Factorization of the block then involves a post-order traversal of the separator tree. The nodes in the separator tree can be factorized in parallel. The factorization of a node would additionally involve computing the schur complement of its parent and of its ancestors in the tree. Once all the children of a node have updated its schur complement, the node is ready to be factorized and it inturn computes the schur complement of its parent and its ancestors. The factorization and computation of schur complement is done in a post-order traversal fashion and the process stops at the root.

Parallelism can help in reducing the factorization time. It gains importance in the context of multi processor systems. Work is being done to enable parallelism in KLU.

CHAPTER 3
CIRCUIT SIMULATION: APPLICATION OF KLU

The KLU algorithm comprises the following steps:

1. Unsymmetric Permutation to block upper triangular form. This consists of two steps.
   (a) unsymmetric permutation to ensure a zero free diagonal using maximum transversal.
   (b) symmetric permutation to block upper triangular form by finding the strongly connected components of the graph.
2. Symmetric permutation of each block(say A) using AMD on $A + A^T$ or an unsymmetric permutation of each block using COLAMD on AA^T. These permutations are fill-in reducing orderings on each block.
3. Factorization of each scaled block using Gilbert-Peierls' left looking algorithm with partial pivoting.
4. Solve the system using block-back substitution and account for the off-diagonal entries. The solution is re-permuted to bring it back to original order.

Let us first derive the final system that we need to solve taking into account, the different permutations, scaling and pivoting. The original system to solve is

$$Ax = b \tag{3–1}$$

Let R be the diagonal matrix with the scale factors for each row. Applying scaling, we have

$$RAx = Rb \tag{3–2}$$

Let P' and Q' be the row and column permutation matrices that combine the permutations for maximum transversal and the block upper triangular form together. Applying these permutations together, we have

$$P'RAQ'Q'^{T}x = P'Rb. \;\; [Q'Q'^{T} = I, the \;\; identity \;\; matrix] \tag{3–3}$$

Let P and Q be row and column permutation matrices that club the P' and Q' mentioned above with the symmetric permutation produced by AMD and the partial pivoting row permutation produced by factorization. Now,

$$PRAQQ^{T}x = PRb$$

or

$$(PRAQ)Q^{T}x = PRb \tag{3–4}$$

The matrix (PRAQ) consists of two parts viz. the diagonal blocks that are factorized and the off-diagonal elements that are not factorized.

$(PRAQ) = LU + F$ where LU represents the factors of all the blocks collectively and F represents the entire off diagonal region. Equation (3–4) now becomes

$$(LU + F)Q^{T}x = PRb \tag{3–5}$$

$$x = Q(LU + F)^{-1}(PRb) \tag{3–6}$$

Equation (3–6) consists of two steps. A block back-substitution i.e. computing $(LU + F)^{-1}(PRb)$ followed by applying the column permutation Q.

The block-back substitution in $(LU + F)^{-1}(PRb)$ looks cryptic and can be better explained as follows: Consider a simple $3 * 3$ block system

$$\begin{bmatrix} L_{11}U_{11} & F_{12} & F_{13} \\ 0 & L_{22}U_{22} & F_{23} \\ 0 & 0 & L_{33}U_{33} \end{bmatrix} * \begin{bmatrix} X_1 \\ X_2 \\ X_3 \end{bmatrix} = \begin{bmatrix} B_1 \\ B_2 \\ B_3 \end{bmatrix} \tag{3–7}$$

The equations corresponding to the above system are:

$$L_{11}U_{11} * X_1 + F_{12} * X_2 + F_{13} * X_3 = B_1 \tag{3–8}$$

$$L_{22}U_{22} * X_2 + F_{23} * X_3 = B_2 \tag{3–9}$$

$$L_{33}U_{33} * X_3 = B_3 \tag{3–10}$$

In block back substitution, we first solve (3–10) for X_3, and then eliminate X_3 from (3–9) and (3–8) using the off-diagonal entries.

Next, we solve (3–9) for X_2 and eliminate X_2 from (3–8).

Finally we solve (3–8) for X_1

3.1 Characteristics of Circuit Matrices

Circuit matrices exhibit certain unique characteristics that makes KLU more relevant to them. They are very sparse. Because of their high sparsity, BLAS kernels are not applicable. Circuit matrices often have a few dense rows/columns that originate from voltage or current sources. These dense rows/columns are effectively removed by BTF permutation.

Circuit matrices are asymmetric, but the nonzero pattern is roughly symmetric. They are easily permutable to block upper triangular form. Besides, they have zero-free or nearly zero-free diagonal. Another peculiar feature of circuit matrices is that the nonzero pattern of each block after permutation to block upper triangular form, is more symmetric than the original matrix. Typical ordering strategies applied to the original matrix cause high fill-in whereas when applied to the blocks, leads to less fill-in.

The efficiency of the permutation to block upper triangular form shows in the fact the entire sub-diagonal region in the matrix has zero work and the off-diagonal elements do not cause any fill-in since they are not factorized.

3.2 Linear Systems in Circuit Simulation

The linear systems in circuit simulation process originate from solving large systems of non linear equations using Newton's method and integrating large stiff systems of ordinary differential equations. These linear systems consist of the coefficients matrix A, the unknowns vector x and the right hand side b.

During the course of simulation, the matrix A retains the same nonzero pattern(structurally unchanged) and only undergoes changes in numerical values. Thus the initial analysis phase (permutation to ensure zero-free diagonal, block triangular form and minimum degree ordering on blocks) and factorization phase(that involves symbolic analysis, partial pivoting and symmetric pruning) can be restricted to the initial system alone.

Subsequent systems $A'x = b$ where only the numerical values of A' are different from A, can be solved using a mechanism called refactorization. Refactorization simply means to use the same row and column permutations (comprising entire analysis phase and partial pivoting) computed for the initial system, for solving the subsequent systems that have changes only in numerical values. Refactorization substantially reduces run time since the analysis time and factorization time spent on symbolic analysis, partial pivoting, pruning are avoided. The nonzero pattern of the factors L and U are the same as for the initial system. Only Numerical factorization using the pre-computed nonzero pattern and partial pivoting order, is required.

The solve step follows the factorization/refactorization step. KLU accomodates solving multiple right hand sides in a single solve step. Upto four right hand sides can be solved in a single step.

3.3 Performance Benchmarks

During my internship at a circuit simulation company, I did performance benchmarking of KLU vs SuperLU in the simulation environment. The performance benchmarks were run on a representative set of examples. The results of these benchmarks are tabulated as follows. (the size of the matrix created in simulation is shown in parenthesis).

Table 3–1: Comparison between KLU and SuperLU on overall time and fill-in

	Overall time			Nonzeros(L+U)	
Netlist	KLU	SuperLU	Speedup	KLU	SuperLU
Problem1 (301)	1.67	1.24	0.74	1808	1968
Problem2 (1879)	734.96	688.52	0.94	13594	13770
Problem3 (2076)	56.46	53.38	0.95	16403	16551
Problem4 (7598)	89.63	81.85	0.91	52056	54997
Problem5 (745)	18.13	16.84	0.93	4156	4231
Problem6 (1041)	1336.50	1317.30	0.99	13198	13995
Problem7 (33)	0.40	0.32	0.80	157	176
Problem8 (10)	4.46	1.570	0.35	40	41
Problem9 (180)	222.26	202.29	0.91	1845	1922
Problem10(6833)	6222.20	6410.40	1.03	56322	58651
Problem11 (1960)	181.78	179.50	0.99	13527	13963
Problem12 (200004)	6.25	8.47	1.35	500011	600011
Problem13 (20004)	0.47	0.57	1.22	50011	60011
Problem14 (40004)	0.97	1.31	1.35	100011	120011
Problem15 (100000)	1.76	2.08	1.18	299998	499932
Problem16 (7602)	217.80	255.88	1.17	156311	184362
Problem17(10922)	671.10	770.58	1.15	267237	299937
Problem18 (14842)	1017.00	1238.10	1.22	326811	425661
Problem19 (19362)	1099.00	1284.40	1.17	550409	581277
Problem20 (24482)	3029.00	3116.90	1.03	684139	788047
Problem21 (30202)	2904.00	3507.40	1.21	933131	1049463

The circuits Problem16–Problem21 are TFT LCD arrays similar to memory circuits. These examples were run atleast twice with each algorithm employed viz. KLU or SuperLU to get consistent results. The results are tabulated in tables 3–1, 3–2 and 3–3. The ”overall time” in table 3–1 comprises of analysis, factorization and solve time.

Table 3–2: Comparison between KLU and SuperLU on factor time and solve time

Netlist	Factor time per iteration		Factor speedup	Solve time per iteration		Solve speedup
	KLU	SuperLU		KLU	SuperLU	
Problem1 (301)	0.000067	0.000084	1.26	0.000020	0.000019	0.92
Problem2 (1879)	0.000409	0.000377	0.92	0.000162	0.000100	0.61
Problem3 (2076)	0.000352	0.000317	0.90	0.000122	0.000083	0.68
Problem4 (7598)	0.001336	0.001318	0.99	0.000677	0.000326	0.48
Problem5 (745)	0.000083	0.000063	0.76	0.000035	0.000022	0.62
Problem6 (1041)	0.000321	0.000406	1.26	0.000079	0.000075	0.95
Problem7 (33)	0.000004	0.000004	0.96	0.000003	0.000002	0.73
Problem8 (10)	0.000001	0.000001	0.89	0.000001	0.000001	0.80
Problem9 (180)	0.000036	0.000042	1.16	0.000014	0.000011	0.76
Problem10(6833)	0.001556	0.001530	0.98	0.000674	0.000365	0.54
Problem11 (1960)	0.000663	0.000753	1.14	0.000136	0.000122	0.90
Problem12 (200004)	0.103900	0.345500	3.33	0.030640	0.041220	1.35
Problem13 (20004)	0.005672	0.020110	3.55	0.001633	0.002735	1.67
Problem14 (40004)	0.014430	0.056080	3.89	0.004806	0.006864	1.43
Problem15 (100000)	0.168700	0.283700	1.68	0.018600	0.033610	1.81
Problem16 (7602)	0.009996	0.017620	1.76	0.001654	0.001439	0.87
Problem17(10922)	0.018380	0.030010	1.63	0.002542	0.001783	0.70
Problem18 (14842)	0.024020	0.046130	1.92	0.003187	0.002492	0.78
Problem19 (19362)	0.054730	0.080280	1.47	0.005321	0.003620	0.68
Problem20 (24482)	0.121400	0.122600	1.01	0.006009	0.004705	0.78
Problem21 (30202)	0.124000	0.188700	1.52	0.009268	0.006778	0.73

3.4 Analyses and Findings

The following are my inferences based on the results: Most of the matrices in these experiments are small matrices of the order of a few thousands.

Fill in is much better with KLU. The 'BTF' ordering combined with the 'AMD' ordering on each of the blocks does a good job in reducing the fill in count to a good extent. The improvement in fill in averages around 6% for many examples.

Table 3–3: Ordering results using BTF+AMD in KLU on circuit matrices

Netlist	Size	Nonzeros in A	Nonzeros in L+U	No of Blocks	Max Block size	Nonzeros off diagonal
Problem1	301	1484	1808	7	295	89
Problem2	1879	12926	13594	19	1861	4307
Problem3	2076	15821	16403	13	2064	6689
Problem4	7598	48922	52056	13	7586	19018
Problem5	745	3966	4156	128	426	1719
Problem6	1041	9654	13198	67	975	2608
Problem7	33	153	157	7	27	50
Problem8	10	39	40	5	6	16
Problem9	180	1503	1845	19	162	661
Problem10	6833	43250	56322	507	6282	12594
Problem11	1960	11187	13527	58	1715	2959
Problem12	200004	500011	500011	200003	2	300005
Problem13	20004	50011	50011	20003	2	30005
Problem14	40004	100011	100011	40003	2	60005
Problem15	100000	299998	299998	1	100000	0
Problem16	7602	32653	156311	103	7500	251
Problem17	10922	46983	267237	123	10800	301
Problem18	14842	63913	326811	143	14700	351
Problem19	19362	83443	550409	163	19200	401
Problem20	24482	105573	684139	183	24300	451
Problem21	30202	130303	933131	203	30000	501

There is no 'fill in' in the Problem12, Problem13, Problem14 and Problem15 netlists with KLU. This is quite significant in that there is no memory overhead in these examples. In the case of circuits Problem16–Problem21, the gain in fill

in with KLU ranges from 6% in the Problem19 example to 24% in Problem18 example.

The gain in fill in translates into faster factorization because few nonzeros imply less work. The factorization time thus is expected to be low. It turns out to be true in most of the cases (factorization speedup of 1.6x in Problem16–Problem21 examples and 3x for Problem12–Problem14 examples). For some cases like Problem2 and Problem10, the factorization time remains same for both KLU and SuperLU.

Solve phase turns out to be slow in KLU. Probably, the off diagonal nonzero handling part tends to account for the extra time spent in the solve phase.

One way of reducing the solve overhead in KLU would be solving multiple RHS at the same time. In a single solve iteration, 4 equations are solved.

On the whole, the overall time speedup is 1.2 for Problem16–Problem21 examples and Problem12–Problem14 examples. For others, the overall time is almost the same between the two algorithms.

BTF is not able to find out many blocks for most of the matrices and there happens to be a single large block and the remaining are singletons. But the AMD ordering does a good job in getting the fill in count reduced. The off-diagonal nonzero count is not high.

3.5 Alternate Ordering Experiments

Different ordering strategies were employed to analyze the fill in behaviour. The statistics using different ordering schemes are listed in table 3–4. 'COLAMD' is not listed in the table. It typically gives poor ordering and causes more fill in than AMD, MMD and AMD+BTF. AMD alone gives relatively higher fill in compared to AMD+BTF in most of the matrices. However AMD alone gives mixed results in comparison with MMD. It matches or outperforms MMD in fill in on Problem12–Problem14 and Problem16–Problem21 matrices. However it gives

Table 3–4: Comparison of ordering results produced by BTF+AMD, AMD, MMD

Netlist	Nonzeros in A	Fill-in BTF+AMD	Fill-in AMD	Fill-in MMD
Problem1 (301)	1484	1808	1928	1968
Problem2 (1879)	12926	13594	13857	13770
Problem3 (2076)	15821	16403	18041	16551
Problem4 (7598)	48922	52056	57975	54997
Problem5 (745)	3966	4156	5562	4231
Problem6 (1041)	9654	13198	14020	13995
Problem7 (33)	153	157	178	176
Problem8 (10)	39	40	41	41
Problem9 (180)	1503	1845	1968	1922
Problem10(6833)	43250	56322	133739	58651
Problem11 (1960)	11187	13527	14800	13963
Problem12 (200004)	500011	500011	600011	600011
Problem13 (20004)	50011	50011	60011	60011
Problem14 (40004)	100011	100011	120011	120011
Problem15 (100000)	299998	299998	299998	499932
Problem16 (7602)	32653	156311	165264	184362
Problem17(10922)	46983	267237	255228	299937
Problem18 (14842)	63913	326811	387668	425661
Problem19 (19362)	83443	550409	451397	581277
Problem20 (24482)	105573	684139	718891	788047
Problem21 (30202)	130303	933131	839226	1049463

poor fill in for rest of the circuits when compared with MMD. AMD alone beats AMD+BTF in fill-in for some of the examples viz. Problem17, Problem19 and Problem21. Overall, to sum it up, BTF+AMD is the best ordering strategy to use.

3.6 Experiments with UF Sparse Matrix Collection

There are a number of circuit matrices in the UF sparse matrix collection. Different experiments were done with these matrices as well on different parameters like

1. ordering quality with different ordering schemes in KLU
2. timing of different phases of KLU
3. ordering quality among KLU, UMFPACK and Gilbert-Peierls' Algorithm
4. performance comparison between KLU and UMFPACK.

UMFPACK is a unsymmetric multifrontal sparse solver and uses an unsymmetric COLAMD ordering or an AMD ordering, automatically selecting which method to use based on the matrix characteristics. Gilbert-Peierls' algorithm is available in MATLAB as an LU factorization scheme. These experiments were done on a Mandrake 10.0 linux OS, Intel Pentium M Processor with clock frequency of 1400 MHz and RAM 768 kB.

3.6.1 Different Ordering Schemes in KLU

There are six different ordering schemes possible in KLU. The three fill reducing schemes are AMD, COLAMD and User Specified Ordering. These three fill reducing orderings can be combined with a BTF preordering or no preordering.Hence six different schemes. However in this experiment user specified ordering is not considered. That leaves us with four different schemes.The table 3–5 lists the fill (number of nonzeros in L+U plus the number of off-diagonal elements) for each of these ordering schemes.

From table 3–5, we find that BTF+AMD gives consistently better fill-in across different circuit matrices. However there are observable aberrations. For

example, with the circuit Bomhof/circuit_2, AMD and COLAMD give better fill-in than BTF+AMD. These results determine again that BTF+AMD is the best ordering scheme to use for circuit matrices.

3.6.2 Timing Different Phases in KLU

These experiments show the time spent in different phases of the algorithm. BTF pre-ordering followed by a AMD fill-reducing ordering is employed. As mentioned earlier, there are four different phases.

1. Analysis phase: This comprises the pre-ordering and fill reducing ordering.
2. Factor phase: This comprises the factorization part of the algorithm. It includes the symbolic analysis phase, partial pivoting, symmetric pruning steps.
3. Refactor phase: This comprises the part where we do a factorization using the already pre-computed partial pivoting permutation and the nonzero pattern of the L and U matrices. There is no symbolic analysis, partial pivoting and symmetric pruning in refactor phase.
4. Solve phase: This comprises the solve phase of the algorithm. Solving a single right hand side was experimented.

When given a set of matrices with the same nonzero pattern, the analysis and factor phases are done only once. The refactor phase is then repeated for the remaining matrices. Solve phase is repeated as many times as there are systems to solve. Table 3–6 consists of the timing results. Analysis phase consumes most of the time spent in the algorithm. Refactor time is typically 3 or 4 times smaller than factor time and 8 times smaller than analysis time plus factor time put together. Solve phase consumes the least fraction of time spent.

3.6.3 Ordering Quality among KLU, UMFPACK and Gilbert-Peierls

The table 3–7 compares the ordering quality among KLU using BTF+AMD, UMFPACK using COLAMD or AMD and Gilbert-Peierls' using AMD. We can

Table 3–5: Fill-in with four different schemes in KLU

Matrix	Nonzeros in A	BTF + AMD	BTF + COLAMD	AMD	COLAMD
Sandia/adder_dcop_01 (1813)	11156	13525	13895	18848	21799
Sandia/adder_trans_01 (1814)	14579	20769	36711	24365	119519
Sandia/fpga_dcop_01 (1220)	5892	7891	8118	8869	12016
Sandia/fpga_trans_01 (1220)	7382	10152	12776	10669	21051
Sandia/init_adder1 (1813)	11156	13525	13895	18848	21799
Sandia/mult_dcop_01 (25187)	193276	226673	228301	2176328	1460322
Sandia/oscil_dcop_01 (430)	1544	2934	3086	3078	3295
Sandia/oscil_trans_01 (430)	1614	2842	3247	2897	3259
Bomhof/circuit_1 (2624)	35823	44879	775363	44815	775720
Bomhof/circuit_2 (4510)	21199	40434	89315	36197	36196
Bomhof/circuit_3 (12127)	48137	86718	98911	245336	744245
Grund/b2_ss (1089)	3895	9994	9212	26971	9334
Grund/b_dyn (1089)	4144	11806	10597	33057	10544
Grund/bayer02 (13935)	63307	889914	245259	1365142	307979
Grund/d_dyn (87)	230	481	461	619	494
Grund/d_ss (53)	144	302	292	382	298
Grund/meg1 (2904)	58142	232042	184471	1526780	378904
Grund/meg4 (5860)	25258	42398	310126	43250	328144
Grund/poli (4008)	8188	12200	12208	12238	12453
Grund/poli_large (15575)	33033	48718	48817	49806	51970
Hamm/add20 (2395)	13151	19554	34636	19554	34636
Hamm/add32 (4960)	19848	28754	36030	28754	36030
Hamm/bcircuit (68902)	375558	1033240	1692668	1033240	1692668
Hamm/hcircuit (105676)	513072	731634	2623852	736080	4425310
Hamm/memplus (17758)	99147	137030	3282586	137030	3282586
Hamm/scircuit (170998)	958936	2481122	6410286	2481832	6427526
Rajat/rajat03 (7602)	32653	163913	235111	172666	236938
Rajat/rajat04 (1041)	8725	12863	80518	18618	158000
Rajat/rajat05 (301)	1250	1926	2053	2101	3131
Rajat/rajat11 (135)	665	890	978	944	1129
Rajat/rajat12 (1879)	12818	15308	273667	15571	128317
Rajat/rajat13 (7598)	48762	58856	90368	64791	5234287
Rajat/rajat14 (180)	1475	1994	2249	2105	2345

Table 3–6: Time in seconds, spent in different phases in KLU

Matrix	Analysis time	Factor time	Refactor time	Solve time
Sandia/adder_dcop_01 (1813)	0.0028	0.0028	0.0007	0.0003
Sandia/adder_trans_01 (1814)	0.0045	0.0038	0.0013	0.0003
Sandia/fpga_dcop_01 (1220)	0.0018	0.0015	0.0004	0.0002
Sandia/fpga_trans_01 (1220)	0.0022	0.0017	0.0005	0.0002
Sandia/init_adder1 (1813)	0.0028	0.0028	0.0007	0.0003
Sandia/mult_dcop_01 (25187)	0.2922	0.0522	0.0196	0.0069
Sandia/oscil_dcop_01 (430)	0.0008	0.0006	0.0002	0.0001
Sandia/oscil_trans_01 (430)	0.0008	0.0006	0.0002	0.0001
Bomhof/circuit_1 (2624)	0.0098	0.0085	0.0053	0.0006
Bomhof/circuit_2 (4510)	0.0082	0.0064	0.0034	0.0006
Bomhof/circuit_3 (12127)	0.0231	0.0174	0.0056	0.0020
Grund/b2_ss (1089)	0.0031	0.0018	0.0005	0.0001
Grund/b_dyn (1089)	0.0033	0.0021	0.0007	0.0002
Grund/bayer02 (13935)	0.0584	0.2541	0.2070	0.0090
Grund/d_dyn (87)	0.0002	0.0001	0.0000	0.0000
Grund/d_ss (53)	0.0001	0.0001	0.0000	0.0000
Grund/meg1 (2904)	0.0178	0.0853	0.0590	0.0033
Grund/meg4 (5860)	0.0157	0.0094	0.0028	0.0009
Grund/poli (4008)	0.0017	0.0010	0.0004	0.0003
Grund/poli_large (15575)	0.0064	0.0045	0.0018	0.0014
Hamm/add20 (2395)	0.0056	0.0044	0.0014	0.0003
Hamm/add32 (4960)	0.0084	0.0074	0.0019	0.0006
Hamm/bcircuit (68902)	0.3120	0.2318	0.1011	0.0257
Hamm/hcircuit (105676)	0.2553	0.1920	0.0658	0.0235
Hamm/memplus (17758)	0.0576	0.0358	0.0157	0.0036
Hamm/scircuit (170998)	0.8491	0.6364	0.3311	0.0622
Rajat/rajat03 (7602)	0.0152	0.0440	0.0306	0.0034
Rajat/rajat04 (1041)	0.0029	0.0023	0.0008	0.0002
Rajat/rajat05 (301)	0.0005	0.0005	0.0001	0.0000
Rajat/rajat11 (135)	0.0002	0.0002	0.0001	0.0000
Rajat/rajat12 (1879)	0.0038	0.0027	0.0008	0.0002
Rajat/rajat13 (7598)	0.0122	0.0105	0.0033	0.0011
Rajat/rajat14 (180)	0.0004	0.0003	0.0001	0.0000

infer from the results that KLU produces better ordering than UMFPACK and Gilbert-Peierls' algorithm. For KLU, the following MATLAB code determines the fill.

```
opts = [0.1 1.2 1.2 10 1 0 0 0 ] ;
[x info] = klus(A,b,opts, []) ;
fill = info (31) + info (32) + info(8) ;
```

For UMFPACK, the snippet is

```
[L U P Q] = lu(A) ;
fill = nnz(L) + nnz(U) ;
```

For Gilbert-Peierls' the code is

```
Q = amd(A) ;
[L U P] = lu(A(Q,Q), 0.1) ;
fill = nnz(L) + nnz(U) ;
```

3.6.4 Performance Comparison between KLU and UMFPACK

This experiment compares the total time spent in the analysis, factor and solve phases by the algorithms. The results are listed in table 3–8. KLU outperforms UMFPACK in time. For KLU, the following snippet in MATLAB is used:

```
tic
[x info] = klus(A,b,opts) ;
t1 = toc ;
```

For UMFPACK, the following code in MATLAB is used to find the total time:

```
tic
x = A \ b ;
t2 = toc ;
```

Table 3–7: Fill-in among KLU, UMFPACK and Gilbert-Peierls

Matrix	nnz	KLU	UMFPACK	Gilbert-Peierls
Sandia/adder_dcop_01 (1813)	11156	13525	14658	18825
Sandia/adder_trans_01 (1814)	14579	20769	20769	24365
Sandia/fpga_dcop_01 (1220)	5892	7891	8106	8869
Sandia/fpga_trans_01 (1220)	7382	10152	10152	10669
Sandia/init_adder1 (1813)	11156	13525	14658	18825
Sandia/mult_dcop_01 (25187)	193276	226673	556746	1300902
Sandia/oscil_dcop_01 (430)	1544	2934	2852	3198
Sandia/oscil_trans_01 (430)	1614	2842	3069	2897
Bomhof/circuit_1 (2624)	35823	44879	44879	44815
Bomhof/circuit_2 (4510)	21199	40434	35107	38618
Bomhof/circuit_3 (12127)	48137	86718	84117	245323
Grund/b2_ss (1089)	3895	9994	8309	22444
Grund/b_dyn (1089)	4144	11806	9642	41092
Grund/bayer02 (13935)	63307	889914	259329	973093
Grund/d_dyn (87)	230	481	442	523
Grund/d_ss (53)	144	302	268	395
Grund/meg1 (2904)	58142	232042	151740	1212904
Grund/meg4 (5860)	25258	42398	42398	43250
Grund/poli (4008)	8188	12200	12200	12239
Grund/poli_large (15575)	33033	48718	48745	49803
Hamm/add20 (2395)	13151	19554	19554	19554
Hamm/add32 (4960)	19848	28754	28754	28754
Hamm/bcircuit (68902)	375558	1033240	1033240	1033240
Hamm/hcircuit (105676)	513072	731634	730906	736080
Hamm/memplus (17758)	99147	137030	137030	137030
Hamm/scircuit (170998)	958936	2481122	2481122	2481832
Rajat/rajat03 (7602)	32653	163913	163913	172666
Rajat/rajat04 (1041)	8725	12863	12860	18613
Rajat/rajat05 (301)	1250	1926	1944	2101
Rajat/rajat11 (135)	665	890	890	944
Rajat/rajat12 (1879)	12818	15308	15308	15571
Rajat/rajat13 (7598)	48762	58856	58856	64791
Rajat/rajat14 (180)	1475	1994	1994	2105

Table 3–8: Performance comparison between KLU and UMFPACK

Matrix	KLU	UMFPACK
Sandia/adder_dcop_01 (1813)	0.0116	0.0344
Sandia/adder_trans_01 (1814)	0.0112	0.0401
Sandia/fpga_dcop_01 (1220)	0.0050	0.0257
Sandia/fpga_trans_01 (1220)	0.0054	0.0203
Sandia/init_adder1 (1813)	0.0109	0.0323
Sandia/mult_dcop_01 (25187)	1.2383	1.1615
Sandia/oscil_dcop_01 (430)	0.0022	0.0070
Sandia/oscil_trans_01 (430)	0.0019	0.0074
Bomhof/circuit_1 (2624)	0.0232	0.1223
Bomhof/circuit_2 (4510)	0.0201	0.0522
Bomhof/circuit_3 (12127)	0.0579	0.1713
Grund/b2_ss (1089)	0.0066	0.0168
Grund/b_dyn (1089)	0.0072	0.0175
Grund/bayer02 (13935)	0.6089	0.3565
Grund/d_dyn (87)	0.0005	0.0014
Grund/d_ss (53)	0.0003	0.0010
Grund/meg1 (2904)	0.1326	0.1018
Grund/meg4 (5860)	0.0571	0.1111
Grund/poli (4008)	0.0050	0.0121
Grund/poli_large (15575)	0.0208	0.0497
Hamm/add20 (2395)	0.0123	0.0506
Hamm/add32 (4960)	0.0201	0.0738
Hamm/bcircuit (68902)	0.7213	1.8823
Hamm/hcircuit (105676)	0.7313	2.7764
Hamm/memplus (17758)	0.1232	0.8140
Hamm/scircuit (170998)	1.9812	7.3448
Rajat/rajat03 (7602)	0.0793	0.1883
Rajat/rajat04 (1041)	0.0068	0.0284
Rajat/rajat05 (301)	0.0014	0.0046
Rajat/rajat11 (135)	0.0007	0.0023
Rajat/rajat12 (1879)	0.0087	0.0355
Rajat/rajat13 (7598)	0.0330	0.1229
Rajat/rajat14 (180)	0.0010	0.0032

CHAPTER 4
USER GUIDE FOR KLU

4.1 The Primary KLU Structures

4.1.1 klu_common

This is a control structure that contains both input control parameters for KLU as well as output statistics computed by the algorithm. It is a mandatory parameter for all the KLU routines. Its contents are listed as follows:

- tol

  Type: double

  Input parameter for pivot tolerance for diagonal preference.Default value is 0.001

- growth

  Type: double

  Input parameter for reallocation growth size of LU factors. Default value is 1.2

- initmem_amd

  Type: double

  Input parameter for initial memory size with AMD.

  Initial memory size = initmem_amd * nnz(L) + n. Default value is 1.2

- initmem

  Type: double

  Input parameter for initial memory size with COLAMD.

  Initial memory size = initmem * nnz(A) + n. Default value is 10

- btf

  Type: int

Input parameter to use BTF pre-ordering, or not. Default value is 1 (to use BTF)

- ordering

  Type: int

  Input parameter to specify which fill reducing ordering to use.

  0= AMD,1= COLAMD, 2= user P and Q. Default is 0.

- scale

  Type: int

  Input parameter to specify which scaling strategy to use. 0= none, 1= sum, 2= max. Default is 0

- singular_proc

  Type: int

  Input parameter to specify whether to stop on singularity or continue.

  0= stop, 1= continue. Default is 0.

- status

  Type: int

  Output parameter that indicates the result of the KLU function call. Values are KLU_OK(0) if OK and < 0 if error. Error values are KLU_SINGULAR (-1), KLU_OUT_OF_MEMORY (-2), KLU_INVALID (-3)

- nrealloc

  Type: int

  Output parameter.Contains number of reallocations of L and U

- singular_col

  Type: int

  Output parameter.Contains the column no of singular column if any

- noffdiag

  Type: int

Output parameter.Contains the number of off-diagonal pivots chosen

4.1.2 klu_symbolic

This structure encapsulates the information related to the analysis phase. The members of the structure are listed as follows:

- symmetry

  Type: double

  Contains the symmetry of largest block

- est_flops

  Type: double

  Contains the estimated factorization flop count

- lnz

  Type: double

  Contains the estimated nonzeros in L including diagonals

- unz

  Type: double

  Contains the estimated nonzeros in U including diagonals

- Lnz

  Type: double *

  Array of size n, but only Lnz [0..nblocks-1] is used. Contains the estimated number of nonzeros in each block

- n

  Type: int

  Contains the size of input matrix A where A is n-by-n

- nz

  Type: int

  Contains the number of entries in input matrix

- P

Type: int *

Array of size n. Contains the row permutation from ordering

- Q

  Type: int *

  Array of size n. Contains the column permutation from ordering

- R

  Type: int *

  Array of size n+1, but only R [0..nblocks] is used. Contains the start and end column/row index for each block. Block k goes from R[k] to R[k+1] - 1

- nzoff

  Type: int

  Contains the number of nonzeros in off-diagonal blocks

- nblocks Type: int

  Contains the number of blocks

- maxblock

  Type: int

  Contains the size of largest block

- ordering

  Type: int

  Contains the ordering used (0 = AMD, 1 = COLAMD, 2 = GIVEN)

- do_btf

  Type: int

  Indicates whether or not BTF preordering was requested

The members symmetry, est_flops, lnz, unz, Lnz are computed only when AMD is used. The remaining members are computed for all orderings.

4.1.3 klu_numeric

This structure encapsulates information related to the factor phase. It contains the LU factors of each block, pivot row permutation, and the entries in the off-diagonal blocks among others. Its contents are listed as follows:

- umin

  Type: double

  Contains the minimum absolute diagonal entry in U

- umax

  Type: double

  Contains the maximum absolute diagonal entry in U

- nblocks

  Type: int

  Contains the number of blocks

- lnz

  Type: int

  Contains actual number of nonzeros in L excluding diagonals

- unz

  Type: int

  Contains actual number of nonzeros in U excluding diagonals

- Pnum

  Type: int *

  Array of size n.Contains the final pivot permutation

- Pinv

  Type: int *

  Array of size n.Contains the inverse of final pivot permutation

- Lbip

  Type: int **

Array of size nblocks. Each element is an array of size block size + 1. Each element contains the column pointers for L factor of the corresponding block

- Ubip

  Type: int **

  Array of size nblocks. Each element is an array of size block size + 1. Each element contains the column pointers for U factor of the corresponding block

- Lblen

  Type: int **

  Array of size nblocks. Each element is an array of size block size. Each element contains the column lengths for L factor of the corresponding block

- Ublen

  Type: int **

  Array of size nblocks. Each element is an array of size block size. Each element contains the column lengths for U factor of the corresponding block

- LUbx

  Type: void **

  Array of size nblocks. Each element is an array containing the row indices and numerical values of LU factors of the corresponding block. The diagonals of LU factors are not stored here

- Udiag

  Type: void **

  Array of size nblocks.Each element is an array of size block size. Each element contains the diagonal values of U factor of the corresponding block

- Singleton

  Type: void *

  Array of size nblocks.Contains the singleton values

- Rs

Type: double *

Array of size n. Contains the row scaling factors

- scale

  Type: int

  Indicates the scaling strategy used.

  (0 = none, 1 = sum, 2 = max)

- Work

  Type: void *

  Permanent workspace used for factorization and solve. It is of size

  MAX (4n numerical values, n numerical values + 6*maxblock int's)

- worksize

  Type: size_t

  Contains the size (in bytes) of Work allocated above

- Xwork

  Type: void *

  This is an alias into Numeric-¿Work

- Iwork

  Type: int *

  An integer alias into Xwork + n

- Offp

  Type: int *

  Array of size n+1. Contains the column pointers for off-diagonal elements.

- Offi

  Type: int *

  Array of size number of off-diagonal entries. Contains the row indices of

  off-diagonal elements

- Offx

Type: void *

Array of size number of off-diagonal entries. Contains the numerical values of off-diagonal elements

4.2 KLU Routines

The user callable KLU routines in the C language are explained in this section. The following guidelines are applicable to all routines except when explicitly stated otherwise in the description of a routine.

1. : All the arguments are required except when explicitly stated as optional. If optional, a user can pass NULL for the corresponding argument.
2. : The control input/output argument "Common" of type "klu_common *" is a required argument for all routines.
3. : All arguments other than the above mentioned control argument "Common", are input arguments and are not modified.

4.2.1 klu_analyze

```
klu_symbolic *klu_analyze
(
    int n,
    int Ap [ ],
    int Ai [ ],
    klu_common *Common
) ;
```

This routine orders the matrix using BTF if specified and the fill reducing ordering specified.

- Returns a pointer to klu_symbolic structure that contains the ordering information.
- All arguments are required
  - n: Size of the input matrix A where A is n*n.
  - Ap: Array of column pointers for the input matrix. Size n+1.

- Ai: Array of row indices. Size number of nonzeros in A.
- Common: The control input/output structure.

4.2.2 klu_analyze_given

```
klu_symbolic *klu_analyze_given
(
    int n,
    int Ap [ ],
    int Ai [ ],
    int Puser [ ],
    int Quser [ ],
    klu_common *Common
) ;
```

This routine orders the matrix using BTF if specified and the given Puser and QUser as fill reducing ordering. If Puser and Quser are NULL, then the natural ordering is used.

- Returns a pointer to klu_symbolic structure that contains the ordering information.
- Arguments
  - n: Size of the input matrix A where A is n*n. Required.
  - Ap: Array of column pointers for the input matrix. Size n+1. Required.
  - Ai: Array of row indices. Size number of nonzeros in A. Required.
  - Puser: Optional row permutation to use.
  - Quser: Optional column permutation to use.
  - Common: The control input/output structure.

4.2.3 klu_*factor

```
klu_numeric *Numeric klu_factor
(
    int Ap [ ],
    int Ai [ ],
```

```
    double Ax [ ],
    klu_symbolic *Symbolic,
    klu_common *Common
) ;
```

This routine factors a real matrix. There is a complex version of this routine klu_z_factor that factors a complex matrix and has same function declaration as klu_factor. Both use the results of a call to klu_analyze.

- Returns a pointer to klu_numeric if successful. NULL otherwise.
- All the arguments are required.
  - Ap: Array of column pointers for the input matrix. Size n+1.
  - Ai: Array of row indices. Size number of nonzeros in A.
  - Ax: Array of numerical values. Size number of nonzeros in A. In the complex case, the array should consist of real and imaginary parts of each numerical value as adjacent pairs.
  - Symbolic: The structure that contains the results from a call to klu_analyze.
  - Common: The control input/output structure. The status field in Common is set to indicate if the routine was successful or not.

4.2.4 klu_*solve

```
void klu_solve
(
    klu_symbolic *Symbolic,
    klu_numeric *Numeric,
    int ldim,
    int nrhs,
    double B [ ],
    klu_common *Common
) ;
```

This routine solves a real system. There is a complex version of this routine klu_z_solve, that solves a complex system and has the same function declaration as klu_solve. Both use the results of a call to klu_analyze and klu_*factor.

- Return type is void. The rhs vector B is overwritten with the solution.
- All Arguments are required.
  - Symbolic: The structure that contains the results from a call to klu_analyze.
  - Numeric: The structure that contains the results from a call to klu_*factor.
  - ldim: The leading dimension of the right hand side B.
  - nrhs: The number of right hand sides being solved.
  - B: The right hand side. It is a vector of length ldim * nrhs. It can be real or complex depending on whether a real or complex system is being solved. If complex, the real and imaginary parts of the rhs numerical value must be stored as adjacent pairs. It is overwritten with the solution.
  - Common: The control input/output structure.

4.2.5 klu_*tsolve

```
void klu_tsolve
(
    klu_symbolic *Symbolic,
    klu_numeric *Numeric,
    int ldim,
    int nrhs,
    int conj_solve,
    double B [ ],
    klu_common *Common
) ;
```

This routine is similar to klu_solve except that it solves a transpose system $A'x = b$. This routine solves a real system. Again there is a complex version of this routine klu_z_tsolve for solving complex systems and has the same function declaration as klu_tsolve. It also offers to do a conjugate transpose solve for the complex system $A^H x = b$.

- Return type is void. The rhs vector B is overwritten with the solution.
- All arguments are required. The descriptions for all arguments except conj_solve are same as those for klu_*solve. The argument conj_solve is relevant only for complex case. It takes two values 1 = CONJUGATE TRANSPOSE SOLVE, 0 = TRANSPOSE SOLVE.

4.2.6 klu_*refactor

```
void klu_refactor
(
    int Ap [ ],
    int Ai [ ],
    double Ax [ ],
    klu_symbolic *Symbolic,
    klu_numeric *Numeric,
    klu_common *Common
) ;
```

This routine does a refactor of the matrix using the previously computed ordering information in Symbolic and the nonzero pattern of the LU factors in Numeric objects. It assumes same partial pivoting order computed in Numeric. It changes only the numerical values of the LU factors stored in Numeric object. It has a complex version klu_z_refactor with the same function prototype to handle complex cases.

- Return type is void. The numerical values of LU factors in Numeric parameter are updated.
- All arguments are required.

- Ap: Array of column pointers for the input matrix. Size n+1.
- Ai: Array of row indices. Size number of nonzeros in A.
- Ax: Array of numerical values. Size number of nonzeros in A. In the complex case, the array should consist of real and imaginary parts of each numerical value as adjacent pairs.
- Symbolic: The structure that contains the results from a call to klu_analyze.
- Numeric: Input/output argument. The structure contains the results from a call to klu_*factor. The numerical values of LU factors are overwritten with the ones for the current matrix being factorized.
- Common: The control input/output structure. The status field in Common is set to indicate if the routine was successful or not.

4.2.7 klu_defaults

```
void klu_defaults
(
    klu_common *Common
) ;
```

This routine sets the default values for the control input parameters of the klu_common object. The default values are listed in the description of the klu_common structure. A call to this routine is required unless the user sets the control input parameters explicitly.

- Return type is void.
- The argument Common is required. The control input parameters in Common are set to default values.

4.2.8 klu_*rec_pivot_growth

```
double klu_rec_pivot_growth
(
    int Ap [ ],
```

```
    int Ai [ ],
    double Ax [ ],
    klu_symbolic *Symbolic,
    klu_numeric *Numeric,
    klu_common *Common
) ;
```

This routine computes the reciprocal pivot growth of the factorization algorithm. The complex version of this routine klu_z_rec_pivot_growth handles complex matrices and has the same function declaration.

- The pivot growth estimate is returned.
- All arguments are required.
  - Ap: Array of column pointers for the input matrix. Size n+1.
  - Ai: Array of row indices. Size number of nonzeros in A.
  - Ax: Array of numerical values. Size number of nonzeros in A. In the complex case, the array should consist of real and imaginary parts of each numerical value as adjacent pairs.
  - Symbolic: The structure that contains the results from a call to klu_analyze.
  - Numeric: The structure that contains the results from a call to klu_*factor.
  - Common: The control input/output structure. The status field in Common is set to indicate if the routine was successful or not.

4.2.9 klu_*estimate_cond_number

```
double klu_estimate_cond_number
(
    int Ap [ ],
    double Ax [ ],
    klu_symbolic *Symbolic,
    klu_numeric *Numeric,
```

```
    klu_common *Common
);
```

This routine computes the condition number estimate of the input matrix. As before, the complex version of this routine klu_z_estimate_cond_number has the same function declaration and handles complex matrices.

- The condition number estimate is returned.
- All arguments are required.
  - Ap: Array of column pointers for the input matrix. Size n+1.
  - Ax: Array of numerical values. Size number of nonzeros in A. In the complex case, the array should consist of real and imaginary parts of each numerical value as adjacent pairs.
  - Symbolic: The structure that contains the results from a call to klu_analyze.
  - Numeric: The structure that contains the results from a call to klu_*factor.
  - Common: The control input/output structure. The status field in Common is set to indicate if the routine was successful or not.

4.2.10 klu_free_symbolic

```
void klu_free_symbolic
(
     klu_symbolic **Symbolic,
     klu_common *Common
) ;
```

This routine deallocates or frees the contents of the klu_symbolic object. The Symbolic parameter must be a valid object computed by a call to klu_analyze or klu_analyze_given.

- Return type is void.
- All arguments are required.

- Symbolic: Input/Output argument. Must be a valid object computed by a call to klu_analyze or klu_analyze_given. If NULL, the routine just returns.
- Common: The control input/output structure.

4.2.11 klu_free_numeric

```
void klu_free_numeric
(
     klu_numeric **Numeric,
     klu_common *Common
) ;
```

This routine frees the klu_numeric object computed by a call to klu_factor or klu_z_factor routines. It resets the pointer to klu_numeric to NULL. There is a complex version of this routine called klu_z_free_numeric with the same function declaration to handle the complex case.

- Return type is void.
- All arguments are required.
  - Numeric. Input/Output argument. The contents of the klu_numeric object are freed. The pointer to klu_numeric object is set to NULL.
  - Common: The control input/output structure.

REFERENCES

[1] C. L. Lawson, R. J. Hanson, D. Kincaid, and F. T. Krogh, Basic linear algebra subprograms for FORTRAN usage, *ACM Trans. Math. Soft.*, 5: 308–323, 1979.

[2] J. J. Dongarra, J. Du Croz, S. Hammarling, and R. J. Hanson, An extended set of FORTRAN basic linear algebra subprograms, *ACM Trans. Math. Soft.*, 14: 1–17, 1988.

[3] J. J. Dongarra, J. Du Croz, S. Hammarling, and R. J. Hanson, Algorithm 656: An extended set of FORTRAN basic linear algebra subprograms, *ACM Trans. Math. Soft.*, 14: 18–32, 1988.

[4] J. J. Dongarra, J. Du Croz, I. S. Duff, and S. Hammarling, A set of level 3 basic linear algebra subprograms, *ACM Trans. Math. Soft.*, 16: 1–17, 1990.

[5] J. J. Dongarra, J. Du Croz, I. S. Duff, and S. Hammarling, Algorithm 679: A set of level 3 basic linear algebra subprograms, *ACM Trans. Math. Soft.*, 16: 18–28, 1990.

[6] James W. Demmel, Stanley C. Eisenstat, John R. Gilbert, Xiaoye S. Li and Joseph W. H. Liu, A supernodal approach to sparse partial pivoting, *SIAM J. Matrix Analysis and Applications*, 20(3): 720–755, 1999.

[7] Timothy A. Davis, I.S.Duff, An unsymmetric-pattern multifrontal method for sparse LU factorization, *SIAM J. Matrix Analysis and Applic.*, 18(1): 140–158, 1997.

[8] Timothy A. Davis, Algorithm 832: UMFPACK, an unsymmetric-pattern multifrontal method with a column pre-ordering strategy, *ACM Trans. Math. Software*, 30(2): 196–199, 2004.

[9] John R.Gilbert and Tim Peierls, Sparse partial pivoting in time proportional to arithmetic operations, *SIAM J. Sci. Stat. Comput.*, 9(5): 862–873, 1988.

[10] A. George and E. Ng, An implementation of Gaussian elimination with partial pivoting for sparse systems. *SIAM J. Sci. Statist. Comput.*, 6(2): 390–409, 1985.

[11] Iain S. Duff, On algorithms for obtaining a maximum transversal, *ACM Transactions on Mathematical Software*, 7(3): 315–330, 1981.

[12] Iain S. Duff, Algorithm 575 permutations for a zero-free diagonal, *ACM Transactions on Mathematical Software*, 7(3): 387–390, 1981.

[13] Iain S. Duff and John K. Reid, Algorithm 529: permutations to block triangular form, *ACM Trans. on Mathematical Software*, 4(2): 189–192, 1978.

[14] Iain S. Duff and John K. Reid, An implementation of Tarjan's algorithm for the block triangular form of a matrix, *ACM Trans. on Mathematical Software*, 4(2): 137–147, 1978.

[15] R.E. Tarjan, Depth first search and linear graph algorithms, *SIAM J. Computing.*, 1: 146–160, 1972.

[16] Thomas H. Cormen, Charles E. Leiserson, Ronald L. Rivest, Clifford Stein, *Introduction to Algorithms*, Second Edition 2001, MIT Press, Cambridge.

[17] S.C. Eisenstat and J.W.H. Liu, Exploiting structural symmetry in a sparse partial pivoting code, *SIAM J.Sci.Comput.*, 14(1): 253–257, 1993.

[18] P. R. Amestoy, T. A. Davis, and I. S. Duff,An approximate minimum degree ordering algorithm, *SIAM J. Matrix Anal. Applic.*, 17(4): 886–905, 1996.

[19] P.R. Amestoy, T.A. Davis, and I.S. Duff, Algorithm 837: AMD, an approximate minimum degree ordering algorithm, *ACM Transactions on Mathematical Software*, 30(3): 381–388, 2004.

[20] Timothy A. Davis, John R. Gilbert, Stefan I. Larimore, and Esmond G. Ng. A column approximate minimum degree ordering algorithm, *ACM Transactions on Mathematical Software*, 30(3): 353–376, 2004.

[21] Timothy A. Davis , John R. Gilbert , Stefan I. Larimore , Esmond G. Ng, Algorithm 836: COLAMD, a column approximate minimum degree ordering algorithm, *ACM Transactions on Mathematical Software*, 30(3): 377–380, 2004.

[22] W.W. Hager, Condition estimates, *SIAM J. Sci. Stat. Comput.*, 5,2: 311–316, 1984.

[23] Nicholas J. Higham, Fortran codes for estimating the one-norm of a real or complex matrix, with applications to condition estimation, *ACM Trans. on Mathematical Software.*, 14(4): 381–396, 1988.

BIOGRAPHICAL SKETCH

Ekanathan was born in Tirunelveli, India, on October 2, 1977. He received his Bachelor of Technology degree in chemical engineering from Anna University, Chennai, India, in May 1998. He worked with Infosys Technologies at Brussels, Belgium, as programmer/analyst from 1998 till 2001 and with SAP AG at Walldorf, Germany, as software developer from 2001 till 2003. Currently he is pursuing his Master of Science degree in computer science at University of Florida. His interests and hobbies include travel, reading novels and magazines, music and movies.
