# Supplementary material for: Inference for Stochastic Chemical Kinetics Using Moment Equations and System Size Expansion
Source: PLoS Comput Biol. 2016 Jul 22;12(7):e1005030. doi: 10.1371/journal.pcbi.1005030 (PMC4957800; doi:10.1371/journal.pcbi.1005030)
Supplement: S1 Code — This zip-file contains the MATLAB code for the simulation and application example presented in the paper. We provide implementations of all models, parameter estimation and uncertainty analysis to allow everybody to reproduce the results. (ZIP) [file pcbi.1005030.s002.zip › S1code/cvodewrap/sundials-2.6.1/INSTALL_GUIDE.pdf]

Installation Guide for SUNDIALS v2.6.1

Eddy Banks, Aaron M. Collier, Alan C. Hindmarsh, Radu Serban, and Carol S. Woodward
Center for Applied Scientific Computing
Lawrence Livermore National Laboratory

March 30, 2015

UCRL-SM-208116

DISCLAIMER

This document was prepared as an account of work sponsored by an agency of the United States government. Neither the United States government nor Lawrence Livermore National Security, LLC, nor any of their employees makes any warranty, expressed or implied, or assumes any legal liability or responsibility for the accuracy, completeness, or usefulness of any information, apparatus, product, or process disclosed, or represents that its use would not infringe privately owned rights. Reference herein to any specific commercial product, process, or service by trade name, trademark, manufacturer, or otherwise does not necessarily constitute or imply its endorsement, recommendation, or favoring by the United States government or Lawrence Livermore National Security, LLC. The views and opinions of authors expressed herein do not necessarily state or reflect those of the United States government or Lawrence Livermore National Security, LLC, and shall not be used for advertising or product endorsement purposes.

This work was performed under the auspices of the U.S. Department of Energy by Lawrence Livermore National Laboratory under Contract DE-AC52-07NA27344.

Approved for public release; further dissemination unlimited

Contents

1 SUNDIALS Package Installation Procedure **1**

1.1 CMake-based installation 2

1.1.1 Configuring, building, and installing on Unix-like systems 2

1.1.2 Configuration options (Unix/Linux) 4

1.1.3 Configuration examples 7

1.1.4 Working with external Libraries 7

1.2 Building and Running Examples 8

1.3 Configuring, building, and installing on Windows 8

1.4 Installed libraries and exported header files 9

Chapter 1

SUNDIALS Package Installation Procedure

The installation of any SUNDIALS package is accomplished by installing the SUNDIALS suite as a whole, according to the instructions that follow. The same procedure applies whether or not the downloaded file contains one or all solvers in SUNDIALS.

The SUNDIALS suite (or individual solvers) are distributed as compressed archives (`.tar.gz`). The name of the distribution archive is of the form *solver*-`x.y.z.tar.gz`, where *solver* is one of: `sundials`, `cvode`, `cvodes`, `arkode`, `ida`, `idas`, or `kinsol`, and `x.y.z` represents the version number (of the SUNDIALS suite or of the individual solver) . To begin the installation, first uncompress and expand the sources, by issuing

```
% tar xzf solver-x.y.z.tar.gz
```

This will extract source files under a directory *solver*-`x.y.z`.

Starting with version `2.6.0` of SUNDIALS, CMake is the only supported method of installation. The explanations on the installation procedure begins with a few common observations:

- The remainder of this chapter will follow these conventions:

  srcdir is the directory *solver*-`x.y.z` created above; i.e., the directory containing the SUNDIALS sources.

  builddir is the (temporary) directory under which SUNDIALS is built.

  instdir is the directory under which the SUNDIALS exported header files and libraries will be installed. Typically, header files are exported under a directory *instdir*`/include` while libraries are installed under *instdir*`/lib`, with *instdir* specified at configuration time.

- For SUNDIALS CMake-based installation, in-source builds are prohibited; in other words, the build directory *builddir* can **not** be the same as *srcdir* and such an attempt will lead to an error. This prevents "polluting" the source tree and allows efficient builds for different configurations and/or options.

- The installation directory *instdir* can **not** be the same as the source directory *srcdir*.

- By default, only the libraries and header files are exported to the installation directory *instdir*. If enabled by the user (with the appropriate toggle for CMake), the examples distributed with SUNDIALS will be built together with the solver libraries but the installation step will result in exporting (by default in a subdirectory of the installation directory) the example sources and sample outputs together with automatically generated configuration files that reference the *installed* SUNDIALS headers and libraries. As such, these configuration files for the SUNDIALS examples can be used as "templates" for your own problems. CMake installs `CMakeLists.txt` files and also (as an option available only under Unix/Linux) `Makefile` files. Note this installation

approach also allows the option of building the SUNDIALS examples without having to install them. (This can be used as a sanity check for the freshly built libraries.)

- Even if generation of shared libraries is enabled, only static libraries are created for the FCMIX modules. (Because of the use of fixed names for the Fortran user-provided subroutines, FCMIX shared libraries would result in "undefined symbol" errors at link time.)

1.1 CMake-based installation

CMake-based installation provides a platform-independent build system. CMake can generate Unix and Linux Makefiles, as well as KDevelop, Visual Studio, and (Apple) XCode project files from the same configuration file. In addition, CMake also provides a GUI front end and which allows an interactive build and installation process.

The SUNDIALS build process requires CMake version `2.8.1` or higher and a working compiler. On Unix-like operating systems, it also requires Make (and `curses`, including its development libraries, for the GUI front end to CMake, `ccmake`), while on Windows it requires Visual Studio. While many Linux distributions offer CMake, the version included is probably out of date. Many new CMake features have been added recently, and you should download the latest version from `http://www.cmake.org`. Build instructions for CMake (only necessary for Unix-like systems) can be found on the CMake website. Once CMake is installed, Linux/Unix users will be able to use `ccmake`, while Windows users will be able to use `CMakeSetup`.

As previously noted, when using CMake to configure, build and install SUNDIALS, it is always required to use a separate build directory. While in-source builds are possible, they are explicitly prohibited by the SUNDIALS CMake scripts (one of the reasons being that, unlike autotools, CMake does not provide a `make distclean` procedure and it is therefore difficult to clean-up the source tree after an in-source build). By ensuring a separate build directory, it is an easy task for the user to clean-up all traces of the build by simply removing the build directory. CMake does generate a `make clean` which will remove files generated by the compiler and linker.

1.1.1 Configuring, building, and installing on Unix-like systems

The default CMake configuration will build all included solvers and associated examples and will build static and shared libraries. The *installdir* defaults to `/usr/local` and can be changed by setting the `CMAKE_INSTALL_PREFIX` variable. Support for FORTRAN and all other options are disabled.

CMake can be used from the command line with the `cmake` command, or from a `curses`-based GUI by using the `ccmake` command. Examples for using both methods will be presented. For the examples shown it is assumed that there is a top level SUNDIALS directory with appropriate source, build and install directories:

```
% mkdir (...)sundials/instdir
% mkdir (...)sundials/builddir
% cd (...)sundials/builddir
```

Building with the GUI

Using CMake with the GUI follows this general process:

- Select and modify values, run configure (`c` key)
- New values are denoted with an asterisk
- To set a variable, move the cursor to the variable and press enter
  - If it is a boolean (ON/OFF) it will toggle the value
  - If it is string or file, it will allow editing of the string

– For file and directories, the <tab> key can be used to complete

- Repeat until all values are set as desired and the generate option is available (`g` key)
- Some variables (advanced variables) are not visible right away
- To see advanced variables, toggle to advanced mode (`t` key)
- To search for a variable press `/` key, and to repeat the search, press the `n` key

To build the default configuration using the GUI, from the *builddir* enter the ccmake command and point to the *srcdir*:

```
% ccmake ../srcdir
```

The default configuration screen is shown in Figure 1.1.

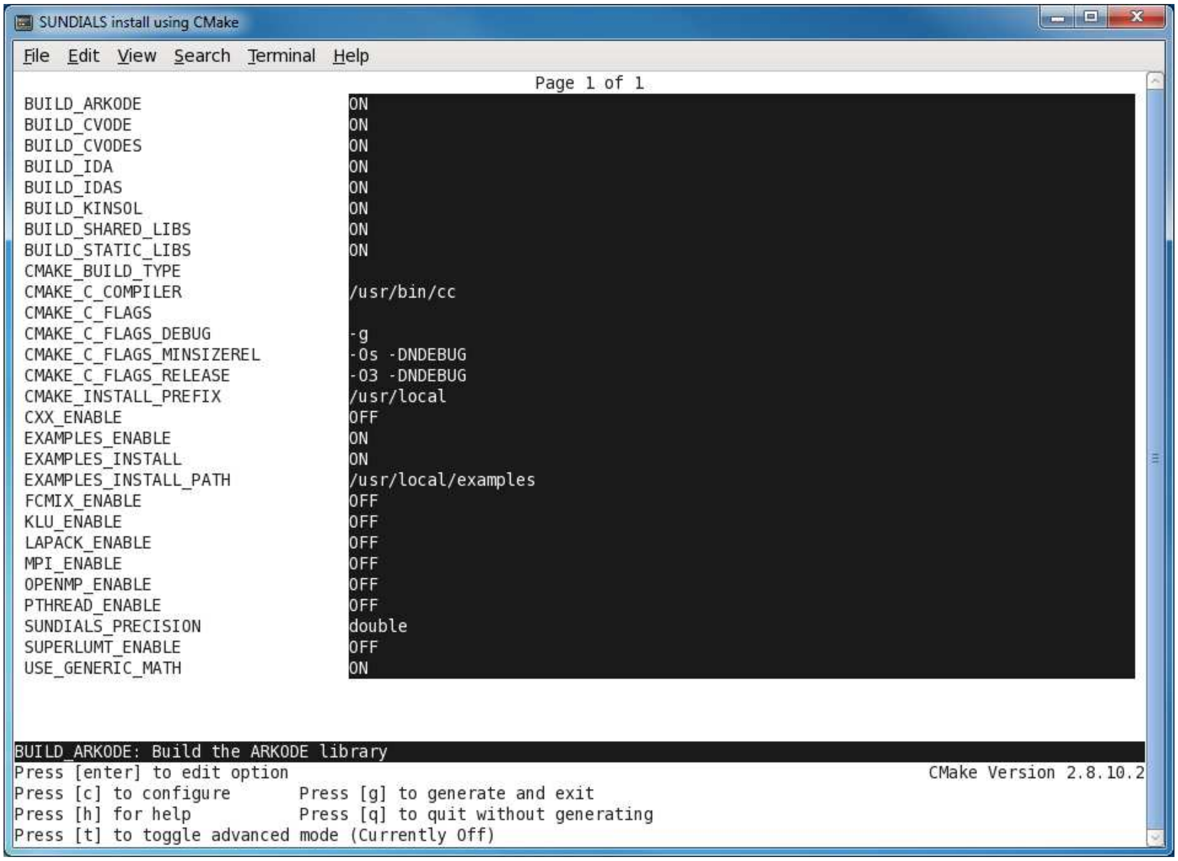

Figure 1.1: Default configuration screen. Note: Initial screen is empty. To get this default configuration, press 'c' repeatedly (accepting default values denoted with asterisk) until the 'g' option is available.

The default *instdir* for both SUNDIALS and corresponding examples can be changed by setting the `CMAKE_INSTALL_PREFIX` and the `EXAMPLES_INSTALL_PATH` as shown in figure 1.2.

Pressing the (`g` key) will generate makefiles including all dependencies and all rules to build SUNDIALS on this system. Back at the command prompt, you can now run:

```
% make
```

To install SUNDIALS in the installation directory specified in the configuration, simply run:

```
% make install
```

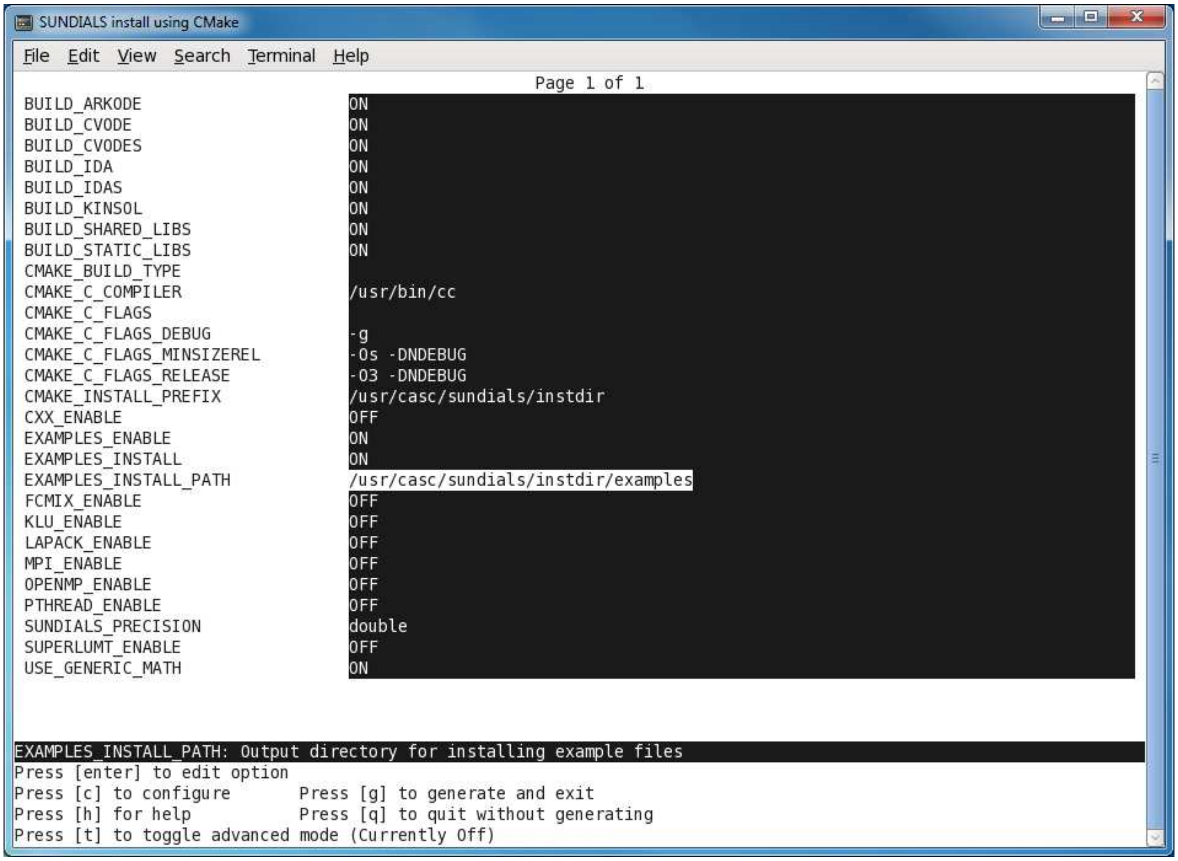

Figure 1.2: Changing the *instdir* for SUNDIALS and corresponding examples

Building from the command line

Using CMake from the command line is simply a matter of specifying CMake variable settings with the cmake command. The following will build the default configuration:

```
% cmake -DCMAKE_INSTALL_PREFIX=/home/myname/sundials/instdir \
> -DEXAMPLES_INSTALL_PATH=/home/myname/sundials/instdir/examples \
> ../srcdir
% make
% make install
```

1.1.2 Configuration options (Unix/Linux)

A complete list of all available options for a CMake-based SUNDIALS configuration is provide below. Note that the default values shown are for a typical configuration on a Linux system and are provided as illustration only.

BUILD_ARKODE - Build the ARKODE library
    Default: ON

BUILD_CVODE - Build the CVODE library
    Default: ON

BUILD_CVODES - Build the CVODES library
    Default: ON

BUILD_IDA - Build the IDA library
Default: ON

BUILD_IDAS - Build the IDAS library
Default: ON

BUILD_KINSOL - Build the KINSOL library
Default: ON

BUILD_SHARED_LIBS - Build shared libraries
Default: OFF

BUILD_STATIC_LIBS - Build static libraries
Default: ON

CMAKE_BUILD_TYPE - Choose the type of build, options are: None (CMAKE_C_FLAGS used) Debug Release RelWithDebInfo MinSizeRel
Default:

CMAKE_C_COMPILER - C compiler
Default: /usr/bin/cc

CMAKE_C_FLAGS - Flags for C compiler
Default:

CMAKE_C_FLAGS_DEBUG - Flags used by the compiler during debug builds
Default: -g

CMAKE_C_FLAGS_MINSIZEREL - Flags used by the compiler during release minsize builds
Default: -Os -DNDEBUG

CMAKE_C_FLAGS_RELEASE - Flags used by the compiler during release builds
Default: -O3 -DNDEBUG

CMAKE_Fortran_COMPILER - Fortran compiler
Default: /usr/bin/gfortran
Note: Fortran support (and all related options) are triggered only if either Fortran-C support is enabled (FCMIX_ENABLE is ON) or Blas/Lapack support is enabled (LAPACK_ENABLE is ON).

CMAKE_Fortran_FLAGS - Flags for Fortran compiler
Default:

CMAKE_Fortran_FLAGS_DEBUG - Flags used by the compiler during debug builds
Default:

CMAKE_Fortran_FLAGS_MINSIZEREL - Flags used by the compiler during release minsize builds
Default:

CMAKE_Fortran_FLAGS_RELEASE - Flags used by the compiler during release builds
Default:

CMAKE_INSTALL_PREFIX - Install path prefix, prepended onto install directories
Default: /usr/local
Note: The user must have write access to the location specified through this option. Exported SUNDIALS header files and libraries will be installed under subdirectories include and lib of CMAKE_INSTALL_PREFIX, respectively.

EXAMPLES_ENABLE - Build the SUNDIALS examples
Default: ON

EXAMPLES_INSTALL - Install example files
Default: ON
Note: This option is triggered only if building example programs is enabled (EXAMPLES_ENABLE ON). If the user requires installation of example programs then the sources and sample output files for all SUNDIALS modules that are currently enabled will be exported to the directory specified by EXAMPLES_INSTALL_PATH. A CMake configuration script will also be automatically generated and exported to the same directory. Additionally, if the configuration is done under a Unix-like system, makefiles for the compilation of the example programs (using the installed SUNDIALS libraries) will be automatically generated and exported to the directory specified by EXAMPLES_INSTALL_PATH.

EXAMPLES_INSTALL_PATH - Output directory for installing example files
Default: /usr/local/examples
Note: The actual default value for this option will an `examples` subdirectory created under CMAKE_INSTALL_PREFIX.

FCMIX_ENABLE - Enable Fortran-C support
Default: OFF

KLU_ENABLE - Enable KLU support
Default: OFF

LAPACK_ENABLE - Enable Lapack support
Default: OFF
Note: Setting this option to ON will trigger the two additional options see below.

LAPACK_LIBRARIES - Lapack (and Blas) libraries
Default: /usr/lib/liblapack.so;/usr/lib/libblas.so
Note: CMake will search for these libraries in your LD_LIBRARY_PATH prior to searching default system paths.

MPI_ENABLE - Enable MPI support
Default: OFF
Note: Setting this option to ON will trigger several additional options related to MPI.

MPI_MPICC - `mpicc` program
Default:

MPI_RUN_COMMAND - Specify run command for MPI
Default: mpirun
Note: This can either be set to `mpirun` for OpenMPI or `srun` if jobs are managed by SLURM - Simple Linux Utility for Resource Management as exists on LLNL's high performance computing clusters.

MPI_MPIF77 - `mpif77` program
Default:
Note: This option is triggered only if using MPI compiler scripts (MPI_USE_MPISCRIPTS is ON) and Fortran-C support is enabled (FCMIx_ENABLE is ON).

OPENMP_ENABLE - Enable OpenMP support
Default: OFF
Turn on support for the OpenMP based nvector.

PTHREAD_ENABLE - Enable Pthreads support
Default: OFF
Turn on support for the Pthreads based nvector.

SUNDIALS_PRECISION - Precision used in SUNDIALS, options are: double, single or extended
Default: double

SUPERLUMT_ENABLE - Enable SUPERLU_MT support
Default: OFF

USE_GENERIC_MATH - Use generic (stdc) math libraries
Default: ON

1.1.3 Configuration examples

The following examples will help demonstrate usage of the CMake configure options.
To configure SUNDIALS using the default C and Fortran compilers, and default mpicc and mpif77 parallel compilers, enable compilation of examples, and install libraries, headers, and example sources under subdirectories of /home/myname/sundials/, use:

```
% cmake \
> -DCMAKE_INSTALL_PREFIX=/home/myname/sundials/instdir \
> -DEXAMPLES_INSTALL_PATH=/home/myname/sundials/instdir/examples \
> -DMPI_ENABLE=ON \
> -DFCMIX_ENABLE=ON \
> /home/myname/sundials/srcdir
%
% make install
%
```

To disable installation of the examples, use:

```
% cmake \
> -DCMAKE_INSTALL_PREFIX=/home/myname/sundials/instdir \
> -DEXAMPLES_INSTALL_PATH=/home/myname/sundials/instdir/examples \
> -DMPI_ENABLE=ON \
> -DFCMIX_ENABLE=ON \
> -DEXAMPLES_INSTALL=OFF \
> /home/myname/sundials/srcdir
%
% make install
%
```

1.1.4 Working with external Libraries

The SUNDIALS Suite contains many options to enable implementation flexibility when developing solutions. The following are some notes addressing specific configurations when using the supported third party libraries.

Building with LAPACK and BLAS

To enable LAPACK and BLAS libraries, set the LAPACK_ENABLE option to ON. If the directory containing the LAPACK and BLAS libraries is in the LD_LIBRARY_PATH environment variable, CMake will set the LAPACK_LIBRARIES variable accordingly, otherwise CMake will attemp to find the LAPACK and BLAS libraries in standard system locations. To explicitly tell CMake what libraries to use, the LAPACK_LIBRARIES varible can be set to the desired libraries. Example:

```
% cmake \
> -DCMAKE_INSTALL_PREFIX=/home/myname/sundials/instdir \
> -DEXAMPLES_INSTALL_PATH=/home/myname/sundials/instdir/examples \
> -DLAPACK_LIBRARIES=/mypath/lib/liblapack.so;/mypath/lib/libblas.so \
> /home/myname/sundials/srcdir
%
% make install
%
```

Building with KLU

The KLU libraries are part of SuiteSparse, a suite of sparse matrix software, available from the Texas A&M University website: `http://faculty.cse.tamu.edu/davis/suitesparse.html`. SUNDIALS has been tested with SuiteSparse version 4.2.1. To enable KLU, set `KLU_ENABLE` to `ON`, set `KLU_INCLUDE_DIR` to the `include` path of the KLU installation and set `KLU_LIBRARY_DIR` to the `lib` path of the KLU installation. The CMake configure will result in populating the following variables: `AMD_LIBRARY`, `AMD_LIBRARY_DIR`, `BTF_LIBRARY`, `BTF_LIBRARY_DIR`, `COLAMD_LIBRARY`, `COLAMD_LIBRARY_DIR`, and `KLU_LIBRARY`

Building with SuperLU_MT

The SuperLU_MT libraries are available for download from the Lawrence Berkeley National Laboratory website: `http://crd-legacy.lbl.gov/~xiaoye/SuperLU/#superlu_mt`. SUNDIALS has been tested with SuperLU_MT version 2.4. To enable SuperLU_MT, set `SUPERLUMT_ENABLE` to `ON`, set `SUPERLUMT_INCLUDE_DIR` to the `SRC` path of the SuperLU_MT installation, and set the variable `SUPERLUMT_LIBRARY_DIR` to the `lib` path of the SuperLU_MT installation. At the same time, the variable `SUPERLUMT_THREAD_TYPE` must be set to either `Pthread` or `OpenMP`.

Do not mix thread types when building SUNDIALS solvers. If threading is enabled for SUNDIALS by having either `OPENMP_ENABLE` or `PTHREAD_ENABLE` set to `ON` then SuperLU_MT should be set to use the same threading type.

1.2 Building and Running Examples

Each of the SUNDIALS solvers is distributed with a set of examples demonstrating basic usage. To build and install the examples, set both `EXAMPLES_ENABLE` and `EXAMPLES_INSTALL` to `ON`. Specify the installation path for the examples with the variable `EXAMPLES_INSTALL_PATH`. CMake will generate `CMakeLists.txt` configuration files (and `Makefile` files if on Linux/Unix) that reference the *installed* SUNDIALS headers and libraries.

Either the `CMakeLists.txt` file or the traditional `Makefile` may be used to build the examples as well as serve as a template for creating user developed solutions. To use the supplied `Makefile` simply run `make` to compile and generate the executables. To use CMake, from within the installed example directory, run `cmake` (or `ccmake` to use the GUI) followed by `make` to compile the example code. Note that if CMake is used, it will overwrite the traditional `Makefile` with a new CMake generated `Makefile`. The resulting output from running the examples can be compared with example output bundled in the SUNDIALS distribution.

NOTE: There will potentially be differences in the output due to machine architecture, compiler versions, use of third party libraries etc.

1.3 Configuring, building, and installing on Windows

CMake can also be used to build SUNDIALS on Windows. To build SUNDIALS for use with Visual Studio the following steps should be performed:

1. Unzip the downloaded tar file(s) into a directory, this will be the *srcdir*
2. Create a separate *builddir*
3. Open a Visual Studio Command Prompt and cd to *builddir*
4. Run cmake-gui ../*srcdir*
   (a) Hit Configure
   (b) Check/Uncheck solvers to be built
   (c) Change CMAKE_INSTALL_PREFIX to *instdir*

(d) Set other options as desired

(e) Hit Generate

5. Back in the VS Command Window:

   (a) Run msbuild ALL_BUILD.vcxproj

   (b) Run msbuild INSTALL.vcxproj

The resulting libraries will be in the *instdir*. The SUNDIALS project can also now be opened in Visual Studio. Double click on the ALL_BUILD.vcxproj file to open the project. Build the whole *solution* to create the SUNDIALS libraries. To use the SUNDIALS libraries in your own projects, you must set the include directories for your project, add the SUNDIALS libraries to your project solution, and set the SUNDIALS libraries as dependencies for your project.

1.4 Installed libraries and exported header files

Using the CMake SUNDIALS build system, the command

```
% make install
```

will install the libraries under *libdir* and the public header files under *includedir*. The values for these directories are *instdir*/`lib` and *instdir*/`include`, respectively. The location can be changed by setting the CMake variable `CMAKE_INSTALL_PREFIX`. Although all installed libraries reside under *libdir*/`lib`, the public header files are further organized into subdirectories under *includedir*/`include`.

The installed libraries and exported header files are listed for reference in Tables 1.1 and 1.2. The file extension *.lib* is typically `.so` for shared libraries and `.a` for static libraries. Note that, in the Tables, names are relative to *libdir* for libraries and to *includedir* for header files.

A typical user program need not explicitly include any of the shared SUNDIALS header files from under the *includedir*/`include/sundials` directory since they are explicitly included by the appropriate solver header files (*e.g.*, `cvode_dense.h` includes `sundials_dense.h`). However, it is both legal and safe to do so, and would be useful, for example, if the functions declared in `sundials_dense.h` are to be used in building a preconditioner.

Table 1.1: SUNDIALS libraries and header files

SHARED	Libraries	n/a	
	Header files	sundials/sundials_config.h	sundials/sundials_types.h
		sundials/sundials_math.h	
		sundials/sundials_nvector.h	sundials/sundials_fnvector.h
		sundials/sundials_direct.h	sundials/sundials_lapack.h
		sundials/sundials_dense.h	sundials/sundials_band.h
		sundials/sundials_sparse.h	
		sundials/sundials_iterative.h	sundials/sundials_spgmr.h
		sundials/sundials_spbcgs.h	sundials/sundials_sptfqmr.h
		sundials/sundials_pcg.h	sundials/sundials_spfgmr.h
NVECTOR_SERIAL	Libraries	libsundials_nvecserial.*lib*	libsundials_fnvecserial.a
	Header files	nvector/nvector_serial.h	
NVECTOR_PARALLEL	Libraries	libsundials_nvecparallel.*lib*	libsundials_fnvecparallel.a
	Header files	nvector/nvector_parallel.h	
NVECTOR_OPENMP	Libraries	libsundials_nvecopenmp.*lib*	libsundials_fnvecopenmp.a
	Header files	nvector/nvector_openmp.h	
NVECTOR_PTHREADS	Libraries	libsundials_nvecpthreads.*lib*	libsundials_fnvecpthreads.a
	Header files	nvector/nvector_pthreads.h	
CVODE	Libraries	libsundials_cvode.*lib*	libsundials_fcvode.a
	Header files	cvode/cvode.h	cvode/cvode_impl.h
		cvode/cvode_direct.h	cvode/cvode_lapack.h
		cvode/cvode_dense.h	cvode/cvode_band.h
		cvode/cvode_diag.h	
		cvode/cvode_sparse.h	cvode/cvode_klu.h
		cvode/cvode_superlumt.h	
		cvode/cvode_spils.h	cvode/cvode_spgmr.h
		cvode/cvode_sptfqmr.h	cvode/cvode_spbcgs.h
		cvode/cvode_bandpre.h	cvode/cvode_bbdpre.h
CVODES	Libraries	libsundials_cvodes.*lib*	
	Header files	cvodes/cvodes.h	cvodes/cvodes_impl.h
		cvodes/cvodes_direct.h	cvodes/cvodes_lapack.h
		cvodes/cvodes_dense.h	cvodes/cvodes_band.h
		cvodes/cvodes_diag.h	
		cvodes/cvodes_sparse.h	cvodes/cvodes_klu.h
		cvodes/cvodes_superlumt.h	
		cvodes/cvodes_spils.h	cvodes/cvodes_spgmr.h
		cvodes/cvodes_sptfqmr.h	cvodes/cvodes_spbcgs.h
		cvodes/cvodes_bandpre.h	cvodes/cvodes_bbdpre.h
ARKODE	Libraries	libsundials_arkode.*lib*	libsundials_farkode.a
	Header files	arkode/arkode.h	arkode/arkode_impl.h
		arkode/arkode_direct.h	arkode/arkode_lapack.h
		arkode/arkode_dense.h	arkode/arkode_band.h
		arkode/arkode_sparse.h	arkode/arkode_klu.h
		arkode/arkode_superlumt.h	
		arkode/arkode_spils.h	arkode/arkode_spgmr.h
		arkode/arkode_sptfqmr.h	arkode/arkode_spbcgs.h
		arkode/arkode_pcg.h	arkode/arkode_spfgmr.h
		arkode/arkode_bandpre.h	arkode/arkode_bbdpre.h

Table 1.2: SUNDIALS libraries and header files (cont.)

IDA	Libraries	libsundials_ida.*lib*	libsundials_fida.a
	Header files	ida/ida.h	ida/ida_impl.h
		ida/ida_direct.h	ida/ida_lapack.h
		ida/ida_dense.h	ida/ida_band.h
		ida/ida_sparse.h	ida/ida_klu.h
		ida/ida_superlumt.h	
		ida/ida_spils.h	ida/ida_spgmr.h
		ida/ida_spbcgs.h	ida/ida_sptfqmr.h
		ida/ida_bbdpre.h	
IDAS	Libraries	libsundials_idas.*lib*	
	Header files	idas/idas.h	idas/idas_impl.h
		idas/idas_direct.h	idas/idas_lapack.h
		idas/idas_dense.h	idas/idas_band.h
		idas/idas_sparse.h	idas/idas_klu.h
		idas/idas_superlumt.h	
		idas/idas_spils.h	idas/idas_spgmr.h
		idas/idas_spbcgs.h	idas/idas_sptfqmr.h
		idas/idas_bbdpre.h	
KINSOL	Libraries	libsundials_kinsol.*lib*	libsundials_fkinsol.a
	Header files	kinsol/kinsol.h	kinsol/kinsol_impl.h
		kinsol/kinsol_direct.h	kinsol/kinsol_lapack.h
		kinsol/kinsol_dense.h	kinsol/kinsol_band.h
		kinsol/kinsol_sparse.h	kinsol/kinsol_klu.h
		kinsol/kinsol_superlumt.h	
		kinsol/kinsol_spils.h	kinsol/kinsol_spgmr.h
		kinsol/kinsol_spbcgs.h	kinsol/kinsol_sptfqmr.h
		kinsol/kinsol_bbdpre.h	kinsol/kinsol_spfgmr.h
